# Identification and characterization of miRNAs expressed in the bovine ovary

**DOI:** 10.1186/1471-2164-10-443

**Published:** 2009-09-18

**Authors:** Md Munir Hossain, Nasser Ghanem, Michael Hoelker, Franca Rings, Chirawath Phatsara, Ernst Tholen, Karl Schellander, Dawit Tesfaye

**Affiliations:** 1 Institute of Animal Science, Animal Breeding and Husbandry Group, University of Bonn, Endenicher Allee 15, 53115 Bonn, Germany

## Abstract

**Background:**

MicroRNAs are the major class of gene-regulating molecules playing diverse roles through sequence complementarity to target mRNAs at post-transcriptional level. Tightly regulated expression and interaction of a multitude of genes for ovarian folliculogenesis could be regulated by these miRNAs. Identification of them is the first step towards understanding miRNA-guided gene regulation in different biological functions. Despite increasing efforts in miRNAs identification across various species and diverse tissue types, little is known about bovine ovarian miRNAs. Here, we report the identification and characterization of miRNAs expressed in the bovine ovary through cloning, expression analysis and target prediction.

**Results:**

The miRNA library (5'-independent ligation cloning method), which was constructed from bovine ovary in this study, revealed cloning of 50 known and 24 novel miRNAs. Among all identified miRNAs, 38 were found to be new for bovine and were derived from 43 distinct loci showing characteristic secondary structure. While 22 miRNAs precursor loci were found to be well conserved in more than one species, 16 were found to be bovine specific. Most of the miRNAs were cloned multiple times, in which let-7a, let-7b, let-7c, miR-21, miR-23b, miR-24, miR-27a, miR-126 and miR-143 were cloned 10, 28, 13, 4, 11, 7, 6, 4 and 11 times, respectively. Expression analysis of all new and some annotated miRNAs in different intra-ovarian structures and in other multiple tissues showed that some were present ubiquitously while others were differentially expressed among different tissue types. Bta-miR-29a was localized in the follicular cells at different developmental stages in the cyclic ovary. Bio-informatics prediction, screening and Gene Ontology analysis of miRNAs targets identified several biological processes and pathways underlying the ovarian function.

**Conclusion:**

Results of this study suggest the presence of miRNAs in the bovine ovary, thereby elucidate their potential role in regulating diverse molecular and physiological pathways underlying the ovarian functionality. This information will give insights into bovine ovarian miRNAs, which can be further characterized for their role in follicular development and female fertility as well.

## Background

Folliculogenesis is the result of series of complex and coordinated processes, which include morphological and functional changes in different types of follicular cells and their interactions. Sequential recruitment, selection and growth of the follicles, atresia, ovulation and luteolysis are dynamically regulated events that occur on a cyclical basis within the ovary. These processes are under control of closely coordinated endocrine and paracrine factors to develope a number of ovulatory follicles that are species and breed dependent [1]. All those events entail substantial changes and balance between many processes such as the cell cycle, cellular growth, proliferation, differentiation, angiogenesis, steroidogenesis and atresia to determine the ultimate fate of follicles. All of these steady state cyclic changes are controlled by tightly regulated expression and interaction of a multitude of genes in different compartments of the ovary (oocyte, cumulus granulosa, mural granulosa cells and theca cells) to facilitate oocyte development [2].

In oogenesis and embryo development, there are different mechanisms regulating gene expression at the post-transcriptional level. These include events of mRNA adenylation and deadenylation, the CAP structure at the 5' end of the mRNA and the effective action of mRNA binding factors [3,4]. Recently, a new post-transcriptional gene regulation is opened up after promising discovery of hundreds of miRNAs in different mammalian species. Diverse expression pattern of miRNAs and high number of their potential target mRNAs suggests their involvement in the regulation of various developmentally related genes at post-transcriptional level [5-11]. The tiny (18-24 nt in length) and single-stranded miRNAs are derived from primary transcripts termed as "pri-miRNAs", which have an RNA hairpin structure of 60-120 nt with a mature miRNA in one of the two strands. This hairpin in turn is cleaved from the pri-miRNA in the nucleus by the double-strand-specific ribonuclease, Drosha [12]. The resulting precursor miRNA (or pre-miRNA) is transported to the cytoplasm via a process that involves Exportin-5 [13] and subsequently cleaved by Dicer [14] to generate a short, double-stranded (ds) RNA duplex. One of the strands of the miRNA duplex is incorporated into a protein complex termed RNA induced silencing complex (RISC). RISC is guided by the incorporated miRNA strand to mRNAs containing complementary sequences in 3' untranslated region, which primarily results in inhibition of mRNA translation [15]. Those mRNAs which are repressed by miRNAs are further stored in the cytoplasmic foci called P-bodies [16-18].

Several studies have shown the involvement of miRNAs in animal development. Inhibition of miRNA biogenesis has resulted in developmental arrest in mouse and fish [19-21]. Similarly, loss of important miRNA processing machinery, Dicer1 resulted in female infertility in mouse [22,23]. Targeted knockdown of mir-17-5p and let-7p in wild type mice revealed impaired corpus luteum (CL) angiogenesis and decreased serum progesterone levels. In the same study, injection of these miRNAs revealed the restoration of vasculature within the CL and increased progesterone levels [23]. In addition to loss-of-function approach, efforts have been done to identity miRNAs by cloning. For example, small RNA-cDNA libraries from the ovaries of 2-wk-old and adult mice have generated a number of miRNAs with potential role in ovarian function [24]. Subsequent study on ovarian miRNAs in mouse showed the post-transcriptional regulation of CtBP1 gene by miR-132 and miR-212 in cultured granulosa cells [25]. In addition to miRNA, several other non-coding small RNAs including rapiRNAs, napiRNAs, rasiRNAs and tncRNAs are identified and reported in different species [26-28].

Bio-informatic approaches and construction of small RNA-cDNA libraries from bovine adipose tissue, mammary gland, embryo, thymus, small intestine, mesenteric lymph node and abomasum lymph node have identified most of presently annotated bovine miRNAs [29,30]. The number of bovine miRNAs (117) in comparison to Human (695), Chicken (475), Mouse (488), Chimpanzee (595), Rhesus Monkey (463) in miRBase 12.0 are inadequate to disclose global miRNAs regulation of gene expression for various biological functions and disease conditions. Recently, we have shown the dynamics of miRNAs expression during bovine oocyte maturation in vitro using heterologous approach [31]. This together with previous report in mouse supports the possible role of miRNAs during follicular development and oocyte growth. Identifying entire set as well as ovary-specific miRNAs may lead to understanding miRNA-guided gene regulation in the ovary. So, the present study has been conducted to get insight into the miRNA population present in bovine ovary by investigating their characteristics, expression pattern and features of their target genes.

## Results

### Description of the bovine ovarian small RNA library

**Figure 1:**
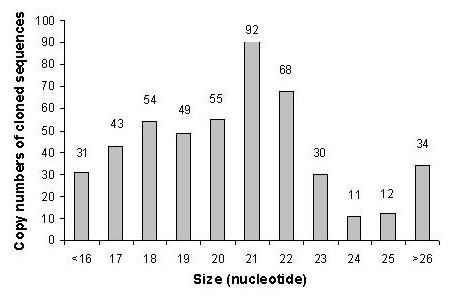
**Size distribution of 479 small RNAs sequences cloned from the bovine ovary**.

**Figure 2:**
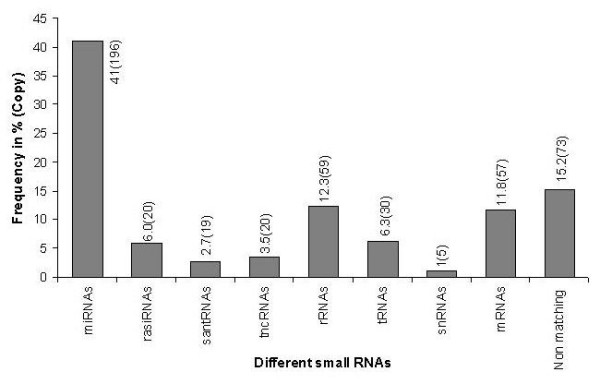
**Frequency (%) of different types of RNA represented in the library**.

To identify miRNAs in the ovary, RNAs of 18 to 26 nt in length from bovine ovarian small RNAs (~200 nt) were purified, cloned, sequenced and analyzed. About 233 concatemer clones were sequenced to generate 479 sequences (after discarding non-quality and self ligated linker sequences). Of these 80 small RNA-cDNA sequences were beyond the expected range of nucleotides (18-26nt) in length. Only sequences of 18 nt or more in length were subjected to detail analysis. Distribution of different lengths of nucleotide sequences found in this library is presented in figure 1. We categorized all identified sequences according to their properties as determined by in-silico analysis based on the criteria reported elsewhere for different types of small RNAs [26,27,32,33]. The 479 sequences identified in the library represented 41% miRNAs, 12% mRNA, 12% rRNA, 6.3% tRNA, 6.0% repeat associated siRNA, 2.7% small antisense RNA, 3.5% tiny noncoding RNA, 1% small nuclear RNA and 15.2% sequences that did not match to bovine genome (Figure 2).

### Distinct miRNAs identified in the bovine ovary

In cDNA library a total of 196 sequences were found to be miRNA like molecules, of which 74 revealed distinct miRNAs (Table 1, Additional file 1). Out of these 74 miRNAs, 36 were found to be reported in miRBase 12.0 for different species including bovine, 14 are registered only for other species and 24 were completely new. Of these 38 new bovine miRNAs, 15 miRNAs were identical or differed by only one or two nucleotides from known mammalian miRNAs. We denoted all the new miRNAs starting with prefix 'bomir' followed by their homologue miRNA number or by clone name in case of no sequence homology. Already annotated miRNAs were named as they were stated in miRBase.

**Table 1:** List of new miRNAs cloned from bovine ovary

**miR ID**	**Length**	**Homolog**	**Copy**	**Strand**	**Sequence**	**Genomic Location^e^**	**Transcript**
bomir-22*/22-5p^a^	22	hsa-miR-22	3	+/-	ACAGUUCUUCA ACUGGCAGCUU	19:22901905: 22901926:1^f^	miR trans.
bomir140*/140-5p^b^	22	hsa-miR-140	1	+	CAGUGGUUUUA CCCUAUGGUAG	18:35987052: 35987073:1^f^	miR trans.
bomir-143-3p	22	ggo-miR-143	11	+/-	UGAGAUGAAGC ACUGUAGCUCG	7:60268857: 60268878:1^f^	Intergenic
bomir-152-5p	21	hsa-miR-152	1	-	CCAAGUUCUG UCAUGCACUGA	19:39650399: 39650419:-1^f^	Intragenic
bomir-193a-2-3p^c^	19	bta-miR193a	1	-	GGGACUUUGU AGGCCAGUU	14:889828: 889846:-1^f^	Intronic
bomir-378-1-3p	21	hsa-miR-378	1	+	CUGGACUUGGA GUCAGAAGGC	7:60536513: 60536533:1^f^	Intronic
bomir-378-2-5p	21	hsa-miR-378	- -	+	CUGGACUUGG AGUCAGAAGGC	4:11116898: 11116918:1^h^	Intronic
bomir-382-3p	22	hsa-miR-382	1	-	GAAUCCACCAC GAACAACUUC	21:66031757: 66031777:-1^f^	Intronic
bomir-409-5p	22	hsa-miR-409	2	-	GGGGUUCACCG AGCAACAUUC	21:66042162: 66042182:-1^f^	Intronic
bomir-424-3p	22	hsa-miR-424*	1	-	CAAAACGUGAG GCGCUGCUAU	Un.04.53:446874: 446894:-1^f^	Intronic
bomir-503-3p	23	mmu-miR-503	1	+	UGCAGUACUGU UCCCGCUGCUA	Un.004.53:446563: 446584:1^f^	Intergenic
bomir-542-3p	23	hsa-miR-542	1	+	UCUCGUGACAU GAUGAUCCCCGA	Un.004.53:441604: 441626:1^f^	Intergenic
bomir-574-5p	22	hsa-miR-574	1	-	UGUGGGUGUGU GCAUGUGCGUG	16:59370677: 59370698:-1^f^	Intergenic
bomir-652-3p^d^	21	hsa-miR-652	5	+	CACAACCCTA GTGGCGCCATT	(from *H. sap*.)	-----
bomir-940-5p	18	hsa-miR-940	1	-	GCAGGGCCC CCGCUCCCC	20:75274475: 75274492:-1^h^	Intergenic
bomir-F0131-5p	18	mmu-miR-667	1	+	GGGGCGGGG GGGCGGGUG	7:10905965: 10905982:1^h^	Intergenic
bomir-F0132-5p	19	hsa-miR-1469	1	+	AGCCCGGGC CCCUCCCCUG	7:13891718: 13891736:1^h^	Intragenic
bomir-H0121-3p	19	hsa-miR-1471	1	+	CUUCCCGUG UGUUGAGCC	18:7202610: 7202627:1^h^	Intergenic
bomir-F0244-5p	19	osa-miR1423	1	-	GCUACUACC GAUUGGAUGG	12:45758300: 45758318:-1^g^	Intergenic
bomir-H0222-3p	22	cre-miR1172.1	1	-	GGACGGCGGCA GCGCCGGGGCG	29:41706141: 41706159:-1^f^	Intergenic
bomir-A0321-3p	18	mml-miR-638	1	+	AGCGCCGCC GGCCGCACC	19:39110507: 39110524:1^g^	Intronic
bomir-C0533-5p	20	oan-miR-1418*	1	+	CGGGACCGGG GUCCGGUGCG	18:59928733: 59928752:1^f^	Intergenic
						21:52041918: 52041937:-1^f^	Intergenic
bomir-F0522-1-3p	19	hsa-miR-1234	1	+	GGUGGGGUGG GGGGGUUGG	21:35870379: 35870397:1^h^	Intergenic
						22:59347395: 59347413:1^h^	Intronic
bomir-B0821-5p	21	oan-miR-1394	1	-	GUCCCCGGGGC UCCCGCCGGC	20:19373746: 19373766:-1^h^	Intergenic
bomir-F1351-3p	20	gga-miR-1607	3	+	GCCCCGGCCG CUCCCGGCCU	25:41129497: 41129516:1^h^	Intergenic
bomir-F1353-5p	20	dre-miR-430c	1	+	AUCUUUGGGC UAGGUUAGUU	28:27885036: 27885055:1^h^	Intronic
bomir-D1431-5p	22	pta-miR1310	2	-	GGCGACGGAGG CGCGACCCCCC	12:75102030: 75102051:-1^g^	Intergenic
bomir-C1511-5p	20	hsa-miR-877	1	+	GUGGAGGAGA AUGCCCGGGG	Un.04.1059:20639: 20658:1^h^	Intronic
bomir-F1821-3p	21	hsa-miR-631	1	+	AGCCCUGGCCC UGCCAUCGUG	Un.04.152:123191: 123211:1^h^	Intronic
bomir-C1931-5p	23	gma-miR1523	1	+	CCUGCUGAUCU CACAUUAAUUCA	26:12405838: 12405860:1^h^	Intergenic
bomir-A2143-3p	18	oan-miR-181c*	1	+	CGGCAGAUG AAGUCCAUC	16:47801336: 47801353:1^h^	Intronic
bomir-F2422-5p	20	hsa-miR-659	1	+	GGUGGGAGGG UCCCACCGAG	18:53584142: 53584161:1^h^	Intragenic
bomir-F2531-3p	18	ppt-miR1030i	3	+	UGGUGGAGA UGCCGGGGA	8:77307661: 77307678:1^g^	Intergenic
bomir-G2511-3p	18	bmo-miR-92	1	+	AGGCGGGCC GGGGUUGGA	18:41190536: 41190553:1^h^	Intergenic
bomir-E2664-3p	20	mml-miR-638	1	-	AGGGCGGGCG GCGACUGGAA	18:64361001: 64361020:-1^h^	Intragenic
bomir-D3011-3p	21	mml-miR-650b	1	+	CCGAGUGCUC CCGCGAGCGCU	18:39424938: 39424958:1^g^	Intragenic
bomir-A3341-1-3p	22	bta-miR-487a	1	+	GUGGCUGUCCC UGGAGGUGGG	3:124988008: 124988028:1^h^	Intergenic
						Un.04.4799:1335: 1355:1^h^	Intergenic
bomir-A3711-5p	19	hsa-miR-937	2	+	UUCCGCGCUC UACGCCAGC	9:63475804: 63475822:1^g^	Intergenic
bomir-A4052-1-5p	19	hsa-miR-615	1	+	GGGAGCCUCG GUUGGCCUC	18:59928630: 59928648:1^f^	Intergenic
						21:52042022: 52042040:-1^f^	Intragenic
						Un.04.2732:16069: 16087:-1^f^	Intergenic

^a^: Cloned sequence is homolog to has-miR 22 but not to bta-miR-22, may bta-miR-22 presented in miRBase v. 12 is bta-miR-22*, ^b^: Cloned sequence is homologue to has-miR-140 but not to bta-miR-140, may bta-miR-140 presented in miRBase v. 12 is bta-miR-140*

^c^: Sequence is smaller than bta-miR-193a and has different genomic locus. ^d^: Sequence does not match to bovine genome, ^e^: Genomic location presenting chromosome number with start and end position along with sense/antisense orientation by 1/-1 of cloned mature sequence. Conservation pattern of the predicted precursor sequences from flanking bovine genome sequence is indicated by- ^f^: found in more than 6 mammalian species, ^g^: present at least in 2 species, ^h^: only in bovine.

**Figure 3:**
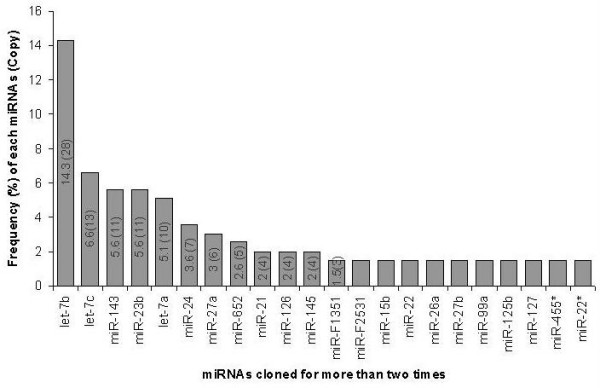
**Frequency (%) of cloned miRNAs along with their copy number**.

Two miRNAs, namely: mir-22/22* and 140/140* which are cloned from 5' fold back arm of the hairpin precursor, have shown exact match to human miRNAs but not to bovine as annotated in miRBase. So, previously annotated bta-miR-22 and 140 seem to be miR-22* and miR-140*, respectively. The number of times that each miRNA cloned in the library ranged from 28 clones for let-7b to a single clone (singleton) for 39 of the 73 miRNAs. All in all, 22 of the 73 miRNAs were cloned for three or more times (Figure 3, Additional file 1).

The corresponding bovine genomic sequences and their locations were identified for each miRNA. The 5' or 3' flanking genomic sequences were then tested for the ability to fold into canonical ~70-nt miRNA precursor hairpin structures by using the MFOLD web server [34]. Small RNA clones with proper positioning within an arm of the hairpin suggest that they have been excised during dicer processing in the cells. Nearly in all of those cases, sequences were found to be conserved in different species including the predicted precursors (Additional file 2). The Bomir-652, which could not be located in bovine genome, was found to be cloned for five times in the library and share sequence homology with already identified miRNA in other species.

### Genomic distribution, properties and clustering of new miRNAs

Genomic locations and properties of the new miRNAs are shown in table 1. All newly identified bovine miRNAs (except bomir-652) are corresponded to 43 distinct loci. Putative precursor hairpin structures have been predicted for all these 43 loci using genomic sequences flanked from candidate miRNAs (Additional file 2). Thirty three of these are found to be encoded by single copy miRNA genes, whereas the other five (bomir-378, bomir-C0533-5p, bomir-F0522-3p, bomir-A3341-3p and bomir-A4052-5p) have multiple loci in the bovine genome (Additional file 2). The analysis of the genomic positions of 61 sequences corresponding to 38 distinct new miRNA genes showed that the majority (23 out of 44 loci) are localized to intergenic regions and the rest corresponded to the intragenic regions in either sense or antisense orientation (Additional file 2). However, 11 sequences are found to be exclusively from known intronic region.

Characterization of our miRNAs was done based on the annotation in the bovine genome data base Ensembl 52: Btau_4.0 [35]. Bomir-F0522-3p and bomir-A4052-5p were mapped to both intergenic and intronic locations. Bomir-F0132-5p (sense), bomir-E2664-3p (antisense) and bomir-A4052-5p (antisense) are originated from the exons of protein-coding genes. While searching the genomic location for all miRNAs, we found six new genomic locations for annotated miRNAs like bta-mir-106, 24, 26, 199a and let-7b (Additional file 1).

All the 50 new genomic loci were found to be distributed in 19 chromosomes (Chr.) namely: Chr. 3, 4, 5, 7, 8, 9, 11, 12, 14, 16, 18, 19, 20, 21, 22, 25, 26, 28 and 29. However, eight loci were found to be mapped to unknown chromosome in the Ensembl 52: Btau_4.0 (end note). Among all newly identified loci, eight miRNA genes were found to be located on Chr. 18 and five miRNAs found on Chr. 7 and 21. Further analysis of the already annotated miRNAs and the newly predicted loci has revealed six miRNAs gene clusters which were mapped within < 10 kb. This clusters are i) bta-miR-10a and bomiR-A0321 on Chr. 19; ii) bta-miR-23b, bta-miR-27b and bta-miR-24-3 on Chr. 8; iii) bta-let-7a-3 and let 7b-2-3P on Chr. 5; iv) bomiR-A4052-1 and bomiR-C0533 on Chr. 18; v) bta-miR-487a, bta-miR-487-b, bomiR-382 and bomiR-409 on Chr. 21; vi) bomiR-C0533-2 and bomiR-A4052-2 on Chr. 21.

To determine whether our new miRNAs are conserved among closely related species, we have searched for homology for precursor sequence in the ENSEMBL genome databases. Results revealed that 17 precursor loci (out of 43 loci for 38 new bovine miRNAs) were found to be conserved in at least six species. While five miRNAs (bomiR-F0244, bomiR-A0321, bomiR-F2531, bomiR-D3011 and bomiR-A3711) were found to be conserved in at least two species, 21 miRNA loci were specific to bovine. All of the newly cloned miRNAs were found to be conserved as mature sequences in the genome of different species. Thermo-dynamically stable hairpin structures were found for those conserved and new miRNAs as shown in additional file 2.

### Other small RNAs and their genomic properties found in the library

Analysis of small RNA library in the present study has enabled us to identify 57 different endogenous siRNAs. We categorized them broadly into two groups, namely: 29 sequences composed of 27 distinct RNAs derived from genomic repetitive region as repeat associated small interfering RNAs (rasiRNAs) and other 30 RNAs associated to non repetitive regions as non-repeat associated small interfering RNAs (nasiRNAs). According to their sequence properties 13 out of 30 nasiRNAs were found to be natural antisense transcripts with ~20 nt in length. Therefore, since they seem to be endogenous siRNAs, we denoted them as small antisense RNAs (santRNAs) and the rest 17 as tiny non-coding RNAs (tncRNAs). Size ranges for rasiRNAs were 18-28 nt (with mean ± SD 21.5 ± 3.1 nt), which did not revealed a sharp size distribution characteristic. However, for the santRNAs and tncRNAs the size distribution was 19.6 ± 1.9 and 19.5 ± 1.1 nt, respectively. Cloned rasiRNAs were found to be distributed on various chromosomes and mapped to repeat sequences mostly corresponding to retrotransposons in both sense and antisense orientation. Total numbers of hits for 27 rasiRNAs were 581 (ranging from 4 to100). Seventy five percent of the rasiRNAs were found to have preference for uridine and adenine residues in either 3' or 5' end position. While seven of the santRNAs were precisely mapped to intergenic region, six fitted to intronic region. All the 13 santRNAs were cloned as antisense orientation to the genome or intron of the protein coding genes on 12 different chromosomes.

Secondary structure analysis of all santRNAs revealed no characteristic hairpin as found for the miRNAs. While eleven tncRNAs were mapped to intergenic region, five were mapped to intronic and two to exonic regions. Two of the seventeen tncRNAs were predicted to form potential fold back structures like the miRNAs. However, these putative tncRNA precursor structures deviated significantly from the miRNA hairpins in key features and they were found to be poorly conserved in closely related species.

### Detection and expression of miRNAs in the ovary and other bovine tissues

**Figure 4:**
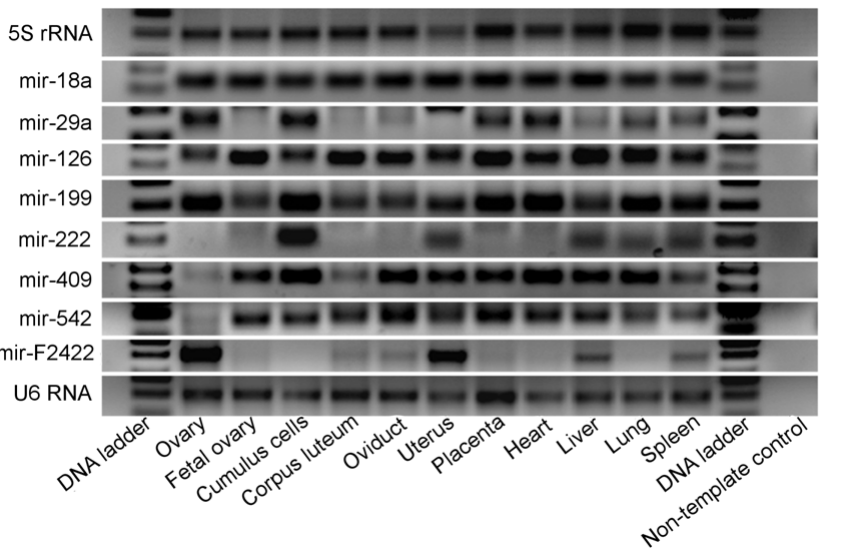
**Detection and expression analysis of selected miRNAs in multiple tissues**. Expression profiles of some representative miRNAs (out of detected 44 miRNAs) in multiple tissues by PCR approach. While the figures are presented in the additional file 2 and the expression for all are summarized in the table 2. Amplicons were analyzed on 2% agarose gel. 5S rRNAs and U6 RNA were used as a loading control. A DNA ladder (M) indicating the size of the fragments (50-100-150 nt) on each side. Ovary denotes only the ovarian cortex without corpus luteum.

The expression of all new miRNAs including nine annotated miRNAs (let-7b, mir-15b, mir-18a, mir-29a, mir-125b, mir-126, mir-145, mir-199a and mir-222) in 11 different bovine tissues were analyzed using semi-quantitative RT-PCR (details in Figure 4, Table 2 and Additional file 2). As small RNAs were cloned in the library derived from all compartments of the ovary, samples from ovarian cortex, cumulus cells and matured corpus luteum were used to determine the sub-cellular expression profile of the new miRNA using RT-PCR (Table 2). This is because of two facts: firstly, the bovine ovary is continuously changing throughout the process of folliculogenesis and secondly, the distinct nature of function of intra-ovarian cells and tissues compartments in the ovary.

**Table 2:** Detection and expression of selected miRNAs in multiple tissues

**miRNAs**	**Ovary^a^**	**Fetal Ovary^b^**	**Cumulus cells**	**Corpus luteum^c^**	**Oviduct**	**Uterus**	**Placenta**	**Heart**	**Liver**	**Lung**	**Spleen**
5s rRNA	+++	+++	+++	+++	+++	+++	+++	+++	+++	+++	+++
U6 RNA	+++	+++	+++	+++	+++	+++	+++	+++	+++	+++	+++
bta-let7b	++	+++	+++	+++	++	+	+++	++	+	++	+++
bta-mir-15b	+	+++	+++	+++	+++	+++	+++	+++	+++	+	+++
bta-mir-18a	+++	+++	+++	+++	+++	+++	+++	+++	+++	+++	+++
bomir-22*/22-5p	+	+	+	-	-	-	-	+	-	+	+
bta-mir-29a	+++	-	+++	-	-	-	+++	+++	+	++	++
bta-mir-125b	-	++	+++	++	++	+	++	++	-	+++	+
bta-mir-126	++	+++	++	+++	+++	+++	+++	+++	+++	+++	+++
bomir140*/140-5p	+++	+++	+++	++	++	++	++	++	++	-	++
bomir-143-3p	++	+++	+	++	+	+	+++	++	+	+	++
bta-mir-145	++	+++	-	++	-	-	+++	++	++	-	+++
bomir-152-5p	++	++	-	++	+++	+++	++	++	+++	+++	+++
bomir-193a-2-3p	+	++	++	-	++	++	++	+	+	++	++
Bta-mir-199	+++	++	+++	++	++	++	+++	+++	++	+++	+++
bta-mir-222	-	-	+++	-	-	++	-	-	+	+	+
bomir-378-3p	+++	+++	-	-	++	+++	+	++	+	+	-
bomir-382-3p	+	-	-	-	+++	+++	+++	+++	-	-	+++
bomir-409-5p	+	+++	+++	++	+++	+++	+++	+++	+++	+++	++
bomir-424-3p	++	-	-	-	+	++	-	-	++	-	+
bomir-503-3p	++	+++	+++	++	+	+	++	++	+	++	++
bomir-542-3p	-	++	++	++	+++	++	+++	++	++	+	+
bomir-574-5p	+	+	-	+	+	++	++	++	++	++	++
bomir-652-3p	+	+++	-	+++	++	+++	-	-	++	-	-
bomir-940-5p	+	-	-	-	+++	+	-	+	+++	++	-
bomir-F0132-5p	+++	+++	+++	+++	+++	+++	+++	+++	+++	+++	+++
bomir-F0244-5p	+	++	+++	++	+	++	++	+	++	+	+
bomir-H0222-3p	-	++	-	+++	+	+	++	++	-	+	+
bomir-A0321-3p	++	++	++	++	++	++	++	++	++	+	++
bomir-C0533-5p	+	+++	++	+	-	+	+	+	-	-	-
bomir-F0522-3p	-	-	++	++	-	-	-	+	-	-	-
bomir-F1351-3p	++	-	-	+++	++	+++	+	+++	+++	++	+++
bomir-F1353-5p	++	++	-	++	+	+++	+++	+	+++	-	+
bomir-D1431-5p	++	+++	++	+++	++	+	++	+++	-	+	-
bomir-C1511-5p	+	+	-	+	++	++	+	++	++	+	+
bomir-F1821-3p	++	++	++	++	++	++	++	++	++	++	++
bomir-C1931-5p	+	++	-	+++	+++	+++	+++	+++	+++	-	+++
bomir-A2143-3p	-	++	-	+++	+++	+++	++	++	++	++	+++
bomir-F2422-5p	+++	-	-	+	+	+++	-	-	++	-	+
bomir-F2531-3p	+	-	-	+	++	++	+	+++	++	+	-
bomir-G2511-3p	+	-	-	+	+	+++	+	+++	++	++	-
bomir-E2664-3p	++	++	++	++	+	++	++	+	++	+	++
bomir-D3011-3p	+	+	++	+	+	+	+	+	+	++	+
bomir-A3341-3p	+	+	+	+	+	+	+	+	+	+	+
bomir-A3711-5p	+	+	-	+	+	+	+++	+	+	+	+
bomir-A4052-5p	+	+	-	++	++	++	++	++	+++	++	++

Expression profiles of 44 miRNAs including all new miRNAs in multiple tissues by PCR approach. Amplicons were analyzed on 2% agarose gel. 5S rRNAs and U6 RNA were used as a loading control. Relative band intensity was categorized into four groups like '+++' for Strong signal, '++' for Medium signal, '+' for Weak signal or smear like product and '-' for not detected by comparing the expression signal of each miRNA to the expression level of 5S rRNA and U6 RNA. ^a^: Ovarian cortex with no visible corpus luteum, ^b^: Ovary from fetus at about 5^th ^month of pregnancy, ^c^: Mature corpus luteum from the same Ovary.

Of all 47 miRNAs (38 new and 9 already annotated miRNAs) 44 were detected in both ovarian cells and multiple tissues. Five miRNAs (bta-mir-126, bomir-F0132, bomir-A0321 and bomir-F1821) were found to be expressed at similar level in all experimental tissues. Seven miRNAs (bta-mir-18a, bta-mir-29a, bomir-140, bta-mir-199, bomir-378, bomir-F0132 and bomir-F2422) were found to be expressed at relatively higher levels in ovarian cortical portion (Table 2). On the other hand, all undetected or less expressed miRNAs in ovarian cortex were found to be highly expressed in cumulus cells or corpus luteum. Most of the miRNAs were found to be differentially expressed between adult ovarian tissues and fetal ovary. Among them bta-mir-15b, bomir-409, bomir-652, bomir-C0533 and bomir-D1431 were highly expressed in the fetal ovary compared to that of adult ovarian cortex. However, bta-mir-29a, bta-mir-199 and bomir-F2422 were found to be expressed at higher level in the adult ovarian cortex than that of the fetal ovary (Table 2). Bta-mir-125b, bta-mir-222, bomir-542, bomir-652, bomir-H0222, bomir-F0522, bomir-C1931 and bomir-A2143 were found to be expressed at very low level or not detected at all in the ovarian cortex. However, their abundance was higher in the cumulus cells and matured corpus luteum. The expression of bta-mir-222 was detected exclusively in the cumulus cells. In addition, higher expression of bta-mir-125b, bomir-409, bomir-503 and bomir-F0244 was also observed in the cumulus cells. The expression of bomir-652, bomir-H0222, bomir-C1931 and bomir-A2143 was higher in the corpus luteum.

Moreover, higher expression level of different miRNAs in various reproductive tissues was also observed. This includes bomir-940 in the oviduct; bta-mir-222, bomir-F2422 and bomir-G2511 in the uterus; and bta-mir-29a, bomir-143, bta-mir-145, bta-mir-199, bomir-542 in the placenta. All these investigated miRNAs were detected at least in one of the non-ovarian somatic tissues including heart, liver, lung and spleen (Table 2). The RT-PCR analysis did not confirm the expression of three novel miRNAs (bomir-F0131, bomir-H0121 and bomir-B0821) in any of the tissues under investigation (image not shown).

**Figure 5:**
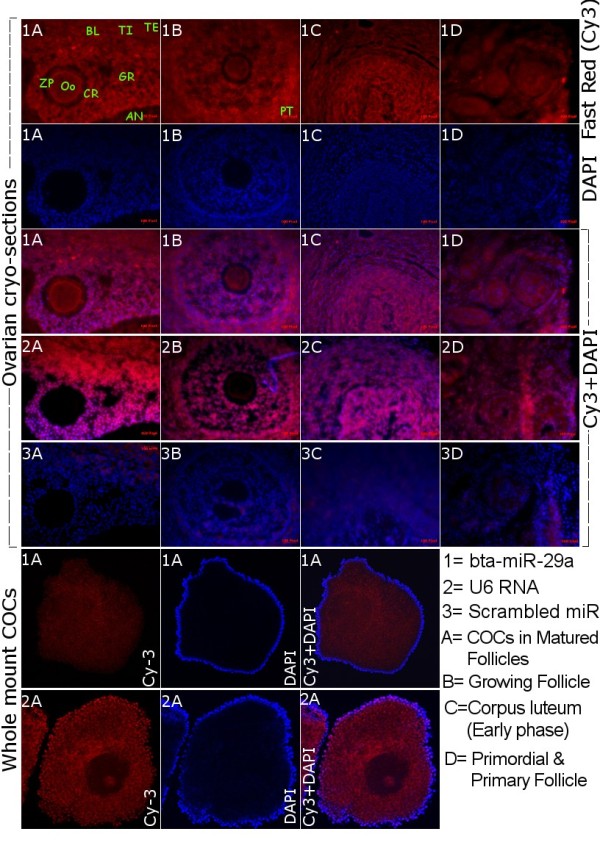
**In-situ detection of mir-29a in the ovarian sections and whole mount COCs**. Bovine ovarian cryo-sections and whole mount cumulus-oocyte complexes were in situ hybridized with 3'-digoxigenin labeled locked nucleic acid (LNA) microRNA probes for miR-29a (1), U6 RNA (2) and scrambled miRNA (3). BL- Basement Laminae, TI- Theca Interna, TE- Theca Externa, GR- Multiple layers of Granulosa, ZP-Zona Pellucida, OO-Oocyte, CR- Corona Radiata, AN-Antrum of the follicle, PT- Presumptive theca in the growing follicle.

In order to elucidate the cellular localization of one miRNA, bta-miR-29a was selected due to its differential expression between adult and fetal ovary, which are distinct in their functional activity. Accordingly, *in-situ *localization of this miRNA in the sections of bovine ovarian follicle revealed its expression in the different intra-ovarian cells (theca, mural granulosa, cumulus granulosa and oocyte) of different stages of development including primordial, primary, growing and matured/tertiary follicles (Figure 5). Stable expression was detected in the whole mount cumulus-oocyte-complexes derived from the follicles of more than 8 mm in diameter. In the semi-quantitative RT-PCR data, expression of this miRNA was found in the cortex region of the adult ovary where follicles with cumulus cells are residing. Moreover, the expression of this miRNA was detected further until early stage of corpus luteum (Figure 5), but very low or no expression in the matured corpus luteum (Figure 4).

### Prediction and functional categorization of cloned miRNA targets

**Figure 6:**
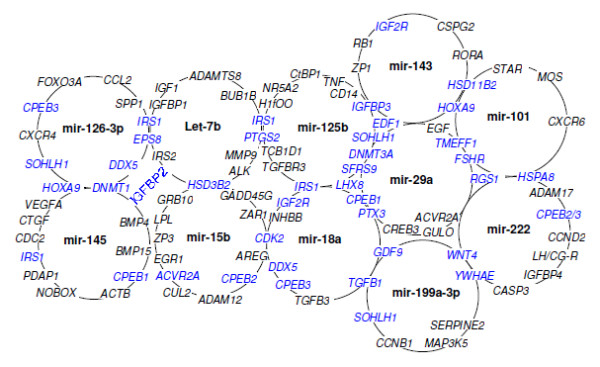
**Eleven miRNAs with highest number of screened target genes (sub-set miRNAs targets)**. Each circle representing one miRNAs and the surrounding genes are targeted by that miRNA. Genes shared by the different circles highlighted as blue (overlapping genes between miRNAs), which are commonly targeted by the corresponding miRNAs.

The goal of this prediction and analysis was to find the major biological processes and signaling pathways in the ovary that are most likely affected by a group of miRNAs. Even though there were many potential target genes predicted for the cloned miRNAs, several filtering and screening procedures (see materials and methods) have enabled us to generate a comprehensive target list consisting of 115 potential genes from all the predicted targets (Additional file 3). From this screened target set, we found that let-7b, mir-15b, mir-18a, mir-29a, mir-101, mir-125b, mir-126, mir-143, mir-145, mir-199a and mir-222 to have the highest number and overlapping targets (Figure 6). Interestingly, we found that all of these targeting miRNAs were represented at higher frequency in our constructed library.

**Figure 7:**
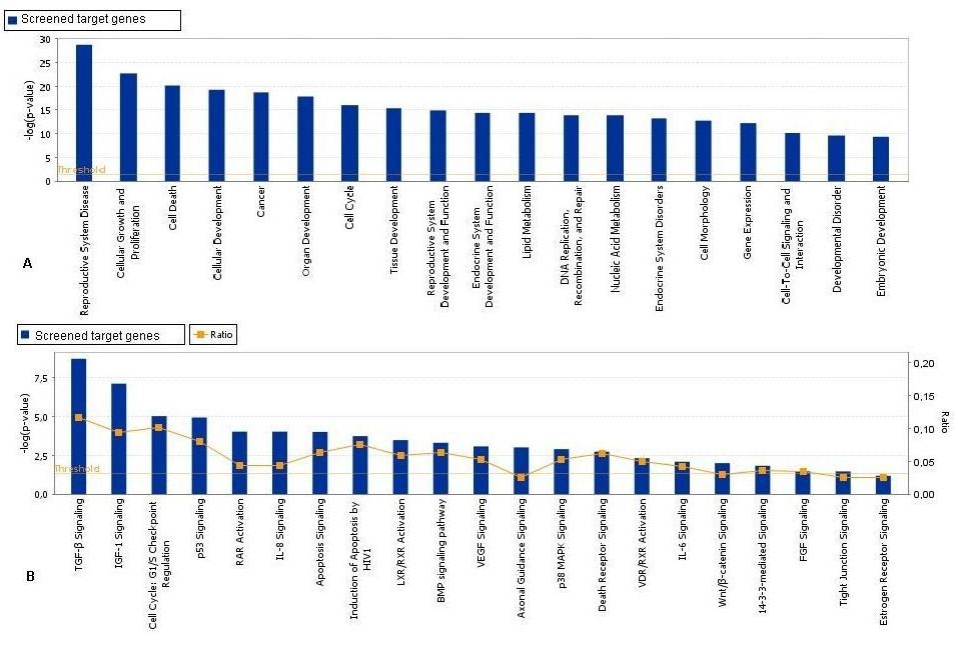
**Top biological function, disease categories and pathways enriched with predicted and screened miRNA target genes**. A. Top biological functions and disease categories and B. pathways enriched with predicted and screened miRNA target genes. Ratio is the number of affected genes to total number of genes in the pathway. Threshold p < 0.05 is shown as yellow line. Bars that are above the line indicate significant enrichment of a functional category or pathway.

Detailed Gene Ontology (GO) analysis of the screened and sub-sets of miRNAs target genes were found to be associated with reproductive system development, function and disorders. These include cell cycle, morphology, cell death, cell to cell signaling, cellular growth, development and proliferation, DNA replication, recombination & repair, endocrine system disorder and different pathways underlying the ovarian functions. To further elucidate the specific functions of these genes, a detailed pathway analysis was performed using Ingenuity Pathway Analysis (Redwood City, California) for all target sets (Figure 7) as well as for the sub-set of genes (Table 3, Additional file 3).

**Table 3:** Ingenuity analysis of the genes targeted by top eleven screened miRNAs

**miRNAs**	**Functions and disease categories enriched with the selected miRNA targets**	**Canonical Pathways enriched with the selected miRNA targets**
Let-7b	Tissue morphology, cellular growth and proliferation, endocrine system disorders	IGF-1 signaling, hepatic fibrosis/hepatic stellate cell activation
mir-15b	cell death, connective tissue development and function, cell cycle	p53 signaling, PPARα/RXR activation
mir-18a	Cell cycles, cellular function, endocrine system development	Cell cycle: G1/S checkpoint regulation, TGF-β signaling
mir-29a	Reproductive system development and function, organ development, endocrine system development	Ephrin receptor signaling Aminophosphonate metabolism
mir-101	Endocrine system development, lipid metabolism, small molecule biochemistry	C21-steroid hormone metabolism, Androgen and estrogen metabolism
mir-125b	Inflammatory response, cell cycle, cellular function and maintenance	LPS/IL-1 Mediated inhibition of RXR function, LXR/RXR activation
mir-126	Cellular movement, Endocrine system disorders, cell mediated immune response	Pro-apoptosis, PXR/PXR activation
mir-143	Cellular growth and proliferation, DNA replication, recombination and repair, gene expression	G1/S transition of the cell cycle, p53 signaling
mir-145	Reproductive system diseases, reproductive system development and function, cell death	BMP signaling pathway, VEGF signaling
mir-199a	Cellular development, cell death, cellular growth and proliferation	Cell cycle: G2/M DNA damage checkpoint regulation, p38 MAPK signaling
mir-222	Cellular development, reproductive system development and function, cell death	IGF-1 signaling, Axonal guidance signaling

Eleven top miRNAs targeting highest number of genes from our screened and filtered all predicted targets and their top Gene Ontology categories and pathways based on Fisher' Exact P-value (< 0.05) are presented in the table. For detail figures see additional file 3.

## Discussion

### Identification of small RNAs

MicroRNAs play an integral part of animal gene regulatory networks as one of the most abundant classes of gene regulators. They are estimated to comprise 1-5% of animal genes [8,36,37] or a given genome could encode nearly thousands of miRNAs [36]. Moreover, a typical miRNA regulates hundreds of target genes [38-41] and altogether they could target a large proportion of genes up to 30% of the genome [42]. Changes in the expression of even a single miRNA could have a significant impact on the outcome of diverse cellular activities regulated by the product of those genes. Beyond the strict conservation of miRNAs across different species, some miRNAs appear to be species specific [32,36,43]. Compared with computational or heterologus approaches, direct cloning has the advantage of identifying non-conserved and new miRNAs.

Our cloning and expression analysis led to the identification of 74 miRNAs out of which 38 are new in bovine. Mature sequences were found to be conserved in closely related species, but when considering precursor sequence only 51% was found to be conserved in human, mouse, rat, dog, horse and also in other non-mammalian vertebrates. However, in the present study, 17 miRNA precursors corresponding to 21 genomic loci were found to be not conserved (Table 1). This could be either due to the lack of sequences in draft genome assembly or these miRNAs are bovine specific. The genomic properties of our new miRNAs showed that they are derived from exon, intron and intergenic region. This may suggest that these miRNAs can be transcribed in parallel with their host transcripts. In addition, two different transcription classes of miRNAs ('exonic' and 'intronic') recognized here may require somewhat different mechanisms of bio-genesis as stated previously [44]. Discovery of six clusters composed of 15 miRNA genes on six chromosomes showed that these closely located host genes may share the same cis-regulatory elements and the miRNAs within the clusters might be expressed in the same tissues or at the same developmental or physiological stage.

The representation of many known and novel miRNAs in this single library indicates the presence of potential miRNAs, which are not yet discovered. Both in-depth analysis of the existing library and cell-type-specific analysis of individual miRNAs will give insight into the functional mechanisms and pathways involved in ovarian folliculogenesis in particular and female fertility in general.

Along with the miRNAs several types of endogenous small interfering RNAs were identified in the present study. Among them, 27 distinct rasiRNAs represented the frequent class of small RNAs. Thirteen RNAs were classified as small antisense RNAs, while 17 small RNAs were tiny non-coding RNAs. The small RNA cloning and profiling from another study revealed less representation of that group of rasiRNAs compared to the miRNAs [33]. The properties we identified for rasiRNAs support the notion that they are presumably emerged from dsRNA produced by annealing of sense and antisense transcripts that contain repeat sequences related to transposable elements [27]. These rasiRNAs are known to repress the repeat sequences at the transcriptional or post-transcriptional level and maintain a centromeric heterochromatic structure [45]. Identity and properties of new types of small RNAs in the present study showed the presence of diverse modes of small RNA-mediated gene regulation in bovine ovary, as reported in other species [26]. Therefore, identification and characterization of other small RNAs and their expression patterns are important for elucidating detailed gene regulatory networks involved in the ovary. So, all these endogenous small interfering RNAs need to be further characterized to elucidate their cellular functions.

### Expression of miRNAs in diverse tissue types

Expression analysis of 44 miRNAs in different ovarian cells and tissues types has enabled us to determine their site of action in terms of tissue specific abundance as well as functional regulation (Table 2). We have detected nearly all of these miRNAs in at least one part of the entire ovary and other somatic tissues. In the present study, some miRNAs appear to be extremely tissue specific. For example; bomir-C0533 and bomir-F0522 were found to be exclusively expressed in ovarian tissues suggesting their potential role in ovary-specific miRNA-dependent regulatory processes. Five miRNAs (miR-29a, miR-125b, bomir-409, bomir-503 and bomir-F0244) were found to be highly abundant in the cumulus cells and four (bomir-652, bomir-H0222, bomir-C1931 and bomir-A2143) in corpus luteum. These cumulus enriched miRNAs in the present study may represent those miRNAs with potential association with the regulation of cumulus secreted factors, which are important for cumulus-oocyte communication and subsequent oocyte development. Similar study in mouse showed hormonal regulation of miRNAs expression in preovulatory mural granulosa cell [25].

Altered expression of various ovary related genes was reported in ovaries from fetal, new born and adult animals [46-49]. Furthermore, alteration in expression of small RNAs has been addressed at different stages of mouse ovary [24]. Similarly, in the present study we found differential expression of mir-29a, bomir-140, mir-199, mir-378, bomir-F0132 and bomir-F2422 in the ovarian cortical portion between fetal and adult cows. This may indicate their possible involvement in regulating follicular development in the adult cyclic ovarian function. This notion was further supported by higher detection of miR-29a in different follicular cells (theca, cumulus-granulosa, and oocyte) of adult ovary by in situ hybridization (Figure 5) and higher expression in cumulus cells by RT-PCR but no detection in the fetal ovary. The expression of miRNAs in ovarian cells is reported to be regulated by FSH and LH/hCG [25,50] which functions in the cyclic ovary but not in fetal ovary [51]. Moreover, most of the targets predicted for this miRNA (Figure 6) are known to be involved in various cyclic adult ovarian functions.

Noticeable expression level of miR-29a was found in different phases of corpus luteum (CL) development. According to RT-PCR and in situ hybridization results, expression of miR-29a was detected in the early phase CL but not in mid phase (matured) CL. These two phases of CL development are known to vary in multiple aspects of luteal physiology, angiogenesis and sensitivity to luteolytic actions, which are accompanied by differential expression of multiple genes [52-55]. Bovine corpus luteum is reported to be resistant to luteolysis by exogenous PGF2α in early stage of CL (before Day 5) due to differential expression of genes associated with the PGF2α receptor [55]. Considering these facts and restricted expression of miR-29a in early phase of CL in the present study, it is possible to suggest that miR-29a is involved in gene regulatory action during early phase of CL. All in all, our results on miR-29a may elucidate the potential involvement of this regulatory miRNA in growth and differentiation of cumulus cells, endocrine regulation of theca cells and early luteinisation in cyclic ovary.

Cloning, determining potential secondary structures and expression analysis of all new miRNAs in multiple tissues indicate their tissue specific existence and regulation of gene expression. Only 7.8% of the new miRNAs could not be detected by the RT-PCR procedure in various reproductive tissues. This may be due to the fact that these transcripts were cloned at lower frequency (only once) showing their lower abundance and subsequent difficulty to detect them [30]. In general, the expression profiling analysis in the present study revealed that our cloned miRNAs were either ubiquitously expressed in multiple tissues or preferentially expressed in a few tissues including the intra-ovarian cells and tissues.

### Features of predicted target genes

Multiple genes contributing to mammalian folliculogenesis have been identified in mouse knockout study [56]. Primarily, oocyte-specific transcriptional regulators such as *Figla, Nobox*, *Sohlh1 *and *Lhx8*, oocyte-secreted factors such as *Gdf9 *and *Bmp15*, as well as genes expressed in the granulosa and cumulus cells (*FSHR *and *PTX3*) were found to initiate and control follicular growth [56,57]. Among the key intra-ovarian factors, the transforming growth factor β (*TGFβ*) family members, of which *bone morphogenetic protein-4 *have been identified as regulators of primordial germ cell generation [58].

In response to FSH, the granulosa cell-derived factors such as kit ligand, transforming growth factor *alfa *(*TGF-α*) and epidermal growth factor (*EGF*) activate the resting follicular growth. The interactions between ovarian germ and somatic cells (granulosa cells and the oocytes) and expression of several intra-ovarian autocrine/paracrine regulators (*FSH, estrogen and androgen receptors*) are the major contributing factors in the ovary leading to preantral and antral follicles development [59].

During follicle growth, *IGF *system works in synergy with gonadotrophins (follicle-stimulating hormone and luteinising hormone) to regulate proliferation and differentiation of granulosa and theca cells [60,61]. In addition, it has been shown that the processes of follicular dynamics (Recruitment, selection, dominance and ovulation) are associated with temporal changes of peripheral gonadotropins concentration and *IGF *system [62-68]. All the above-mentioned genes are represented in our predicted and analyzed targets. Altogether 115 genes were among potential target genes of our identified miRNA. These target genes are already experimentally validated for potential ovary related functions in different mammalian species (references in additional file 3). Interestingly, several well-known target genes including *IRS1, IGFBP3, DNMT3A, HOXA9, TNF*, etc. which are identified by our new screening approach, were already validated in wet lab experiments and reported as targets of multiple miRNAs (miR-145, miR-125b, miR-126 and miR-29) [69-73]. Accordingly these studies have elucidated the potential involvement of these miRNAs in broad class of functions related to apoptosis, differentiation signal, cell differentiation, tumorogenesis, DNA methylation and innate immune responses.

Gonadotropins, intra-ovarian mediators and their receptors which are identified as target genes for our miRNAs might mediate important intracellular actions necessary for normal follicular development and other ovarian functions. Alterations in the expression of these mediators by miRNAs will result in various ovarian dysfunctions causing infertility, polycystic ovary syndrome and tumorigenesis. Recent evidences also support our hypothesis, where at least six of our 11 top ranked ovarian miRNAs were found to be related to cancer or tumors in the ovary. For example, miR-199a, miR-145, miR-125b and let-7 clusters were found to be the most differentially regulated miRNAs in human ovarian cancer [74,75]. While miR-145 [76] and mir-199a [77] have recently been shown to be down-modulated in the tumor cells, the miR-222 is reported to be down-regulated in ovarian epithelial carcinomas [74]. Furthermore, higher expression of miR-18a and lower expression of let-7b and miR-199a were shown to be correlated with serous ovarian carcinoma [78]. In another study, miR17-5p and let-7b were found to be involved in the regulation of development and function of the ovarian corpus luteum specially angiogenesis of corpus luteum [23]. Interestingly, nearly all of these 11 selected miRNAs (Figure 6) in the present study are reported to be differentially regulated in endometrium of women with and without endometriosis [79]. Taken together, our findings and other evidences support the relevance of these 11 miRNAs to ovarian physiology and may be the most important regulatory miRNA group in ovary, as their predicted and analyzed target genes are involved in a broad range of signaling cascades and pathways of the ovarian function.

## Conclusion

The presence of distinct miRNAs and other small RNAs, with different expression patterns and various target genes in bovine ovary revealed the potential role of such miRNAs in follicular development in particular and female fertility in general. Further functional characterization of some selected miRNAs including expression profiling and in situ localization in the ovarian follicles at different cyclic stages will supplement the results of this study and help to elucidate their specific roles in the ovarian function. The information we generated from this study will help to identify candidate miRNAs targeting specific molecular and cellular pathways important for ovarian follicular development, atresia, ovulation as well as ovarian dysfunction.

## Methods

### Isolation of Small RNAs and subsequent miRNAs fractionation

Bovine ovaries were obtained from a cyclic heifer with the age of 30 months at a stage of mid cycle with a visible mature corpus luteum including normally distributed different types of follicles. Small RNA samples from various bovine tissues and cells were isolated using mirVana miRNA isolation kit (Applied Biosystems Inc, Foster City, CA) according to the manufacturer's instructions. For cloning, 10 μg of the ovarian small RNA was loaded into 12% denaturing poly acrylamide gel electrophoresis with size markers miSPIKE (Integrated DNA Technologies, Inc., Iowa, USA) and fractions of 18-26 nt were recovered using DTR gel filtration cartridge (Edge BioSystems, Maryland, USA).

### Cloning of small RNAs

For cloning the small RNAs, we followed "5' Ligation independent Cloning" to ensure complete recovery of conventional small RNAs as well as small RNAs with 5' modifications or non-standard 5' ends. All the linkers and primers were obtained from Integrated DNA Technologies, Inc., Iowa, USA. List and sequence of linkers and primers are given in additional file 4. Briefly, once the enriched small RNA fraction was recovered from the acrylamide gel slice, the small RNAs were ligated with a 3' linker - adenylated oligos, modified with a 3'-terminal dideoxy-C (ddC) containing Ban-I restriction site [5]. The ligated products were loaded on dPAGE for purification and reverse transcription was performed. An exonuclease digestion was carried out after first strand cDNA synthesis and then a second 3' ligation was carried out using a different linker sequences. The second 3' linkered product (60 nt) was purified from dPAGE to remove free linkers. Subsequently, the amplification of the RT-PCR product was performed using linker specific primer set with the thermocycler program of 95.0°C for 10 minutes, 35 cycles of (95.0°C for 30 seconds, 52.0°C for 30 seconds 72.0°C for 30 seconds) and followed by incubation at 72.0°C for 5 minutes. Then, the amplicon was subjected to Ban I digestion, concatemerization and end filling with non-template adenosine followed by cloning into TOPO TA Cloning^® ^vector (Invitrogen, Carlsbad, CA). Concatemer clones were picked up, cultured and then colony PCR was performed for screening the insert size. Plasmid DNA preparation and DNA sequencing were performed for screened clones and small RNAs which were separated by well defined linker units with the reconstituted *Ban I *site.

### Bioinformatic analysis of small RNA sequences

The small RNA sequences were first compared with the sequences in miRBase [32,80-82]. Small RNAs completely or partially matched by less than two mismatches to any registered miRNA in miRBase were considered putative bovine miRNA. The remaining sequences were compared to the bovine nucleotide collection (nr/nt) and the expressed sequence tags (EST) database in NCBI [83] and different noncoding RNA databases [84-88]. Sequences, which were matched 100% to any mRNA, rRNA or tRNA were excluded from further evaluation to generate novel miRNA candidates. All the remaining sequences and the putative bovine miRNA sequences were submitted to BLAST-search in the Ensembl 52: bovine genome assembly (Btau_4.0) [35] and the 75 bp genomic flanking sequence upstream from the 3' end or downstream from the 5' end of the miRNA was considered putative precursor of the matching miRNA.

All the putative precursor sequences were analyzed for hairpin structure using the mfold Web server (version 3.2) [89] to evaluate the ability to form thermodynamically stable hairpin structures [90] based on other criteria described elsewhere [32]. Chromosome locations, orientation and genomic features of the predicted miRNA precursors as well as other small RNAs sequences (not meeting miRNAs criteria) and whether they were located in intragenic or intergenic genomic regions were determined using ensembl. Other small RNAs were categorized according to published research articles [26,27,33].

### Detection of miRNAs expression by semi-quantitative RT-PCR

Small RNA samples isolated from the 11 different tissues and cells, such as ovarian cortex, fetal ovary at about six month of pregnancy, cumulus cells, matured corpus luteum, oviduct (entire), uterus (horn), placenta, heart, liver, lung and spleen were used for the detection of miRNAs by PCR method according to Ro *et al *[91] with some modifications. Briefly, the poly (A)-tailed small RNA was purified by acid phenol: chloroform: iso-amyl alcohol and ethanol precipitation method. All small RNA-cDNA samples were diluted to the same concentration of 6 ng/μl (which was the lowest amount obtained from cumulus cells). Three microliters of cDNA was used as template for conventional PCR and the products were analyzed on a 2% agarose gel. List of primers and oligos used are shown in the additional file 4. Some representative RT-PCR products were cloned into PGEM-T easy vector (Promega Corporation, Wisconsin, USA) and transformed to *E. coli *and sequenced to verify the specificity of PCR amplification.

### In situ hybridization of miRNAs in ovarian cryo-sections and whole mount COCs

For in-situ hybridization of miRNAs, bovine ovary (21/0) days of estrus cycle was fixed in 4% PFA overnight at 4°C followed by overnight incubation in PBS with 30% sucrose at 4°C and frozen in Tissue-Tek OCT reagent (Sakura Finetek, Zoeterwoude, NL). Cryo-sections (10 μm) preparation, post-fixation, acetylation and proteinase K treatment were carried out as described previously [92]. Two hours of pre-hybridization was performed at 52°C in hybridization solution (50% formamide, 5× sodium chloride/sodium citrate [SSC; pH 6.0], 0.1% Tween-20, 50 μg/ml heparin, and 500 mg/ml yeast tRNA). Ovarian sections were incubated overnight at 52°C with 3'-Digoxigenin (DIG) labeled LNA-modified oligonucleotide probes (1 pM) for miR-29a, U6 RNA and scrambled miR (Exiqon, Vedbaek, Denmark) in hybridization buffer in a humidified chamber. Blocking, incubation with anti-DIG-AP antibody, washing and color development using Fast Red reaction was performed as described previously [92]. The slides were mounted with VectaShield containing DAPI (Vector laboratories, Burlingame, CA) and analyzed by confocal laser scanning microscope (CLSM LSM-510, Carl Zeiss, Germany). For whole mount in-situ hybridization, cumulus oocyte complexes were aspirated from more than 8 mm of ovarian follicles. Pre-fixation, processing, digestion with Proteinase K, pre-hybridization, hybridization, post-hybridization washing was performed in 4-well embryo culture dishes according to the high-resolution whole mount in situ hybridization protocol from Exiqon. The rest of the procedures were similar to cryo-section hybridization protocol.

### Prediction and analysis of ovarian miRNA targets

For this purpose, initially a raw list of all genes found to be targeted by our cloned miRNA was generated using MIRANDA algorithm, miRBase target version 5 [93]. Subsequently, about 800 distinct important genes related to mammalian reproductive system development, function and disorders were extracted from Ingenuity knowledge base (IPA 7.0) by key word search. Then, we applied two filtration steps to generate a comprehensive list of target genes. Firstly, raw target set and genes set extracted from database were cross-matched and common genes were extracted. Secondly, we applied the condition that multiple genes targeted by multiple miRNAs from the common target list. From these screened target sets, 11 miRNAs having the highest number as well as overlapping target genes were enlisted. Then, the Gene Ontology (GO) analysis of the screened and sub sets of miRNAs target genes were performed in order to predict the possible biological processes and functions that were most likely to be affected by miRNAs using web delivered tools of Ingenuity Pathway Analysis (Redwood City, California). Top significant GO categories, biological functions and different canonical pathways were analyzed for miRNA specific targets as well as for all screened targets based on significant over-representation of genes using a selected threshold for p-values ≤ 0.05 of hypergeometric distribution [94].

## Note

We have submitted our newly identified miRNAs into the miR-base and they are annotated accordingly.

## Authors' contributions

MMH was responsible for miRNAs cloning, detection and drafting the manuscript. NG analyzed predicted target genes and reviewed the manuscript. MH supplied reproductive samples. CP, ET in coordination of bioinformatic analysis. KS contributed by supervising the work with necessary suggestion. DT was responsible for project development, reviews the manuscript and is the corresponding author. All contributing authors reviewed and approved before submitting the final copy of this manuscript.

## Supplementary Material

Additional file 1 **List and Bio-informatic analysis of the sequences.** Click here for file

Additional file 2 **Predicted secondary structure of new miRNAs and detection of expression in multiple tissues.** Click here for file

Additional file 3 **Screened target genes list for cloned miRNAs and figures of GO analysis.** Click here for file

Additional file 4 **List of oligos and primers used for this study.** Click here for file

## References

[B1] Hunter MG, Robinson RS, Mann GE, Webb R (2004). Endocrine and paracrine control of follicular development and ovulation rate in farm species. Animal Reproduction Science.

[B2] Bonnet A, Dalbies-Tran R, Sirard MA (2008). Opportunities and challenges in applying genomics to the study of oogenesis and folliculogenesis in farm animals. Reproduction.

[B3] Eichenlaub-Ritter U, Peschke M (2002). Expression in in-vivo and in-vitro growing and maturing oocytes: focus on regulation of expression at the translational level. Hum Reprod Update.

[B4] Piccioni F, Zappavigna V, Verrotti AC (2005). Translational regulation during oogenesis and early development: the cap-poly(A) tail relationship. C R Biol.

[B5] Lau NC, Lim LP, Weinstein EG, Bartel DP (2001). An abundant class of tiny RNAs with probable regulatory roles in Caenorhabditis elegans. Science.

[B6] Alvarez-Garcia I, Miska EA (2005). MicroRNA functions in animal development and human disease. Development.

[B7] Ambros V (2004). The functions of animal microRNAs. Nature.

[B8] Bartel DP (2004). MicroRNAs: genomics, biogenesis, mechanism, and function. Cell.

[B9] Chen K, Rajewsky N (2007). The evolution of gene regulation by transcription factors and microRNAs. Nat Rev Genet.

[B10] Lai EC (2003). microRNAs: Runts of the Genome Assert Themselves. Current Biology.

[B11] Plasterk RH (2006). Micro RNAs in animal development. Cell.

[B12] Lee Y, Jeon K, Lee JT, Kim S, Kim VN (2002). MicroRNA maturation: stepwise processing and subcellular localization. EMBO J.

[B13] Yi R, Qin Y, Macara IG, Cullen BR (2003). Exportin-5 mediates the nuclear export of pre-microRNAs and short hairpin RNAs. Genes Dev.

[B14] Lee Y, Ahn C, Han J, Choi H, Kim J, Yim J, Lee J, Provost P, Radmark O, Kim S (2003). The nuclear RNase III Drosha initiates microRNA processing. Nature.

[B15] Pillai RS, Bhattacharyya SN, Artus CG, Zoller T, Cougot N, Basyuk E, Bertrand E, Filipowicz W (2005). Inhibition of translational initiation by Let-7 MicroRNA in human cells. Science.

[B16] Liu J, Rivas FV, Wohlschlegel J, Yates JR, Parker R, Hannon GJ (2005). A role for the P-body component GW182 in microRNA function. Nat Cell Biol.

[B17] Liu J, Valencia-Sanchez MA, Hannon GJ, Parker R (2005). MicroRNA-dependent localization of targeted mRNAs to mammalian P-bodies. Nat Cell Biol.

[B18] Rehwinkel J, Behm-Ansmant I, Gatfield D, Izaurralde E (2005). A crucial role for GW182 and the DCP1:DCP2 decapping complex in miRNA-mediated gene silencing. RNA.

[B19] Bernstein E, Kim SY, Carmell MA, Murchison EP, Alcorn H, Li MZ, Mills AA, Elledge SJ, Anderson KV, Hannon GJ (2003). Dicer is essential for mouse development. Nat Genet.

[B20] Giraldez AJ, Cinalli RM, Glasner ME, Enright AJ, Thomson JM, Baskerville S, Hammond SM, Bartel DP, Schier AF (2005). MicroRNAs regulate brain morphogenesis in zebrafish. Science.

[B21] Wienholds E, Koudijs MJ, van Eeden FJ, Cuppen E, Plasterk RH (2003). The microRNA-producing enzyme Dicer1 is essential for zebrafish development. Nat Genet.

[B22] Otsuka M, Jing Q, Georgel P, New L, Chen J, Mols J, Kang Young J, Jiang Z, Du X, Cook R (2007). Hypersusceptibility to Vesicular Stomatitis Virus Infection in Dicer1-Deficient Mice Is Due to Impaired miR24 and miR93 Expression. Immunity.

[B23] Otsuka M, Zheng M, Hayashi M, Lee JD, Yoshino O, Lin S, Han J (2008). Impaired microRNA processing causes corpus luteum insufficiency and infertility in mice. J Clin Invest.

[B24] Ro S, Song R, Park C, Zheng H, Sanders KM, Yan W (2007). Cloning and expression profiling of small RNAs expressed in the mouse ovary. RNA.

[B25] Fiedler SD, Carletti MZ, Hong X, Christenson LK (2008). Hormonal regulation of MicroRNA expression in periovulatory mouse mural granulosa cells. Biol Reprod.

[B26] Ambros V, Lee RC, Lavanway A, Williams PT, Jewell D (2003). MicroRNAs and other tiny endogenous RNAs in C. elegans. Curr Biol.

[B27] Aravin AA, Lagos-Quintana M, Yalcin A, Zavolan M, Marks D, Snyder B, Gaasterland T, Meyer J, Tuschl T (2003). The small RNA profile during Drosophila melanogaster development. Dev Cell.

[B28] Reinhart BJ, Weinstein EG, Rhoades MW, Bartel B, Bartel DP (2002). MicroRNAs in plants. Genes Dev.

[B29] Coutinho LL, Matukumalli LK, Sonstegard TS, Van Tassell CP, Gasbarre LC, Capuco AV, Smith TP (2007). Discovery and profiling of bovine microRNAs from immune-related and embryonic tissues. Physiol Genomics.

[B30] Gu Z, Eleswarapu S, Jiang H (2007). Identification and characterization of microRNAs from the bovine adipose tissue and mammary gland. FEBS Lett.

[B31] Tesfaye D, Worku D, Rings F, Phatsara C, Tholen E, Schellander K, Hoelker M (2009). Identification and expression profiling of microRNAs during bovine oocyte maturation using heterologous approach. Mol Reprod Dev.

[B32] Ambros V, Bartel B, Bartel DP, Burge CB, Carrington JC, Chen X, Dreyfuss G, Eddy SR, Griffiths-Jones S, Marshall M (2003). A uniform system for microRNA annotation. RNA.

[B33] Aravin A, Tuschl T (2005). Identification and characterization of small RNAs involved in RNA silencing. FEBS Letters.

[B34] Webb R, Gong JG, Law AS, Rusbridge SM (1992). Control of ovarian function in cattle. J Reprod Fertil Suppl.

[B35] Ensembl 52: B. taurus. http://www.ensembl.org/Bos_taurus/Info/Index.

[B36] Bentwich I, Avniel A, Karov Y, Aharonov R, Gilad S, Barad O, Barzilai A, Einat P, Einav U, Meiri E (2005). Identification of hundreds of conserved and nonconserved human microRNAs. Nat Genet.

[B37] Berezikov E, Guryev V, Belt J van de, Wienholds E, Plasterk RH, Cuppen E (2005). Phylogenetic shadowing and computational identification of human microRNA genes. Cell.

[B38] Brennecke J, Stark A, Russell RB, Cohen SM (2005). Principles of microRNA-target recognition. PLoS Biol.

[B39] Krek A, Grun D, Poy MN, Wolf R, Rosenberg L, Epstein EJ, MacMenamin P, da Piedade I, Gunsalus KC, Stoffel M (2005). Combinatorial microRNA target predictions. Nat Genet.

[B40] Lewis BP, Burge CB, Bartel DP (2005). Conserved seed pairing, often flanked by adenosines, indicates that thousands of human genes are microRNA targets. Cell.

[B41] Xie X, Lu J, Kulbokas EJ, Golub TR, Mootha V, Lindblad-Toh K, Lander ES, Kellis M (2005). Systematic discovery of regulatory motifs in human promoters and 3' UTRs by comparison of several mammals. Nature.

[B42] Lim LP, Lau NC, Garrett-Engele P, Grimson A, Schelter JM, Castle J, Bartel DP, Linsley PS, Johnson JM (2005). Microarray analysis shows that some microRNAs downregulate large numbers of target mRNAs. Nature.

[B43] Stark A, Lin MF, Kheradpour P, Pedersen JS, Parts L, Carlson JW, Crosby MA, Rasmussen MD, Roy S, Deoras AN (2007). Discovery of functional elements in 12 Drosophila genomes using evolutionary signatures. Nature.

[B44] Rodriguez A, Griffiths-Jones S, Ashurst JL, Bradley A (2004). Identification of mammalian microRNA host genes and transcription units. Genome Res.

[B45] Lippman Z, Martienssen R (2004). The role of RNA interference in heterochromatic silencing. Nature.

[B46] Herrera L, Ottolenghi C, Garcia-Ortiz JE, Pellegrini M, Manini F, Ko MS, Nagaraja R, Forabosco A, Schlessinger D (2005). Mouse ovary developmental RNA and protein markers from gene expression profiling. Dev Biol.

[B47] Baillet A, Mandon-Pepin B, Cabau C, Poumerol E, Pailhoux E, Cotinot C (2008). Identification of transcripts involved in meiosis and follicle formation during ovine ovary development. BMC Genomics.

[B48] Vaskivuo TE, Anttonen M, Herva R, Billig H, Dorland M, te Velde ER, Stenback F, Heikinheimo M, Tapanainen JS (2001). Survival of human ovarian follicles from fetal to adult life: apoptosis, apoptosis-related proteins, and transcription factor GATA-4. J Clin Endocrinol Metab.

[B49] Olesen C, Nyeng P, Kalisz M, Jensen TH, Moller M, Tommerup N, Byskov AG (2007). Global gene expression analysis in fetal mouse ovaries with and without meiosis and comparison of selected genes with meiosis in the testis. Cell Tissue Res.

[B50] Yao N, Lu CL, Zhao JJ, Xia HF, Sun DG, Shi XQ, Wang C, Li D, Cui Y, Ma X (2009). A network of miRNAs expressed in the ovary are regulated by FSH. Front Biosci.

[B51] Abel MH, Wootton AN, Wilkins V, Huhtaniemi I, Knight PG, Charlton HM (2000). The effect of a null mutation in the follicle-stimulating hormone receptor gene on mouse reproduction. Endocrinology.

[B52] Wiltbank MC, Shiao TF, Bergfelt DR, Ginther OJ (1995). Prostaglandin F2 alpha receptors in the early bovine corpus luteum. Biol Reprod.

[B53] Copelin JP, Smith MF, Garverick HA, Youngquist RS, McVey WR, Inskeep EK (1988). Responsiveness of bovine corpora lutea to prostaglandin F2 alpha: comparison of corpora lutea anticipated to have short or normal lifespans. J Anim Sci.

[B54] Watts TL, Fuquay JW (1985). Response and fertility of dairy heifers following injection with prostaglandin F(2alpha) during early, middle or late diestrus. Theriogenology.

[B55] Goravanahally MP, Salem M, Yao J, Inskeep EK, Flores JA (2009). Differential gene expression in the bovine corpus luteum during transition from early phase to midphase and its potential role in acquisition of luteolytic sensitivity to prostaglandin F2 alpha. Biol Reprod.

[B56] Matzuk MM, Lamb DJ (2002). Genetic dissection of mammalian fertility pathways. Nat Cell Biol.

[B57] Dumesic DA, Abbott DH (2008). Implications of polycystic ovary syndrome on oocyte development. Semin Reprod Med.

[B58] Hurk R van den, Zhao J (2005). Formation of mammalian oocytes and their growth, differentiation and maturation within ovarian follicles. Theriogenology.

[B59] Filicori M, Cognigni GE, Pocognoli P, Ciampaglia W, Bernardi S (2003). Current concepts and novel applications of LH activity in ovarian stimulation. Trends Endocrinol Metab.

[B60] Campbell BK, Baird DT, Webb R (1998). Effects of dose of LH on androgen production and luteinization of ovine theca cells cultured in a serum-free system. J Reprod Fertil.

[B61] Gutierrez CG, Campbell BK, Webb R (1997). Development of a long-term bovine granulosa cell culture system: induction and maintenance of estradiol production, response to follicle-stimulating hormone, and morphological characteristics. Biol Reprod.

[B62] Austin EJ, Mihm M, Evans AC, Knight PG, Ireland JL, Ireland JJ, Roche JF (2001). Alterations in intrafollicular regulatory factors and apoptosis during selection of follicles in the first follicular wave of the bovine estrous cycle. Biol Reprod.

[B63] Fortune JE, Rivera GM, Evans AC, Turzillo AM (2001). Differentiation of dominant versus subordinate follicles in cattle. Biol Reprod.

[B64] Ginther OJ, Bergfelt DR, Beg MA, Kot K (2002). Role of low circulating FSH concentrations in controlling the interval to emergence of the subsequent follicular wave in cattle. Reproduction.

[B65] Ireland JJ, Mihm M, Austin E, Diskin MG, Roche JF (2000). Historical perspective of turnover of dominant follicles during the bovine estrous cycle: key concepts, studies, advancements, and terms. J Dairy Sci.

[B66] Mihm M, Evans AC (2008). Mechanisms for dominant follicle selection in monovulatory species: a comparison of morphological, endocrine and intraovarian events in cows, mares and women. Reprod Domest Anim.

[B67] Quintal-Franco JA, Kojima FN, Melvin EJ, Lindsey BR, Zanella E, Fike KE, Wehrman ME, Clopton DT, Kinder JE (1999). Corpus luteum development and function in cattle with episodic release of luteinizing hormone pulses inhibited in the follicular and early luteal phases of the estrous cycle. Biol Reprod.

[B68] Schams D, Berisha B, Kosmann M, Einspanier R, Amselgruber WM (1999). Possible role of growth hormone, IGFs, and IGF-binding proteins in the regulation of ovarian function in large farm animals. Domest Anim Endocrinol.

[B69] Fabbri M, Garzon R, Cimmino A, Liu Z, Zanesi N, Callegari E, Liu S, Alder H, Costinean S, Fernandez-Cymering C (2007). MicroRNA-29 family reverts aberrant methylation in lung cancer by targeting DNA methyltransferases 3A and 3B. Proc Natl Acad Sci USA.

[B70] Shen WF, Hu YL, Uttarwar L, Passegue E, Largman C (2008). MicroRNA-126 regulates HOXA9 by binding to the homeobox. Mol Cell Biol.

[B71] Shi B, Sepp-Lorenzino L, Prisco M, Linsley P, deAngelis T, Baserga R (2007). Micro RNA 145 targets the insulin receptor substrate-1 and inhibits the growth of colon cancer cells. J Biol Chem.

[B72] Shi XB, Xue L, Yang J, Ma AH, Zhao J, Xu M, Tepper CG, Evans CP, Kung HJ, deVere White RW (2007). An androgen-regulated miRNA suppresses Bak1 expression and induces androgen-independent growth of prostate cancer cells. Proc Natl Acad Sci USA.

[B73] Tili E, Michaille JJ, Cimino A, Costinean S, Dumitru CD, Adair B, Fabbri M, Alder H, Liu CG, Calin GA (2007). Modulation of miR-155 and miR-125b levels following lipopolysaccharide/TNF-alpha stimulation and their possible roles in regulating the response to endotoxin shock. J Immunol.

[B74] Iorio MV, Visone R, Di Leva G, Donati V, Petrocca F, Casalini P, Taccioli C, Volinia S, Liu CG, Alder H (2007). MicroRNA signatures in human ovarian cancer. Cancer Res.

[B75] Yang H, Kong W, He L, Zhao JJ, O'Donnell JD, Wang J, Wenham RM, Coppola D, Kruk PA, Nicosia SV (2008). MicroRNA expression profiling in human ovarian cancer: miR-214 induces cell survival and cisplatin resistance by targeting PTEN. Cancer Res.

[B76] Iorio MV, Ferracin M, Liu CG, Veronese A, Spizzo R, Sabbioni S, Magri E, Pedriali M, Fabbri M, Campiglio M (2005). MicroRNA gene expression deregulation in human breast cancer. Cancer Res.

[B77] Murakami Y, Yasuda T, Saigo K, Urashima T, Toyoda H, Okanoue T, Shimotohno K (2006). Comprehensive analysis of microRNA expression patterns in hepatocellular carcinoma and non-tumorous tissues. Oncogene.

[B78] Nam EJ, Yoon H, Kim SW, Kim H, Kim YT, Kim JH, Kim JW, Kim S (2008). MicroRNA expression profiles in serous ovarian carcinoma. Clin Cancer Res.

[B79] Pan Q, Luo X, Toloubeydokhti T, Chegini N (2007). The expression profile of micro-RNA in endometrium and endometriosis and the influence of ovarian steroids on their expression. Mol Hum Reprod.

[B80] Griffiths-Jones S (2004). The microRNA Registry. Nucleic Acids Res.

[B81] Griffiths-Jones S, Grocock RJ, van Dongen S, Bateman A, Enright AJ (2006). miRBase: microRNA sequences, targets and gene nomenclature. Nucleic Acids Res.

[B82] miRBase_12.0. http://microrna.sanger.ac.uk/sequences/.

[B83] BLAST cow sequences. http://www.ncbi.nlm.nih.gov/genome/seq/BlastGen/BlastGen.cgi?taxid=9913.

[B84] Genomic tRNA database. http://lowelab.ucsc.edu/GtRNAdb/.

[B85] Ribosomal RNA BLAST. http://bioinformatics.psb.ugent.be/webtools/rRNA/blastrrna.html.

[B86] Blast ncRNA database. http://ncrnadb.trna.ibch.poznan.pl/blast.html.

[B87] RNAdb. http://research.imb.uq.edu.au/rnadb/default.aspx.

[B88] tRNAscan-SE. http://lowelab.ucsc.edu/tRNAscan-SE/.

[B89] Mfold web server_3.2. http://frontend.bioinfo.rpi.edu/applications/mfold/cgi-bin/rna-form1.cgi.

[B90] Zuker M (2003). Mfold web server for nucleic acid folding and hybridization prediction. Nucleic Acids Res.

[B91] Ro S, Park C, Jin J, Sanders KM, Yan W (2006). A PCR-based method for detection and quantification of small RNAs. Biochem Biophys Res Commun.

[B92] Obernosterer G, Martinez J, Alenius M (2007). Locked nucleic acid-based in situ detection of microRNAs in mouse tissue sections. Nat Protoc.

[B93] miRBase Targets Version 5. http://microrna.sanger.ac.uk/targets/v5/.

[B94] Delfour O, Vilanova D, Atzorn V, Michot B, Clarke NJ (2007). The passionate race for miRNA detection and function deciphering. miRNA: Biology, Function and Expression.

