# Supplementary material for: Identification and characterization of miRNAs expressed in the bovine ovary
Source: BMC Genomics. 2009 Sep 18;10:443. doi: 10.1186/1471-2164-10-443 (PMC2762473; doi:10.1186/1471-2164-10-443)
Supplement: Additional file 2 — Predicted secondary structure of new miRNAs and detection of expression in multiple tissues. [file 1471-2164-10-443-S2.PDF]

Supplementary Table 1. Predicted secondary structure of new miRNAs

1.
```
>bomir-22*/22-5p:Btau_4.0:19:22901896:22901976:1(10-31) dG = -31.40 kcal/mol
CAGAGGGCAacaguucuucaacuggcagcuuUAGCUGGGUCAGGACAuaaagcuugccacugaagaacuACUGCGGCUCAG
     CA   G-   ac         ac     -      GC  GGU
       GAG  GCA  aguucuuca  uggca gcuuUA  UG   C
       |||  |||  |||||||||  ||||| ||||||  ||   |
       CUC  CGU  ucaagaagu  accgu cgaaau  AC   A
     GA   GG   CA         c-     u      --  AGG
```

2.
```
>bomir-140*/140-5p:Btau_4.0:18:35987036:35987127:1(17-38) dG = -51.10 kcal/mol
UCUCUCUGUGUCCUGCcagugguuuuacccuaugguagGUUACGUCAUGCUGUUCuaccacaggguagaaccacggACAGGAUACCGGGGCA
     UC    U        -  a             a       UU   UC
       UCUC GUGUCCUG Cc gugguuuuacccu ugguagG  ACG  A
       |||| |||||||| || ||||||||||||| |||||||  |||  |
       GGGG CAUAGGAC gg caccaagauggga accauCU  UGU  U
     AC    C        A  -             c       --   CG
```

3.
```
>bomir-143-3p:Btau_4.0:7:60268810:60268888:1(57-78) dG = -48.50 kcal/mol
UCUCCCAGCCUGAGGUGCAGUGCUGCAUCUCUGGUCAGUUGGGAGUCugagaugaagcacuguagcucgGGAAGGGAGA
           AG      G         G      U  -  AG
     UCUCCC  CCUGAG UGCAGUGCU CAUCUC GG UC  U
     ||||||  |||||| ||||||||| |||||| || ||  |
     AGAGgg  gggcuc augucacga guagag CU AG  U
           aa      g         a      u  G  GG
```

4.
```
>bomir-152-5p:Btau_4.0:19:39650354:39650424:-1(6-26) dG = -38.00 kcal/mol
CGGGCccaaguucugucaugcacugaCUGCUCCAGAGCCCGAGUCGGAGUGUAUCACAGAACCUGGGCCGG
     CG      a       c       --    ---   C
       GGCcca guucugu augcacu  gaCU   GCU C
       |||||| ||||||| |||||||  ||||   ||| |
       CCGGGU CAAGACA UAUGUGA  CUGA   CGA A
     GG      C       C       GG    GCC   G
```

5.
```
>bomir-193a-2-3p:Btau_4.0:14:889826:889878:-1(32-50) dG = -17.80 kcal/mol
AUGGCUGCCUCACAAGGUUUGGAGCUGUGCCUgggacuuuguaggccaguuCA
     AU    -    C       - UG A  U
       GGCU GCCU ACAAGGU U  G GC \
       |||| |||| ||||||| |  | ||  G
       uuga cgga uguuuca g  C CG /
     AC    c    -       g gU -  U
```

6.
```
>bomir-378-1-3p:Btau_4.0:7:60536464:60536541:1(49-69) dG = -47.70 kcal/mol
CCACCCAGGGCUCCUGACUCCAGGUCCUGUGUGUUACCUCGAAAUAGCAcuggacuuggagucagaaggcCUGAGUGG
         C    G  C              UGU      CCU
     CCAC CAGG CU CUGACUCCAGGUCC   GUGUUA   C
     |||| |||| || ||||||||||||||   ||||||   |
     GGUG GUCc ga gacugagguucagg   cACGAU   G
         A    g  a              u--      AAA
```

7.
```
> bomir-378-2-5p:Btau_4.0:4:11116890:11116965:1(9-29) dG = -14.80 kcal/mol
GGAGAGCAcuggacuuggagucagaaggcUGGAGCUUACAGGGCAGCACCGUCAUCUACUGGUGGAGAACUACGCC
       AG-  CA  gg   ugg   c   a   U  A   UAC
     GG   AG  cu  acu   agu aga ggc GG GCU   A
     ||   ||  ||  |||   ||| ||| ||| || |||   |
     CC   UC  GA  UGG   UCA UCU CUG CC CGA   G
       GCA  AA  GG   ---   -   A   -  A   CGG
```

8.
```
>bomir-382-3p:Btau_4.0:21:66031742:66031827:-1 (51-71)dG = -28.30 kcal/mol
UUUGGUACUGAAAAAAGUGUUGUCCGUGAAUGAUUCGUCAUAAGUAAAGCgaauccaccacgaacaacuucUCUUCAAGUACCACA
     UU      -    AAAAGU     C    AAU       CAUA
       UGGUAC UGAA      GUUGU CGUG   GAUUCGU    A
       |||||| ||||      ||||| ||||   |||||||    |
       ACCAUG ACUU      caaca gcac   cuaagCG    G
     AC      A    CUcuu-     a    cac       AAAU
```

9.
```
>bomir-409-5p:Btau_4.0:21:66042116:66042194:-1(13-33) dG = -33.60 kcal/mol
UGAUACCGAAAAggguucaccgagcaacauucGUCGUCCAGAUGCAAAGUUGCUCGGGUAACCUCUCCCCGCGUACCA
     U A   -  AAA       ca         a  -    GU
      G UAC CG   Agggguu  ccgagcaac uu cGUC  \
      | ||| ||   |||||||  ||||||||| || ||||  C
      C AUG GC   UCUCCAA  GGCUCGUUG AA GUAG  /
     A C   C  CCC       UG         A  C    AC
```

10.
```
>bomir-424-3p:Btau_4.0:Un.004.53:446853:446952:-1(59-79) dG = -46.20 kcal/mol
UUCGUUGACUCCGAGGGGAUGCAGCAGCAAUUCAUGUUUUGAAGUGCUUUAAACGGUUcaaaacgugaggcgcugcuauACCCCCUUGCGAGGAAGUAGG
     U CG  GA   -       A  C      AA             G  CUU
      U  UU  CUC CGAGGGG UG AGCAGC  UUCAUGUUUUGAA UG   \
      |  ||  ||| ||||||| || ||||||  ||||||||||||| ||   U
      G  GA  GAG GUUCCCC Au ucgucg  gagugcaaaacUU GC   /
     G AU  AG   C       C  a      cg             G  AAA
```

12.
```
>bomir-542-3p:Btau_4.0:Un.004.53:441560:441637:1(45-67) dG = -25.80 kcal/mol
CCCAGACCUUUCAGUUAUCAAUCUGUCACAAGUGCACAGUGGUAucucgugacaugaugaucccgaGAUGUCUGAGG
       -     C    A---       AUC      A  -   AC
     CC CAGAC UUUC    GUUAUCA   UGUCAC AG UGC  A
     || ||||| ||||    |||||||   |||||| || |||  |
     GG GUCUG AGag    uaguagu   acagug uc AUG  G
       A     U    cccc       ---      c  u   GU
```

13.
```
>bomir-574-5p:Btau_4.0:16:59370641:59370704:-1(7-28) dG = -25.20 kcal/mol
AGAGUGugugggugugugcaugugcgugUGUGCACAUGCAUAUGUGUGUGGCUAUCUUAGCUGU
     AG   Gu--    g  ug             G
       AGU    gugg ug  ugcaugugcgugU U
       |||    |||| ||  ||||||||||||  |
       UCG    UAUC GU  GUGUAUACGUACA G
     UG   AUUC    G  GU             C
```

14.
```
>bomir-652-3p: hsa:X:109185213-109185310:-1, dG = -50.10 kcal/mol
CACAUCCAACACGUGACguguugggaucaccgcgguaaGUUAAUAUCAGAUACACUUACCGUGGGAGAGGAUCCCAACACGUCACGUAUCGGUAAGCA
   CACAU-  AAC                  a----           UAAUAU
         CC   ACGUGACguguugggauc     ccgcgguaaGU      \
         ||   ||||||||||||||||||     |||||||||||      C
         GG   UGCACUGCACAACCCUAG     GGUGCCAUUCA      /
   ACGAAU  CUA                  GAGAG           CAUAGA
```

15.
```
>bomir-940-5p:Btau_4.0:20:75274429:75274494:-1(3-20) dG = -39.80 kcal/mol
CCgcagggcccccgcuccccGUGGCCACCAGAGUGCCCUGUGGGGAGGCGGGGGAGCCACCUGGGG
       g    g----      -       UG C-   C
     CC cagg     cccccg cuccccG  G  CAC A
     || ||||     |||||| |||||||  |  ||| |
     GG GUCC     GGGGGC GAGGGGU  C  GUG G
       G    ACCGA      G       GU CC   A
```

16.
```
>bomir-F0131-5p:Btau_4.0:7:10905950:10906020:1(16-33) dG = -29.40 kcal/mol
UGUGUUUGGGAGUUGggggcgggggggcgggugGAUUCCAGCUGUCUUCCCCUCGUGGACACUGUGACAUA
           UG  A   Gg-   c          g   A
     UGUGUU  GG GUU   ggg gggggggcgg ugG \
     ||||||  || |||   ||| |||||||||| ||| U
     AUACAG  UC CAG   CUC CCCUUCUGUC ACC /
           UG  A   GUG   -          G   U
```

17.
```
>bomir_F0132-5p:Btau_4.0:7:13891717:13891774:1(2-20) dG = -23.40 kcal/mol
CagcccgggccccuccccugGCUCCGCCCACAGCAUCAUGGGACCCAGCCCAGGGCCU
     Ca    -    ccc-    c  --   CCG
       gccc gggc    uccc ug  GCU   C
       |||| ||||    |||| ||  |||   |
       CGGG CCCG    AGGG AC  CGA   C
     UC    A    ACCC    U  UA   CAC
```

18
```
>bomir-H0121-3p:Btau_4.0:18:7202568:7202632:1(43-60) dG = -27.60 kcal/mol
GAGGCGGCCAGACCAGCAGGGCGCAGGGGACCAUGUGUCCUCcuucccguguguugagccGCGUG
     GAG     CA   CA   -   CGC   -    CA
        GCGGC  GAC  GCA GGG   AGG GGAC  \
        |||||  |||  ||| |||   ||| ||||  U
        CGccg  uug  ugu ccc   ucC CCUG  /
     GUG     ag   --   g   u--   U    UG
```

19.
```
>bomir-F0244-5p:Btau_4.0:12:45758242:45758332:-1(15-33) dG = -20.10 kcal/mol
GCCACUGCCCAUUgcuacuaccgauuggauggUUUAGUGUUGCCCUUGGAUAAUCCAAUAAGUAGAAUUAAUAGGUGAAAAGUUGUGAGC
       -   U  ------  C    cua    cg       g      UGU
     GC CAC GC      CC AUUg   cuac  auuggau gUUUAG   U
     || ||| ||      || ||||   ||||  ||||||| ||||||   |
     CG GUG UG      GG UAAU   GAUG  UAACCUA UAGGUU   G
       A   U  AAAAGU  A    UAA    AA       A      CCC
```

20
```
>bomir-H0222-3p:Btau_4.0:29:41706131:41706188:-1(30-48) dG = -31.50 kcal/mol
UCUGGGGCGCCCCGCACCGGCGGGCAGGAcggcggcagcgccggggcgCCGUCUCGGA
          ---        -  A---    G  GGC
     UCUGG   GGCGCCCC GC    CCG CG   \
     |||||   |||||||| ||    ||| ||    A
     AGGCU   CCgcgggg cg    ggc gc   /
          CUG        c  cgac    g  AGG
```

21.
```
>bomir-A0321-3p:Btau_4.0:19:39110468:39110542:1(40-57) dG = -20.40 kcal/mol
CCGGCCUACUUGGGGCUCCGAGCCGCUGCCGCAUACAUUagcgccgccggccgcaccGCCGUCACCUUCAGCGCU
     CC   -  ACU--   -   UC--  A    C  C   AUA
       GGC CU     UGG GGC    CG GCCG UG CGC   C
       ||| ||     ||| |||    || |||| || |||   |
       UCG GA     ACU CCG    gc cggc gc gcg   A
     --   C  CUUCC   G   ccac  -    c  c   aUU
```

22.
```
>bomir-C0533-1-5p:Btau_4.0:18:59928730:59928786:1(4-23) dG = -26.70 kcal/mol
CGUcgggaccgggguccggugcgGAGAGCCCUUCGUCCCGGGACACGGGGCGCGGCC
     C    gga-   ggg     ug     A
      GUcg    ccg   uccgg  cgGAG G
      ||||    |||   |||||  ||||| |
      CGGC    GGC   GGGCC  GCUUC C
     C    GCGG   ACA     CU     C
```

23.
```
>bomir-C0533-2-5p:Btau_4.0:21:52041884:52041940:-1(4-23) dG = -26.70 kcal/mol
CGUcgggaccgggguccggugcgGAGAGCCCUUCGUCCCGGGACACGGGGCGCGGCC
     C    gga-   ggg     ug     A
      GUcg    ccg   uccgg  cgGAG G
      ||||    |||   |||||  ||||| |
      CGGC    GGC   GGGCC  GCUUC C
     C    GCGG   ACA     CU     C
```

24.
```
>bomir-F0522-1-3p:Btau_4.0:21:35870333:35870398:1(47-65) dG = -27.40 kcal/mol
CCUCUCUCCCCUGCAGCAUCUCAGGGCACUGUGUGACUUGGGUCGGGggugggguggggggguuggU
     -  UC      UG AG      AG   A   UGU
      CC  UCUCCC  C  CAUCUC  GGC CUG   \
      ||  ||||||  |  ||||||  ||| |||   G
      gg  gggggg  g  guggGG  CUG GGU   /
     U  uu      gu gg      G-   -   UCA
```

25.
```
>bomir-F0522-2-3p:Btau_4.0:22:59347353:59347420:1(43-61) dG = -34.40 kcal/mol
GGAGGGUGCCUCUAAGCCUCACUUGUCCUGCCUGUCAAAUGGgugggguggggggguuggCCUUUCC
           U   UCUA      A  UG   UG   GUC
     GGAGGG GCC    AGCCUC CU  UCC  CCU   A
     |||||| |||    |||||| ||  |||  |||   |
     CCUUUC Cgg    uugggg gg  ggg  ggG   A
           -   ----      -  gu   gu   GUA
```

26.
```
>bomir-B0821-5p:Btau_4.0:20:19373708:19373768:-1(3-23) dG = -32.60 kcal/mol
GCguccccggggcucccgccggcGGAGUUCGGUUUGCACCGGGGAGGGGGCCGGGGAUCGG
     G  -     gg       g  gg   A  UCG
      Cg ucccc  ggcuccc cc  cGG GU   \
      || |||||  ||||||| ||  ||| ||   G
      GC AGGGG  CCGGGGG GG  GCC CG   /
     G  U     --       A  G-   A  UUU
```

27.
```
>bomir-F1351-3p:Btau_4.0:25:41129445:41129520:1(53-72) dG = -44.20 kcal/mol
CGGCGGCCGGGAAAGGUGGGCUGGGCUCCGGGGCCCCUCCCCGCCCGGUGCUgccccggccgcucccggccuUGUG
     CGGC-        AA   --    UG   U     G CCC
          GGCCGGGA  GGU  GGGC  GGC CCGGG C   U
          ||||||||  |||  ||||  ||| ||||| |   |
          ccggcccu  ccg  cccg  UCG GGCCC G   C
     GUGUu        cg   gc    --   U     - CCC
```

28.
```
>bomir-F1353-5p:Btau_4.0:28:27885032:27885119:1(5-24) dG = -18.30 kcal/mol
AAAAaucuuugggcuagguuaguuCUAUUUUAUGAUCUGUUAUGAAAUGGGUUAAGGAAAAGGAUCCAUGUGGAAGA
AAGAUCAGAGA
     AAAAA-      ggg   g  ua     A   UA         A
           aucuuu   cua gu  guuCU UUU  UGAUCUGUU U
           ||||||   ||| ||  ||||| |||  ||||||||| |
           UAGAAA   GGU UA  UAGGA AAG  AUUGGGUAA G
     AGAGAC      GAA   G  CC     A   GA         A
```

29.
```
>bomir-D1431-5p:Btau_4.0:12:75101950:75102053:-1(3-24) dG = -44.60 kcal/mol
GUggcgacggaggcgcgacccccccCCCCCCCCCCCCCCCCCGCCCCGGUCCGACGGUGCGACCCGCCAGGGGCGCUC
UGGGGACAGUCCGCCCCGCCCCGCCC
     GU    a--    a    c   cc-    CCCC--    CCCCCCCC   C   GU
       ggcg   cgg ggcg gac   cccC      CCCC         CGC CCG  C
       ||||   ||| |||| |||   ||||      ||||         ||| |||  |
       CCGC   GCC CCGC CUG   GGGG      GGGG         GCG GGC  C
     C-    CCC    -    -   ACA    UCUCGC    ACCGCCCA   U   AG
```

30.
```
>bomir-C1511-5p:Btau_4.0:Un.004.1059:20638:20698:1(2-21) dG = -32.00 kcal/mol
CguggaggagaaugcccggggCGUGCUGGGCGGUGCGGGCUGGGCUGGCAGCUCCUCCCUC
     Cgu       aau---      gg  UG  G
        ggaggag      gcccgg  CG  CU G
        |||||||      ||||||  ||  || |
        CCUCCUC      CGGGUC  GC  GG G
     CUC       GACGGU      GG  GU  C
```

31.
```
>bomir-F1821-3p:Btau_4.0:Un.004.152:123132:123213:1(60-80) dG = -31.60 kcal/mol
CGCAACGAGGGCUCGGGACACAGCCCUGCUUCCUGGCCCACGUCACCCGUCGUGGCCAGagcccuggcccugccaucgugUC
     C  A    G   UC   ACA   CCCU    UC      C   UCA
      GC ACGA GGC  GGG   CAG    GCU  CUGGCC ACG   C
      || |||| |||  |||   |||    |||  |||||| |||   |
      Ug ugcu ccg  ccc   guc    cga  GACCGG UGC   C
     C  -    a   u-   g--   c---    --      -   UGC
```

32.
```
>bomir-C1931-5p:Btau_4.0:26:12405822:12405907:1(17-39) dG = -11.60 kcal/mol
CCCUGAGUUGCAAUUCccugcugaucucacauuaauucaUCUUUGCUAGAGAAAUAUCUGAGUGUCUGUUUUGGUUCAAUAAAGCC
     CC  GA    C    Cccu  u   -    cau  a   a   UU
       CU  GUUG AAUU    gc gau cuca   ua uuc UCU  \
       ||  |||| ||||    || ||| ||||   || ||| |||  G
       GA  UAAC UUGG    UG CUG GAGU   AU AAG AGA  /
     CC  AA    -    UUU-  U   U    CU-  A   -   UC
```

33.
```
>bomir-A2143-3p:Btau_4.0:16:47801303:47801359:1(34-51) dG = -18.50 kcal/mol
ACACCCGGCUGGCGUCAACUCUGAACAGAUUCUcggcagaugaaguccaucGGGAAG
     ACA     C   - G   A  -    ACA
        CCCGG UGG C UCA CU CUGA   G
        ||||| ||| | ||| || ||||   |
        GGGcu acc g agu ga ggcU   A
     GAA     -   u a   a  c    CUU
```

34.
```
>bomir-F2422-5p:Btau_4.0:18:53584142:53584210:1(1-20) dG = -35.50 kcal/mol
gguggggagggucccaccgagCACCCUGUCGGCUGGCAGCCCCGGCCCAGGUAGUGGGUGCUCUGCCACA
     g    g     -     cgagCAC    UC      CA
      gugg agggu cccac       CCUG  GGCUGG  \
      |||| ||||| |||||       ||||  ||||||  G
      CACC UCUCG GGGUG       GGAC  CCGGCC  /
     A    G     U     AU-----    --      CC
```

35.
```
>bomir-F2531-3p:Btau_4.0:8:77307612:77307688:1(50-67) dG = -13.80 kcal/mol
GGUUUAAUGCUCUGCUGUCAGCGCUUUGAAAUUCUUACUAAUCUUUUUUugguggagaugccggggaCGUAAUAAUU
     GGUUUA-   -     CU   AG    UU    UUCUUA
            AUG CUCUG  GUC  CGCU  GAAA      \
            ||| |||||  |||  ||||  ||||      C
            UGC ggggc  uag  gugg  UUUU      /
     UUAAUAA   a     cg   ag    uU    UCUAAU
```

36.
```
>bomir-G2511-3p:Btau_4.0:18:41190497:41190564:1(40-57) dG = -21.10 kcal/mol
AGGGGGAGAGUUGCGGCUCAUGAACUGGGUCCAGGUGGAaggcgggccgggguuggaAGGUUCCAGCU
     AGGG    AG  G      A  AA   GG-   A
         GGAG  UU CGGCUC UG  CUG   UCC G
         ||||  || |||||| ||  |||   ||| |
         CCUU  Aa guuggg gc  ggc   AGG G
     UCGA    GG  g      -  cg   gga   U
```

37.
```
>bomir-E2664-3p:Btau_4.0:18:64360999:64361052:-1(33-52) dG = -21.10 kcal/mol
AGUCAAGUGAGCGGCCGCACCGGCUCUCCCGGagggcgggcggcgacuggaaAG
     AG-  A   GAGCG     A   G     C
        UC AGU     GCCGC CCG CUCUC \
        || |||     ||||| ||| |||||  C
        ag uca     cggcg ggc gggaG /
     GAa  g   g----     -   -     G
```

38.
```
>bomir-D3011-3p:Btau_4.0:18:39424877:39424959:1(62-82) dG = -47.50 kcal/mol
ACGCGCUUCUCGGCGCUCACUCGGCGGCACCACUGCCGCAAAUGCGGCUUCGUGGUCUGCGccgagugcucccgcgagcgcuU
     AC    U  U   CGCU         GC-     U--     A
       GCGC UC CGG    CACUCGGCG   ACCAC   GCCGC A
       |||| || |||    |||||||||   |||||   ||||| |
       cgcg ag gcc    gugagccGC   UGGUG   CGGCG A
     Uu    -  c   cuc-         GUC     CUU     U
```

39.
```
>bomir-A3341-1-3p:Btau_4.0:3:124987968:124988032:1(41-61) dG = -28.00 kcal/mol
UCACCCCAGUCUCUAGUGAGAGAGCCAUGGCGGCAGGGGCguggcugucccuggaggugggACUG
     UCAC    G       UGA  G       G GGC
         CCCA UCUCUAG   GA AGCCAUG C   \
         |||| |||||||   || ||||||| |    A
         gggu ggagguc   cu ucggugC G   /
     GUCA    -       c--  g       - GGG
```

40.
```
>bomir-A3341-2-3p:Btau_4.0:Un.004.4799:1295:1359:1(41-61) dG = -28.00 kcal/mol
UCACCCCAGUCUCUAGUGAGAGAGCCAUGGCGGCAGGGGCguggcugucccuggaggugggACUG
     UCAC    G       UGA  G       G GGC
         CCCA UCUCUAG   GA AGCCAUG C   \
         |||| |||||||   || ||||||| |    A
         gggu ggagguc   cu ucggugC G   /
     GUCA    -       c--  g       - GGG
```

41.
```
>bomir-A3711-5p:Btau_4.0:9:63475787:63475874:1(18-36) dG = -64.40 kcal/mol
CGCCGCUCCGCUCCCGGuuccgcgcucuacgccagcCCGCUGCCUGCCGGGCUGGUGCAGGCCGCGGAGCCGGGCGGCCCGGCAGGCA
     C   -   CC   -            cu  a         -  UG
      GCC GCU  GCU CCCGGuuccgcg  cu cgccagcCC GC  \
      ||| |||  ||| ||||||||||||  || ||||||||| ||   C
      CGG CGG  CGG GGGCCGAGGCGC  GA GUGGUCGGG CG  /
     A   A   CC   C            CG  C         C  UC
```

42.
```
>bomir-A4052-1-5p:Btau_4.0:18:59928617:59928686:1(14-32) dG = -38.20 kcal/mol
ACGGCAGCGCCGCgggagccucgguuggccucGGAUAGCCGGUCCCCCGCCGUCCCCGCCGGCGGGCCGU
          AG     -    ag cu   uu  cc    A
     ACGGC  CGCCG Cggg  c  cgg  gg  ucGG U
     |||||  ||||| ||||  |  |||  ||  |||| |
     UGCCG  GCGGC GCCC  G  GCC  CC  GGCC A
          G-     C    CU CC   --  CU    G
```

43.
```
>bomir-A4052-2-5p:Btau_4.0:21:52041984:52042053:-1(14-32) dG = -38.20 kcal/mol
ACGGCAGCGCCGCgggagccucgguuggccucGGAUAGCCGGUCCCCCGCCGUCCCCGCCGGCGGGCCGU
          AG     -    ag cu   uu  cc    A
     ACGGC  CGCCG Cggg  c  cgg  gg  ucGG U
     |||||  ||||| ||||  |  |||  ||  |||| |
     UGCCG  GCGGC GCCC  G  GCC  CC  GGCC A
          G-     C    CU CC   --  CU    G
```

44.
```
>bomir-A4052-3-5p:Btau_4.0:Un.004.2732:16031:16100:-1(14-32) dG = -38.20 kcal/mol
ACGGCAGCGCCGCgggagccucgguuggccucGGAUAGCCGGUCCCCCGCCGUCCCCGCCGGCGGGCCGU
          AG     -    ag cu   uu  cc    A
     ACGGC  CGCCG Cggg  c  cgg  gg  ucGG U
     |||||  ||||| ||||  |  |||  ||  |||| |
     UGCCG  GCGGC GCCC  G  GCC  CC  GGCC A
          G-     C    CU CC   --  CU    G
```

45.
```
> bomir-106-2-5p:Btau_4.0:Un.004.53:181658:181738:-1(14-34) dG = -30.10 kcal/mol
CCUUGGCCAUGUAaaagugcuuacagugcagguaGCUUUUUGAGAUCUACUGCAAUGCAAGCACUUCUUACAUUACCAUGG
       U   CC      -        a  g     g   C  UU
     CC UGG  AUGUAa aagugcuu ca ugcag uaG UU  \
     || |||  |||||| |||||||| || ||||| ||| ||   U
     GG ACC  UACAUU UUCACGAA GU ACGUC AUC AG  /
       U   AU      C        C  A     -   U  AG
```

46.

```
>bomir-24-3-3p:Btau_4.0:8:85962803:85962876:1(47-71) dG = -27.20 kcal/mol
GCCCUCCGGUGCCUACUGAGCUGAUAUCAGUUCUCAUUUUACACACuggcucaguucagcaggaacaggagUCG
     GCC    G  G   A         UA     UCUCAU
        CUCC GU CCU CUGAGCUGA  UCAGU      \
        |||| || ||| |||||||||  |||||       U
        gagg ca gga gacuugacu  gguCA      /
     GCU    a  a   c         c-     CACAUU
```

47.

```
>bomir-26-2-3p:Btau_4.0:22:11464097:11464186:-1(54-75) dG = -34.00 kcal/mol
CAGGCCCGCGUCCGCGUGCAAGUAACCGAGAAUAGGCCCCUUGGGACCUGCACagccuauccuggauuacuugaaCGAGGCCACGGCCUU
     C     CGC U  G   G       -  G  A      ---------  C
      AGGCC   G CC CGU CAAGUAA CC AG AUAGGC         CC U
      |||||   | || ||| ||||||| || || ||||||         || |
      UCCGG   C GG GCa guucauu gg uc uauccg         GG U
     U     CAC -  A   a       a  -  c      aCACGUCCA  G
```

48.

```
>bomir-199a-2-5p:Btau_4.0:7:13733556:13733633:1(8-29) dG = -33.50 kcal/mol
CCCAGCCuaaccaaugugcagacuacuguACACAUUCAGAGCCCCCUGAACAGGUAGUCUGAACACUGGGUUGGCGGG
        A   uaa   a   g         guACACA     AG
     CCC GCC   cca ugu cagacuacu       UUCAG  C
     ||| |||   ||| ||| |||||||||       |||||  |
     GGG CGG   GGU ACA GUCUGAUGG       AAGUC  C
        -   UUG   C   A         AC-----     CC
```

49.

```
>bomir-199a-3-5p:Btau_4.0:11:102419020:102419092:1(6-27) dG = -21.00 kcal/mol
CAGCCuaaccaaugugcagacuacuguACAAUUUGGGAGUCCUGAACAGAUAGUCUAAACACUGGGUAGACGG
     CAGC   a   a   gc      -    ACAAU UG  A
         Cua cca ugu  agacua cugu     U  GG \
         ||| ||| |||  |||||| ||||     |  ||  G
         GAU GGU ACA  UCUGAU GACA     A  CC /
     GGCA   G   C   AA      A    ----- GU  U
```

50.

```
>bta-let-7b-2-3p:Btau_4.0:5:123308015:123308097:-1(60-81) dG = -22.90 kcal/mol
CUCGAGGAAGGCAGUAGGUUGUAUAGUUAUCUUCCGAGGGGGCAACAUCACUACCCUGAaaccacacaaccuacuaccucaCC
     GAC    AAGGC          AUA   A  UUCCG  G  GC
        GAGG     AGUAGGUUGU   GUU UC      AG GG  A
        ||||     ||||||||||   ||| ||      || ||  |
        cucc     ucauccaaca   caa AG      UC CU  A
     CCa    a----          cac   -  UCCCA  A  AC
```

Secondary structure for new miRNAs and new loci of annoated miRNAs predicted by mfold web server. First line started with the name of the miRNA followed by database (Ensemble 52; Btau_4.0), genomic location (chromosome number: start: end: sense or antisense orientationand orientation, (position of the mature sequence in the precursor) and minimum free energy. Position of the mature sequence in the hairpin precursor is indicated as lower case and by the red color.

Supplementary figure 2. Detection and expression analysis of selected miRNAs in multiple tissues by PCR approach.

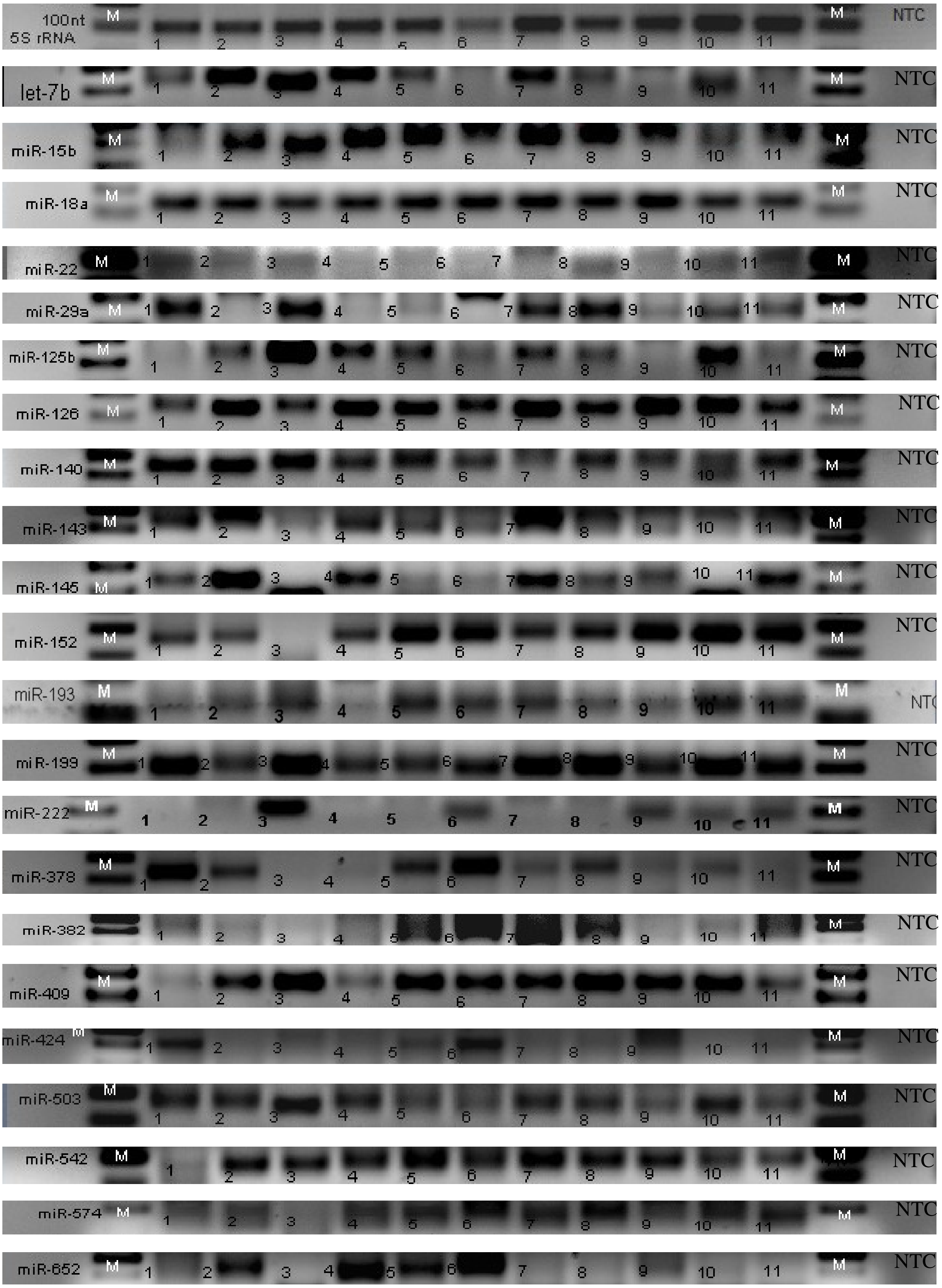

Figures Continued

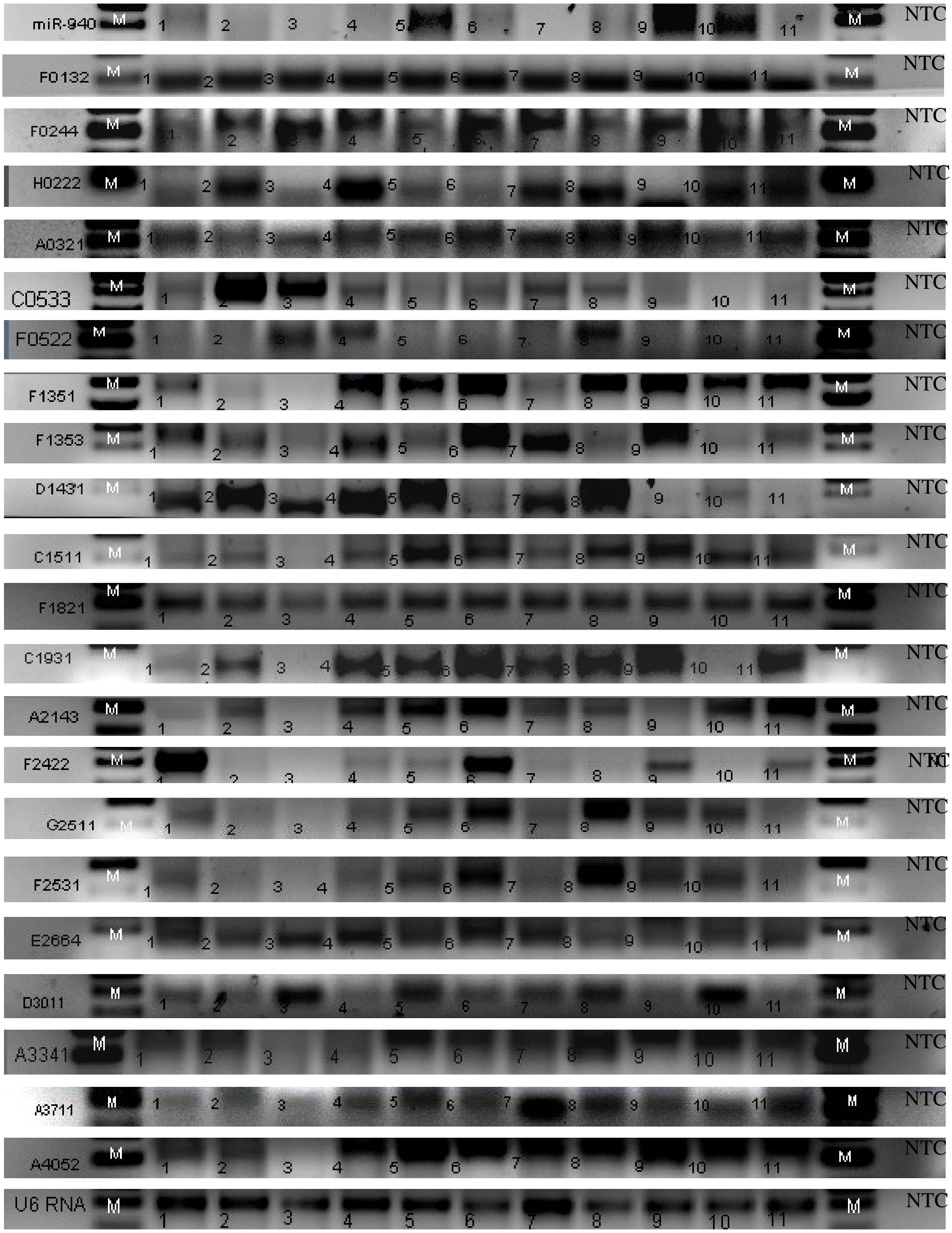

Expression profiles of 44 miRNAs including new miRNAs in multiple tissues by PCR approach. Amplicons were analyzed on 2% agarose gel. 5S rRNAs and U6 RNA were used as a loading control. A DNA ladder (M) indicating the size of the fragments (50-100-150 nt) on each side. (NTC) Non-template control. The number 1-11 denote different tissues and cells where, 1-Ovarian cortex corpus luteum, 2 – Fetal ovary, 3 – Cumulus cells, 4 – Corpus luteum, 5 – Oviduct, 6 – Uterus, 7 – Placenta, 8 – Heart, 9 – Liver, 10 – Lung, 11 – Spleen.
