# Supplementary material for: Identification and characterization of miRNAs expressed in the bovine ovary
Source: BMC Genomics. 2009 Sep 18;10:443. doi: 10.1186/1471-2164-10-443 (PMC2762473; doi:10.1186/1471-2164-10-443)
Supplement: Additional file 3 — Screened target genes list for cloned miRNAs and figures of GO analysis. [file 1471-2164-10-443-S3.PDF]

Supplementary table 1. Comprehensive target list consisting of 115 potential genes after filtering from all the predicted target genes for the identified miRNAs

Sl. No.	*GENE SYMBOL*	*Entrez Gene Name*	Tissues/Cells	Relevant references	Predicted to be targeted by (only identified miRNAs were picked)	Score/Base P value from miRBase target version_5.0	Energy (miRBase target version_5.0
1	*CCNB1*	*cyclin B1*	Oocytes	[1]	hsa-miR-199b-5p	17.0583/1.985800e-02	-20.06
					hsa-miR-199a-3p	16.2084/3.774820e-02	-17.59
2	*IGF1*	*insulin-like growth factor 1 (somatomedin C)*	Granulosa cells, etc	[2]	hsa-let-7b	17.3966/ 2.672260e-02	-22.92
					hsa-miR-23a	17.3748/ 1.593960e-02	-15.48
3	*IGFBP1*	*insulin-like growth factor binding protein 1*	Granulosa cells, etc	[3]	hsa-miR-103	16.7805/ 3.189630e-02	-21.46
					hsa-let-7b	16.1089/ 9.889850e-02	-20.55
					hsa-miR-140-3p	16.1898/ 4.266420e-02	-19.87
4	*CDC2*	*cell division cycle 2, G1 to S and G2 to M*	Oocytes	[1]	hsa-miR-145*	17.1268/ 1.677130e-02	-16.48
5	*CDK2*	*cyclin-dependent kinase 2*	Oocytes	[1]	hsa-miR-15b	15.4162/ 9.063360e-02	-20.1
					hsa-miR-18a	16.431/ 3.872430e-02	-18.94
6	*RB1*	*retinoblastoma 1*	Oocytes	[4]	hsa-miR-143	17.0319/2.137170e-02	-15.72
7	*CUL2*	*cullin 2*	Oocytes	[5]	hsa-miR-15b	15.7993/ 8.761310e-02	-18.57
					hsa-miR-424	18.6368/ 8.779800e-03	-20.05
					hsa-miR-195	16.4184/ 5.003250e-02	-15.64
8	*RAB2*	*RAB2A, member RAS oncogene family*	Oocytes	[6]	rno-miR-21	16.2827/ 3.670530e-02	-16.17
9	*IL2*	*interleukin 2*	Granulosa cells	[7]	hsa-let-7a	17.9651/ 1.121270e-02	-19.61
10	*CX43*	*connexin 43*	Ovary	[8]	dre-miR-23a	17.5667/ 1.993390e-02	-27.51
					dre-miR-23b	17.5667/ 1.962400e-02	-27.51
11	*STAT3*	*signal transducer and activator of transcription 3 (acute-phase response factor)*	Corpus luteum	[9]	hsa-miR-20a	16.8749/ 7.269850e-02	-22.21
					hsa-miR-17	17.1745/ 5.734300e-02	-24.32
12	*DNMT1*	*DNA (cytosine-5-)-methyltransferase 1*	Oocytes	[10]	hsa-miR-145*	17.4956/ 1.155930e-02	-20.44
					hsa-miR-126	13.9965/ 5.771220e-02	-11.07
13	*STAR*	*steroidogenic acute regulatory protein*	Ovary	[11]	hsa-miR-17	17.3682/ 4.979880e-02	-23.81
					hsa-miR-101	15.3062/ 8.480980e-02	-10.38
14	*BMP15*	*bone morphogenetic protein 15*	Oocytes, Ovary	[12, 13]	hsa-miR-145	16.6281/ 3.208670e-02	-22.5
15	*GDF9*	*growth differentiation factor 9*	Oocytes, Ovary		hsa-miR-199a	16.2043/ 3.791010e-02	-20
				[14]	hsa-miR-18a	16.7874/ 2.687100e-02	-19.58
16	*c- MOS*	*c-moloney sarcoma oncogene*	Oocytes	[14]	hsa-miR-101	17.6844/ 9.219050e-03	-23.4
17	*FST*	*follistatin*	Ovary	[13, 15]	hsa-miR-378*	16.5714/ 5.587340e-02	-24.86
					hsa-miR-27b	15.8419/ 8.612300e-02	-17.73
18	*HSPA8*	*heat shock 70kDa protein 8*	Cumulus cells	[16]	hsa-miR-222	18.7465/ 2.378420e-03	-21.04
					hsa-miR-101	17.6034/ 9.952370e-03	-14.77

19	*ZP3*	*zona pellucida glycoprotein 3 (sperm receptor)*	Ovary, Oocyte	[17]	hsa-miR-15b	17.3966/ 2.207880e-02	-18.8
					hsa-miR-940	17.2605/ 3.300140e-02	-28.74
					hsa-miR-140-3p	17.1462/ 1.793330e-02	-21.65
					hsa-miR-29a	17.1462/9.908740e-03	-20.59
20	*ZP1*	*zona pellucida glycoprotein 1 (sperm receptor)*	Oocyte	[18]	hsa-miR-99b*	16.402/ 1.889760e-02	-29.08
					hsa-miR-143	15.8758/ 6.814110e-02	-16.68
21	*EGF*	*epidermal growth factor (beta-urogastrone)*	Luteal cells	[19]	hsa-miR-29a*	17.5947/ 2.185380e-02	-23.88
					hsa-miR-199a-5p	15.6821/ 6.615360e-02	-20.71
22	*ADAMTS8*	*ADAM metallopeptidase with thrombospondin type 1 motif, 8*	Ovary	[20]	mmu-let-7b	17.0975/ 3.394150e-02	-22.15
					mmu-let-7c	17.0975/ 3.052700e-02	-22.15
23	*LH/CG-R*	*luteinizing hormone/choriogonadotropin receptor*	Granulosa cells	[21]	hsa-miR-222	17.5136/ 8.223810e-03	-26.07
					hsa-let-7e	17.432/ 2.644650e-02	-22.97
					hsa-let-7a	16.6305/ 4.700500e-02	-17.15
					hsa-let-7c	16.33/ 7.264700e-02	-17.61
24	*CYP19A1*	*cytochrome P450, family 19, subfamily A, polypeptide 1*	Granulosa cells	[22]	hsa-miR-193a-5p	15.6623/ 9.903720e-02	-22.62
					hsa-miR-23a	16.1244/ 5.094820e-02	-17.43
25	*PTGS2*	*prostaglandin-endoperoxide synthase 2 (prostaglandin G/H synthase and cyclooxygenase)*	Cumulus cells	[23]	bta-let-7c	17.6237/ 3.148530e-02	-15.13
					bta-let-7b	17.3013/ 4.417760e-02	-15.45
					bta-miR-125b	16.4416/ 4.885810e-02	-16.08
26	*PTX3*	*pentraxin-related gene, rapidly induced by IL-1 beta*	Cumulus cells	[24]	hsa-miR-18a	16.575/ 3.341900e-02	-21.09
					hsa-miR-29a	15.9149/ 4.622940e-02	-13.16
27	*TNF*	*tumor necrosis factor (TNF superfamily, member 2)*	Corpus Luteum	[25]	hsa-miR-125b	16.0314/ 5.133550e-02	-21.89
					hsa-miR-409-5p	15.6516/ 3.340670e-02	-13.79
28	*FSHR*	*follicle stimulating hormone receptor*	Granulosa cells	[26]	hsa-miR-101*	17.0555/ 1.374430e-02	-16.94
					hsa-miR-378*	16.1459/ 7.629970e-02	-16.67
					hsa-miR-29a*	16.2596/ 7.866090e-02	-15.94
29	*INHBA*	*inhibin, beta A*	Granulosa cells	[27]	hsa-miR-20a	16.7439/8.003780e	-14.16
					hsa-miR-409-5p	14.8364/7.767180e-02	-11.85
30	*SERPINE2*	*serpin peptidase inhibitor, clade E (nexin, plasminogen activator inhibitor type 1), member 2*	Granulosa Cells	[28]	hsa-miR-424	16.2965/6.588470e-02	-19.14
					hsa-miR-199a-5p	15.9092/5.275390e-02	-23.7
					hsa-miR-199a-3p	15.7742/5.914220e-02	-21.41
31	*LRP8*	*low density lipoprotein receptor-related protein 8*	Granulosa Cells	[29]	hsa-miR-455-5p	16.3337/2.995900e-02	-20.66
32	*EGR1*	*early growth response 1*		[30]	hsa-miR-15b*	17.6609/1.195770e-02	-28.42
33	*ZAR1*	*zygote arrest 1*	Oocyte	[31]	hsa-miR-99a*	16.7144/1.066850e-02	-17.06
					hsa-miR-15b	15.8047/8.720810e-02	-16.47
34	*NR5A2*	*nuclear receptor subfamily 5, group A, member 2*	Ovary	[32]	hsa-miR-125b	15.643 /7.194120e-02	-22.55
35	*POR*	*P450 (cytochrome) oxidoreductase*	Ovary	[32]	hsa-miR-24	19.1953/4.743910e-03	-30.33
36	*BAX*	*BCL2-associated X protein*	Oocyte	[33]	hsa-miR-455-3p	17.3748/1.333010e-02	-19.39

37	*VEGFA*	*vascular endothelial growth factor A*	Ovary	[34]	bta-miR-195	18.2724/1.588310e-02	-25.89
					bta-miR-17-5p	17.7836/1.846720e-02	-22.06
					bta-miR-20a	17.5437/2.635450e-02	-19.91
					bta-miR-145	16.7949/4.182970e-02	-19.54
					bta-miR-26a	16.5526/4.004140e-02	-19.75
					hsa-miR-409-5p	15.7251/4.284640e-02	-23.17
38	*AREG*	*amphiregulin*	Granulosa cells	[35]	hsa-miR-15b	16.8281/ 2.556460e-02	-18.42
					hsa-miR-499-5p	16.1174/ 5.706290e-02	-19.67
39	*ACTB*	*actin, beta*	Oocyte	[31]	hsa-miR-145	16.1202/ 5.265760e-02	-17.81
40	*ALK*	*anaplastic lymphoma receptor tyrosine kinase*	Granulosa cells	[36]	hsa-let-7b*	15.4019/ 6.718860e-02	-13.37
41	*SMAD2*	*SMAD family member 2*	Granulosa cells	[36]	hsa-miR-455-3p	15.8069/ 5.775160e-02	-29.52
42	*HSD3B2*	*hydroxy-delta-5-steroid dehydrogenase, 3 beta- and steroid delta-isomerase 2*	Granulosa cells	[37]	hsa-miR-15b	17.6595/1.753880e-02	-18.31
					hsa-let-7b	17.5498/2.281180e-02	-27.1
					hsa-miR-195	16.7578/3.749650e-02	-16.7
43	*BUB1B*	*BUB1 budding uninhibited by benzimidazoles 1 homolog beta (yeast)*	Oocyte	[38]	hsa-let-7b	19.073/4.680770e-03	-26.76
					hsa-let-7c	18.1859/1.045720e-02	-23.38
					hsa-let-7a	17.5206/1.812050e-02	-21.08
44	*RBL2*	*retinoblastoma-like 2 (p130)*	Oocyte	[38]	hsa-miR-409-5p	15.4722/4.028470e-02	-21.16
45	*HMGA1*	*high mobility group AT-hook 1*	Oocyte	[39]	hsa-miR-26a	16.8505/2.695120e-02	-20.14
46	*SPP1*	*secreted phosphoprotein 1*	Granulosa and theca	[40]	hsa-miR-126	14.7463/2.509870e-02	-17.33
					hsa-miR-127-5p	15.9479/5.980460e-02	-16.53
					hsa-miR-140-3p	16.1391/4.465210e-02	-18.45
47	*AMH*	*anti-Mullerian hormone*	Granulosa cells	[41]	hsa-miR-193a-5p	17.0555/2.414980e-02	-23.85
					hsa-miR-127-3p	15.9185/3.299160e-02	-20.79
48	*Ang II*	*angiogenin, ribonuclease, RNase A family, 5*	Ovary	[42]	hsa-miR-27b*	15.9932/4.726390e-02	-26.58
49	*CCL2*	*chemokine (C-C motif) ligand 2*	Cumulus cells	[43]	hsa-miR-126	15.4742/ 1.108110e-02	-16.51
50	*HOXA9*	*homeo box A9*	Oocyte, granulosa cells	[44]	mmu-miR-145	18.2039/ 1.279040e-02	-17.19
					mmu-miR-143	17.1462/1.989930e-02	-15.35
					bta-miR-101	16.2868/6.084290e-02	-12.72
					mmu-miR-126-3p	15.2496/ 9.831610e-02	-16.18
51	*TBC1D1*	*TBC1 domain family, member 1*		[45]	hsa-miR-125b	15.3968/ 8.889750e-02	-20.98
52	*FOXO3A*	*forkhead box O3*	Ovary	[46]	hsa-miR-99a	16.4709/ 7.738560e-03	-19.67
					hsa-miR-140-3p	16.1446/ 4.443110e-02	-24.48
					hsa-miR-21*	15.741/ 4.794800e-02	-18.48
					hsa-miR-99b	15.6675/ 1.441980e-02	-22.76
					hsa-miR-126	14.2614/ 4.307520e-02	-18.97
53	*CASP3*	*caspase 3, apoptosis-related cysteine peptidase*	Corpus luteum	[47]	hsa-miR-222*	16.0797/ 4.325160e-02	-18.65
					hsa-miR-140-5p	15.984/ 6.188070e-02	-17.04
54	*TGFb1*	*transforming growth factor, beta 1*	Granulosa cells	[48]	hsa-miR-18a	16.2372/ 4.718780e-02	-18.45
					hsa-miR-940	16.2894/ 7.604660e-02	-21.73
					hsa-miR-23a*	17.5122/ 2.752120e-02	-28.23

					hsa-miR-199a-3p	18.2736/ 4.305320e-03	-23.7
55	*CXCR-6*	*chemokine (C-X-C motif) receptor 6*	Granulosa cells	[40]	hsa-miR-199a-5p	17.0501/ 1.663800e-02	-26.42
					hsa-miR-101	15.5701/ 6.665460e-02	-17.78
56	*MMP1*	*matrix metallopeptidase 1 (interstitial collagenase)*	Ovary	[49]	hsa-miR-193a-3p	14.9631/9.596510e-02	-19.42
57	*MMP2*	*matrix metallopeptidase 2*	Ovary	[49]	hsa-miR-26a-1	16.8762/2.169960e-02	-18.71
58	*TNFα*	*tumor necrosis factor (TNF superfamily, member 2)*	Cumulu/granulosa	[50]	dre-miR-23b	17.1692/2.815200e-02	-20.95
59	*LPL*	*lipoprotein lipase*	Corpus luteum	[51]	hsa-miR-15b	16.2422/6.009120e-02	-26.14
60	*AMH*	*anti-Mullerian hormone*	Ovary	[41]	hsa-miR-193a-5p	17.0555/2.414980e-02	-23.85
					hsa-miR-127-3p	15.9185/3.299160e-02	-20.79
61	*wnt4*	*wingless-type MMTV integration site family, member 4*	Ovary	[52]	bta-miR-222	16.0598/4.288560e-02	-20.77
					bta-miR-199a-3p	15.9807/6.758530e-02	-19.14
62	*cxcr4*	*chemokine (C-X-C motif) receptor 4*	Granulosa cells	[40]	hsa-miR-126	13.9793/5.881430e-02	-15.52
63	*cd14*	*CD14 molecule*	Cumulus cells	[50]	hsa-miR-127-5p	16.6007/3.145560e-02	-18.1
					hsa-miR-125b	15.5773/7.613190e-02	-16.45
64	*TLR8*	*toll-like receptor 8*	Cumulus cells	[50]	hsa-miR-27b	17.5857/1.713880e-02	-27.33
					hsa-miR-27a	17.0832/2.415410e-02	-25.57
65	*IGF2R*	*insulin-like growth factor 2 receptor*	Granulosa Cells	[53]	hsa-miR-18a	16.1701/ 5.051580e-02	-16.92
					hsa-miR-143	15.9231/ 6.502460e-02	-19.9
66	*IGFBP4*	*nsulin-like growth factor-binding protein 4 precursor*	Granulosa Cells	[53]	hsa-miR-22	16.5692/ 2.758000e-02	-19.21
					hsa-miR-222	17.5136/ 8.223810e-03	-26.07
67	*lhcgr*	*Lutropin-choriogonadotropic hormone receptor precursor*	Granulosa Cells	[53]	hsa-let-7a	16.6305/ 4.700500e-02	-17.15
					hsa-let-7c	16.33/ 7.264700e-02	-17.61
					hsa-miR-23b	16.0294/ 8.221530e-02	-22.87
68	*TOB1*	*Transducer of erbB-2 1*	Granulosa Cells	[53]	hsa-miR-26a	16.8922/2.594380e-02	-16.61
69	*pdap1*	*PDGFA associated protein 1*	Granulosa Cells	[53]	mmu-miR-145	16.2711/4.793230e-02	-27.26
					mmu-miR-22	16.1373/4.311410e-02	-17.59
70	*GADD45G*	*Growth arrest and DNA-damage- inducible protein GADD45 gamma*	Granulosa Cells	[53]	hsa-miR-103	17.1263/2.300590e-02	-18.4
					hsa-miR-127-3p	16.6675/1.611740e-02	-24.7
					hsa-miR-193a-5p	16.4511/4.483480e-02	-29.36
					hsa-miR-503	15.8338/5.378100e-02	-18.15
					hsa-miR-15b	15.6935/9.578120e-02	-13.6
71	*EDF1*	*Endothelial differentiation-related factor 1*	Granulosa Cells	[53]	hsa-miR-23b	18.0788/1.250420e-02	-26.38
					hsa-miR-29a	17.5103/2.371940e-02	-14.9
					hsa-miR-99a	14.8951/3.899440e-02	-16.67
					hsa-miR-99b	13.8718/8.802760e-02	-19.75
					hsa-miR-193a-3p	17.5062/8.942160e-03	-23.89
					hsa-miR-203	17.6184/2.057350e-02	-15.85
					hsa-miR-143	17.4853/1.348810e-02	-16.89
					hsa-miR-29a	16.7207/2.261010e-02	-15.34
					hsa-miR-127-3p	17.7112/5.900490e-03	-33.71

					hsa-miR-125b	16.7149/2.814470e-02	-22.24
72	*TMEFF1*	*transmembrane protein with EGF-like and two follistatin-like domains 1*	Granulosa Cells	[53]	hsa-miR-101	16.3328/3.287650e-02	-16.14
					hsa-miR-29a	15.4592/6.892840e-02	-14.93
					hsa-miR-27a	17.0034/2.604060e-02	-19.52
					hsa-miR-27b	16.7016/3.913120e-02	-16.28
					hsa-miR-101*	15.8226/4.754970e-02	-16.11
					hsa-miR-101	16.3376/3.272990e-02	-16.14
73	*CTGF*	*connective tissue growth factor*	Granulosa Cells	[53]	hsa-miR-145*	16.0452/4.951250e-02	-15.96
					mmu-miR-7a*	16.4359/3.744620e-02	-19.84
74	*EPS8*	*epidermal growth factor receptor pathway substrate 8*	Granulosa Cells	[53]	mmu-let-7b	16.512/6.260780e-02	-18.57
					mmu-miR-126-3p	14.6154/2.987150e-02	-16.04
75	*TGFB3*	*Transforming growth factor beta-3 precursor*	Granulosa Cells	[53]	hsa-miR-18a	16.7115/4.824610e-02	-24.13
76	*VEGFC*	*Vascular endothelial growth factor C precursor*	Granulosa Cells	[53]	hsa-miR-27a	16.5549/3.966620e-02	-16.83
77	*GRB10*	*Growth factor receptor-bound protein 10*	Granulosa Cells	[53]	hsa-miR-15b	16.0975/6.801110e-02	-18.96
					hsa-miR-424	15.8262/9.768730e-02	-20.06
78	*IGFBP2*	*Insulin-like growth factor-binding protein 2 precursor*	Granulosa Cells	[53]	hsa-miR-940	16.0458/9.340990e-02	-22.35
					hsa-miR-145	15.9944/5.947490e-02	-26.54
					hsa-let-7b*	15.2875/7.404810e-02	-25.93
					hsa-miR-99a	14.2758/7.278800e-02	-16.43
79	*Irs2*	*insulin receptor substrate 2*	Granulosa Cells	[53]	mmu-miR-7b	16.4554/5.691970e-02	-15.97
80	*ADAM17*	*A disintegrin and metalloproteinase domain 17*	Granulosa Cells	[53]	hsa-miR-222	15.728/4.877920e-02	-22.17
					hsa-miR-140-3p	15.6321/7.022190e-02	-18.89
81	*ADAM12*	*A disintegrin and metalloproteinase domain 12*	Granulosa Cells	[53]	hsa-miR-15b	17.7267/1.653470e-02	-28.09
					hsa-miR-23b*	17.1119/3.064470e-02	-27.62
					hsa-miR-195	17.0909/2.820830e-02	-16.47
					hsa-miR-103	16.7283/3.350390e-02	-25.18
82	*ADAMTS1*	*ADAM metallopeptidase with thrombospondin type 1 motif 1*	Granulosa Cells	[53]	mmu-miR-22	15.9859/4.952570e-02	-23.03
83	*RGS1*	*regulator of G-protein signaling 1*			hsa-miR-101	16.8652/1.996050e-02	-13.09
					hsa-miR-222*	16.4731/2.882720e-02	-21.75
					hsa-miR-29a	16.0587/4.071650e-02	-14.78
84	*DDX5*	*DEAD (Asp-Glu-Ala-Asp) box polypeptide 5*	Oocytes	[6]	hsa-miR-18a	16.4041/3.980630e-02	-16.26
					hsa-miR-126	14.554/3.111270e-02	-15.33
85	*TGFBR3*	*transforming growth factor, beta receptor III*		[54]	hsa-miR-103	16.1077/5.989130e-02	-17.15
					hsa-miR-125b	15.3979/8.881980e-02	-19.66
86	*CCND2*	*cyclin D2*	Granulosa cells	[55]	hsa-miR-222	17.4089/1.071940e-02	-17.58
87	*SFRS9*	*splicing factor, arginine/serine-rich 9*		[56]	hsa-miR-29a*	19.2437/4.370020e-03	-15.38
					hsa-miR-27b	17.6386/1.630780e-02	-17.05
					hsa-miR-27a	17.5248/1.591670e-02	-14.91
					hsa-miR-24	17.093/3.121390e-02	-22.05
					hsa-miR-125b	16.1874/4.478590e-02	-15.19
					hsa-miR-99a	15.395/2.341480e-02	-14.7

88	*Casp6*	*caspase 6, apoptosis-related cysteine peptidase*	Ovarian Follicle	[57, 58]	hsa-miR-17	18.1939/2.715510e-02	-24.79
					hsa-miR-20[a]	17.882/ 3.437020e-02	-22.68
89	*Inhbb*	*inhibin, beta B*	Ovarian Follicle	[59]	hsa-miR-22	15.5745/ 6.890290e-02	-22.45
					hsa-miR-18a*	16.7528/ 4.677800e-02	-31.63
90	*Gulo*	*gulonolactone (L-) oxidase*	Ovary	[60]	mmu-miR-29a	16.0229/ 3.995680e-02	-22.04
91	*Cdh2*	*cadherin 2*	Granulosa cells and oocytes	[55]	hsa-miR-199a-5p	16.9266/ 1.886940e-02	-25.85
					hsa-miR-199b-5p	16.3327/ 4.162780e-02	-21.03
92	*Creb3*	*cAMP responsive element binding protein 3*	Granulosa cells	[61]	hsa-miR-29a*	16.8281/ 4.580960e-02	-16.96
					hsa-miR-103	17.0828/ 2.397230e-02	-14.88
93	*Runx1*	*Runt-related transcription factor 1*	Ovary	[62]	hsa-miR-409-3p	16.1294/3.812300e-02	-16.97
94	*Stc1*	*stanniocalcin 1*	Thecal interstitial cells	[22]	mmu-miR-103	16.632/3.938640e-02	-23.92
95	*DNMT3A*	*DNA (cytosine-5)-methyltransferase 3A*	Oocytes	[10]	gga-miR-125b	16.1459/4.217920e-02	-24.38
					hsa-miR-29a	16.7304/2.241400e-02	-23.06
96	*RORA*	*RAR-related orphan receptor A*	Ovary	[63]	hsa-miR-143*	17.624/ 2.741360e-02	-21.82
97	*H1foo*	*H1 histone family, member O, oocyte-specific*	Oocyte	[64]	hsa-miR-125b	15.6019/ 7.453860e-02	-19.88
					hsa-miR-26a	16.402/ 4.050890e-02	-16.77
98	*Hsd11b2*	*hydroxysteroid (11-beta) dehydrogenase 2*	Corpus luteum	[64]	hsa-miR-101	15.8337/ 5.228430e-02	-18.95
					hsa-miR-143*	17.4586/ 3.150310e-02	-28.06
99	*Cspg2*	*Chondroitin □ulphate proteoglycan core protein 2*	Ovary	[64]	rno-miR-143	17.5103/ 1.725090e-02	-13.88
100	*YWHAE*	*tyrosine 3-monooxygenase/tryptophan 5-monooxygenase activation protein, epsilon polypeptide*	Ovary	[65]	hsa-miR-199a-3p	16.3313/ 3.321940e-02	-16.74
					hsa-miR-222	16.3068/ 2.750850e-02	-26.77
101	*MAP3K5*	*mitogen-activated protein kinase kinase kinase 5*	Ovary	[66]	hsa-miR-199a-3p	16.0825/ 4.302150e-02	-13.62
102	*BMP4*	*bone morphogenetic protein 4*	Ovary	[13]	hsa-miR-145	16.0647/ 5.556770e-02	-17.37
103	*SMAD1*	*SMAD family member 1*	Ovary	[13]	hsa-miR-26a	15.5259/ 8.873380e-02	-16.98
104	*SMAD5*	*SMAD family member 5*	Ovary	[13]	rno-miR-29b	15.3821/ 9.549610e-02	-18.62
					rno-miR-101a	15.4237/ 9.712210e-02	-14.39
105	*NOBOX*	*NOBOX oogenesis homeobox*	Oocyte in the newborn ovary	[13]	bta-miR-145	16.0505/ 8.007720e-02	-19.44
					mmu-miR-23a	17.4891/ 1.449400e-02	-17.87
					mmu-miR-23b	17.3748/ 1.552470e-02	-17.87
					mmu-miR-199a	16.487/ 3.185590e-02	-14.21
106	*CtBP1*	*C-terminal binding protein 1*	Steroidogenesis	[67]	mmu-miR-125b	16.3157/ 1.868710e-02	-26.95
					Mmu-miR-409	15.2896/ 7.903530e-02	-14.98
107	*MMP9*	*Matrix metalloproteinase-9 precursor*	Ovary	[68]	hsa-miR-24	16.3733/ 5.891070e-02	-19.96
					mmu-miR-7b	15.9728/ 9.029870e-02	-13.99
					mmu-miR-22	15.7417/ 6.186990e-02	-19.43
108	*IRS1*	*insulin receptor substrate 1*	Theca cells	[69]	mmu-miR-126-3p	13.8597/6.952200e-02	-14.94
					mmu-miR-7b	17.2976/2.506960e-02	-19.15
					mml-miR-125b	15.4465/9.045120e-02	-17.27
					hsa-miR-145	15.603/9.146770e-02	-16.57
					hsa-miR-126	14.3579/.870270e-02	-15.35

109	*Acvr2a*	*Activin receptor type-2A precursor*	Granulosa cells	[36]	rno-miR-15b	18.4755/ 1.103670e-02	-21.98
					rno-miR-29a*	17.1186/ 4.337720e-02	-11.14
110	*Lhx8*	*lim homeodomain transcription factor 8*	Ovarian Development	[70]	hsa-miR-125b		
					hsa-miR-18a	15.635/8.656840e-02	-16.88
					mmu-miR-18a	15.6223/7.542560e-02	-16.62
					dre-miR-145	16.8503/2.912100e-02	-17.44
					dre-miR-29a	16.5177/3.401890e-02	-16.99
111	*Sohlh1*	*spermatogenesis and oogenesis specific basic helix-loop-helix 1*	Ovarian Development	[70]	bta-miR-125b	16.9008/4.307670e-02	-19.66
					hsa-miR-126	14.3149/4.606970e-02	-21.88
					hsa-miR-199a		
					hsa-miR-29a		
112	*CPEB1*	*cytoplasmic polyadenylation element binding protein 1*	Oocyte growth and follicle development	[71]	mmu-miR-29a	15.5525/6.110570e-02	-22.48
					mmu-miR-18a	15.3835/9.611900e-02	-14.28
					mmu-miR-145	16.3382/ 4.498750e-02	-16.1
113	*CPEB2*	*cytoplasmic polyadenylation element binding protein 2*	Oocyte growth and follicle development	[71]	gga-miR-222	15.5373/ 9.313360e-02	-15.08
					mmu-miR-15b	16.6007/ 4.550300e-02	-12.08
					mmu-miR-222	15.7745/ 5.992650e-02	-11.64
114	*CPEB3*	*cytoplasmic polyadenylation element binding protein 3*	Oocyte growth and follicle development	[71]	gga-miR-18a	16.2315/ 6.762780e-02	-23.36
					gga-miR-222	17.2394/ 2.040720e-02	-22.34
					fru-miR-126	14.5653/ 9.138390e-02	-13.35
					bta-miR-222	17.0463/ 1.742610e-02	-21.4
					bta-miR-222	16.3907/ 3.143820e-02	-20.56
115	*IGFBP3*	*Insulin-like growth factor-binding protein 3 precursor*	Oocyte, embryos	[72]	hsa-miR-143	16.4824/3.721040e-02	-21.89
					has-miR-125b	14.3149/4.606970e-02	-16.99

The above table presenting the genes found to be targeted by our cloned miRNAs, which were screened and filtered from total predicted targets based on different condition mentioned in the materials and methods section of the main article. Score/Base P value and energy that were found during target prediction from miRBase target version 5 are listed.

Supplementary Figure 1. Ingenuity analysis of the genes targeted by top eleven screened miRNAs

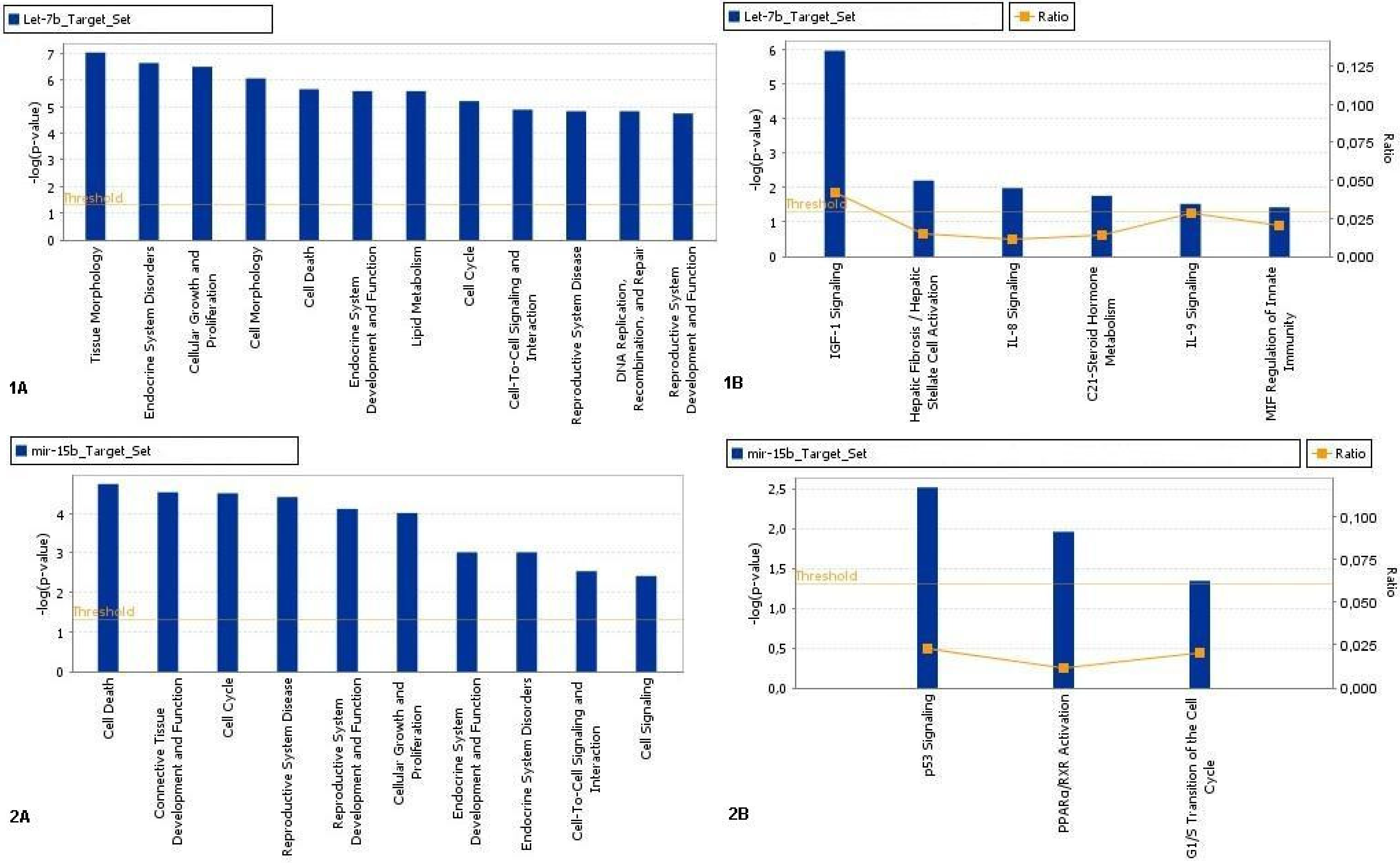

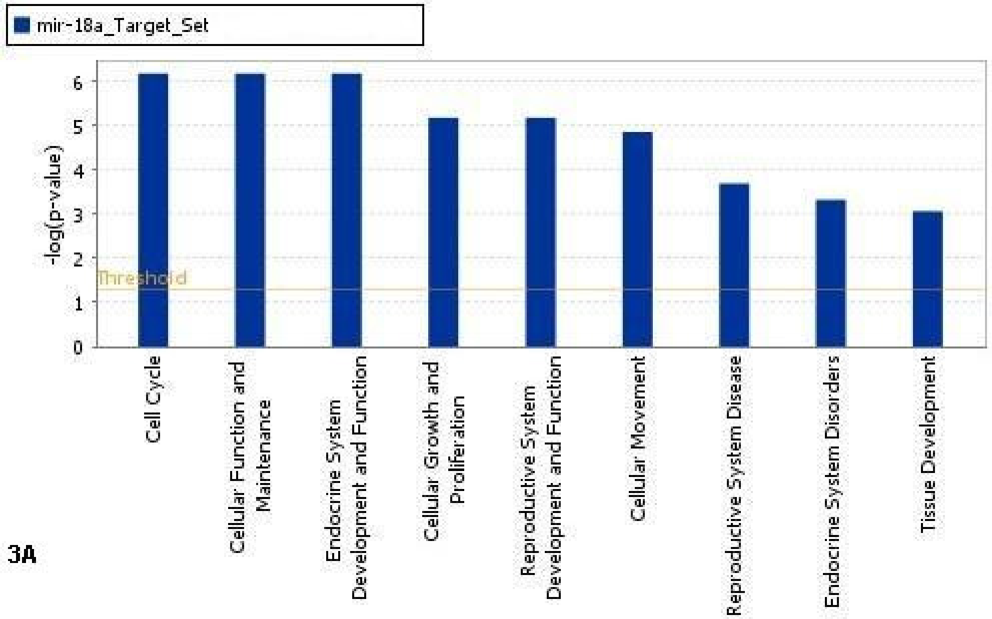
mir-18a_Target_Set
-log(p-value)
0
1
2
3
4
5
6
Threshold
Cell Cycle
Cellular Function and Maintenance
Endocrine System Development and Function
Cellular Growth and Proliferation
Reproductive System Development and Function
Cellular Movement
Reproductive System Disease
Endocrine System Disorders
Tissue Development

3A

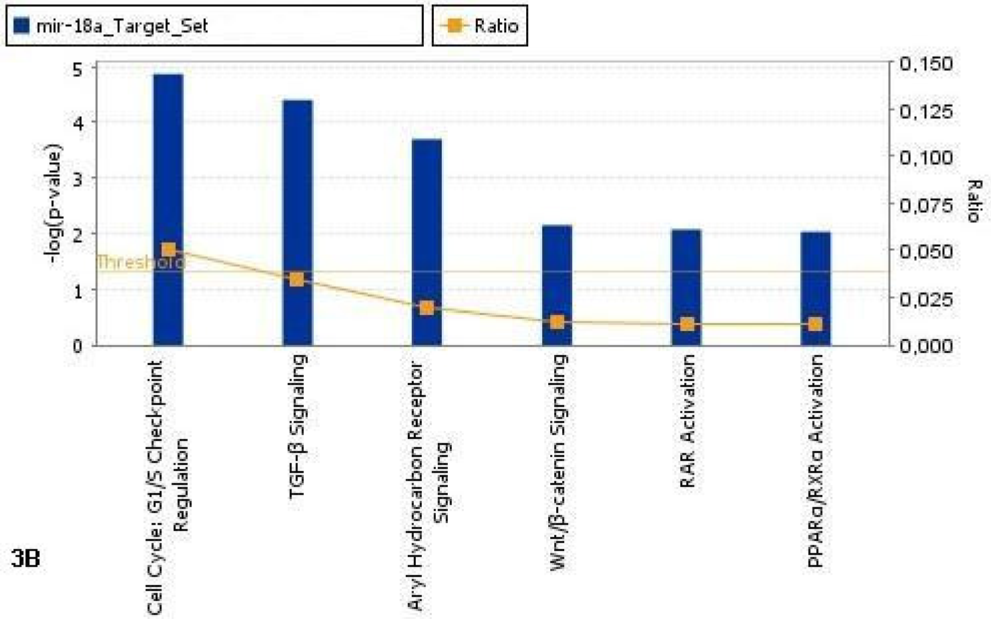
mir-18a_Target_Set
Ratio
-log(p-value)
0
1
2
3
4
5
0,000
0,025
0,050
0,075
0,100
0,125
0,150
Ratio
Threshold
Cell Cycle: G1/S Checkpoint Regulation
TGF-β Signaling
Aryl Hydrocarbon Receptor Signaling
Wnt/β-catenin Signaling
RAR Activation
PPARα/RXRα Activation

3B

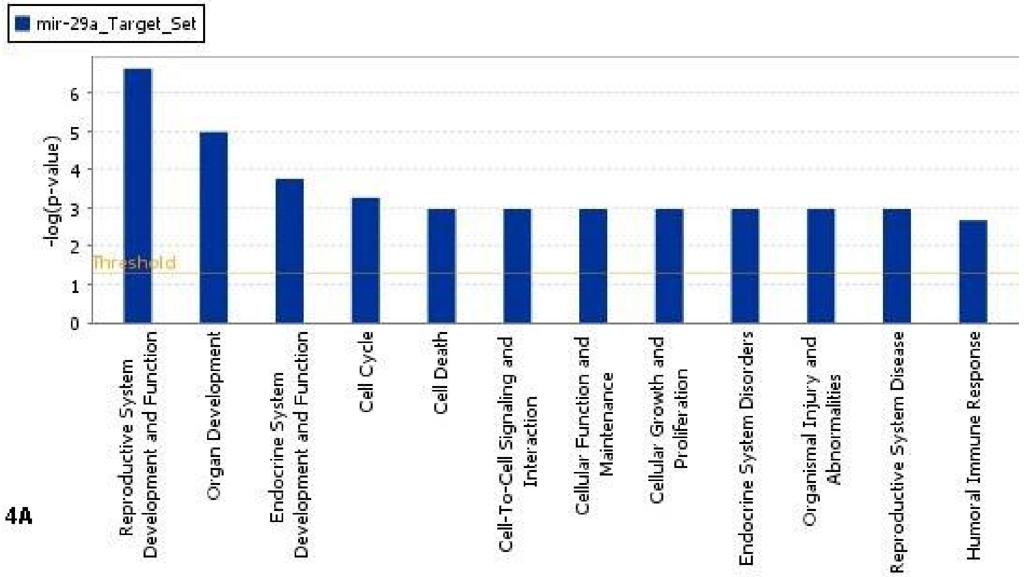
mir-29a_Target_Set
-log(p-value)
0
1
2
3
4
5
6
Threshold
Reproductive System Development and Function
Organ Development
Endocrine System Development and Function
Cell Cycle
Cell Death
Cell-To-Cell Signaling and Interaction
Cellular Function and Maintenance
Cellular Growth and Proliferation
Endocrine System Disorders
Organismal Injury and Abnormalities
Reproductive System Disease
Humoral Immune Response

4A

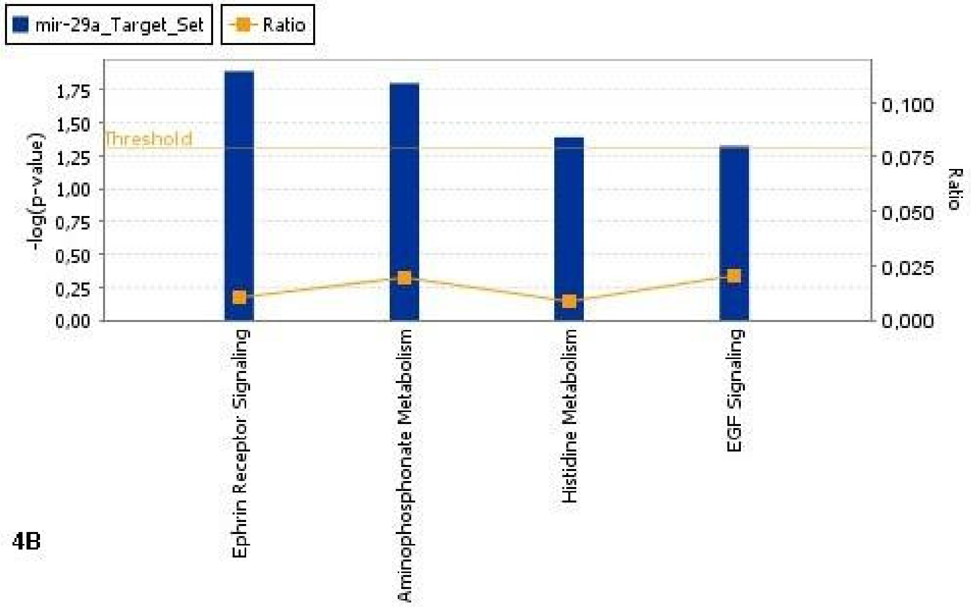
mir-29a_Target_Set
Ratio
-log(p-value)
0,00
0,25
0,50
0,75
1,00
1,25
1,50
1,75
0,000
0,025
0,050
0,075
0,100
Ratio
Threshold
Ephrin Receptor Signaling
Aminophosphonate Metabolism
Histidine Metabolism
EGF Signaling

4B

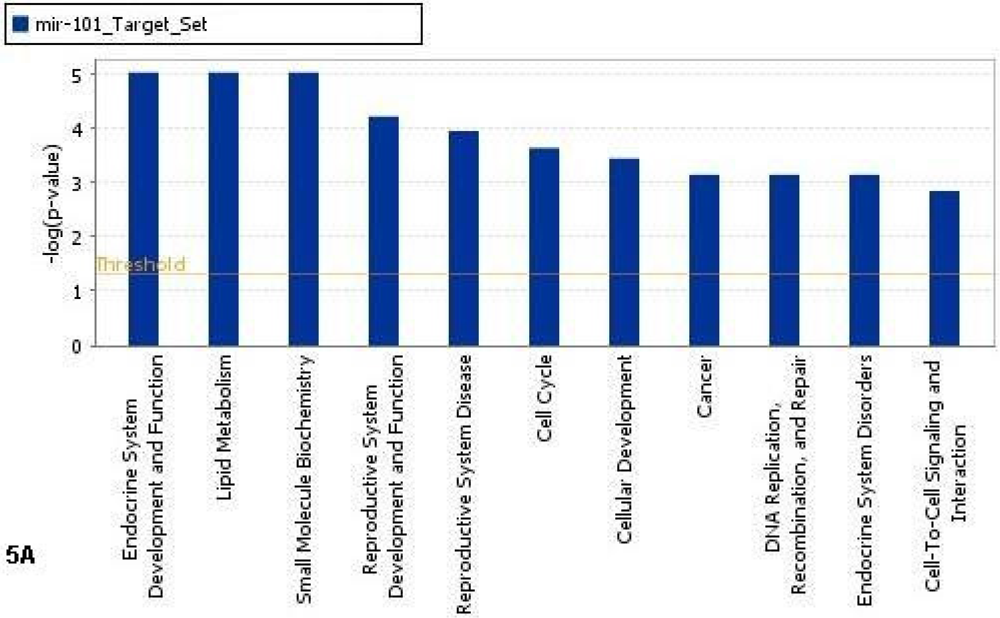
mir-101_Target_Set
-log(p-value)
Threshold
0
1
2
3
4
5
Endocrine System Development and Function
Lipid Metabolism
Small Molecule Biochemistry
Reproductive System Development and Function
Reproductive System Disease
Cell Cycle
Cellular Development
Cancer
DNA Replication, Recombination, and Repair
Endocrine System Disorders
Cell-To-Cell Signaling and Interaction

5A

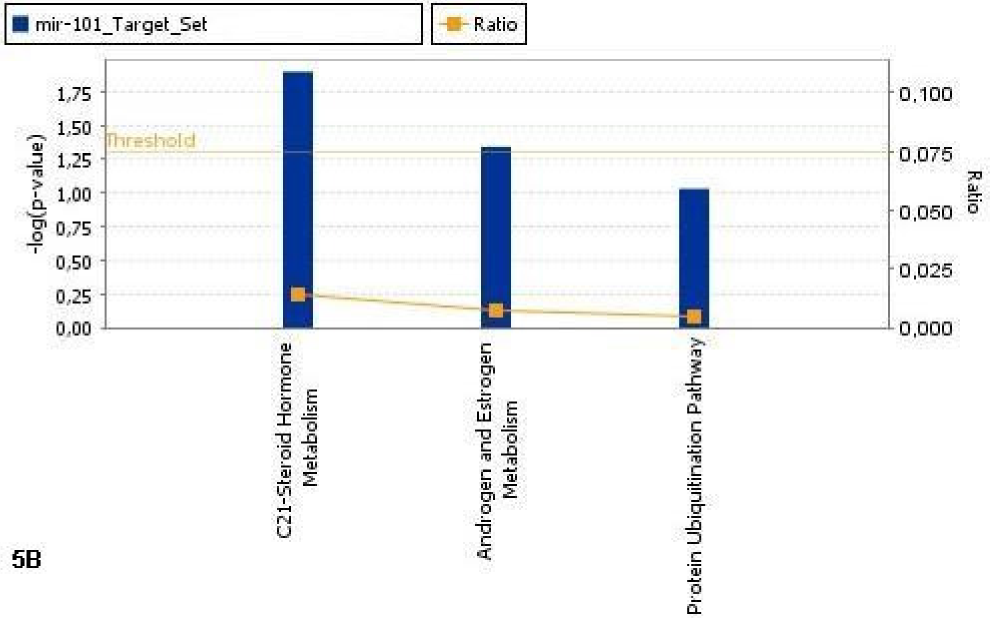
mir-101_Target_Set
Ratio
-log(p-value)
Threshold
0,00
0,25
0,50
0,75
1,00
1,25
1,50
1,75
0,000
0,025
0,050
0,075
0,100
Ratio
C21-Steroid Hormone Metabolism
Androgen and Estrogen Metabolism
Protein Ubiquitination Pathway

5B

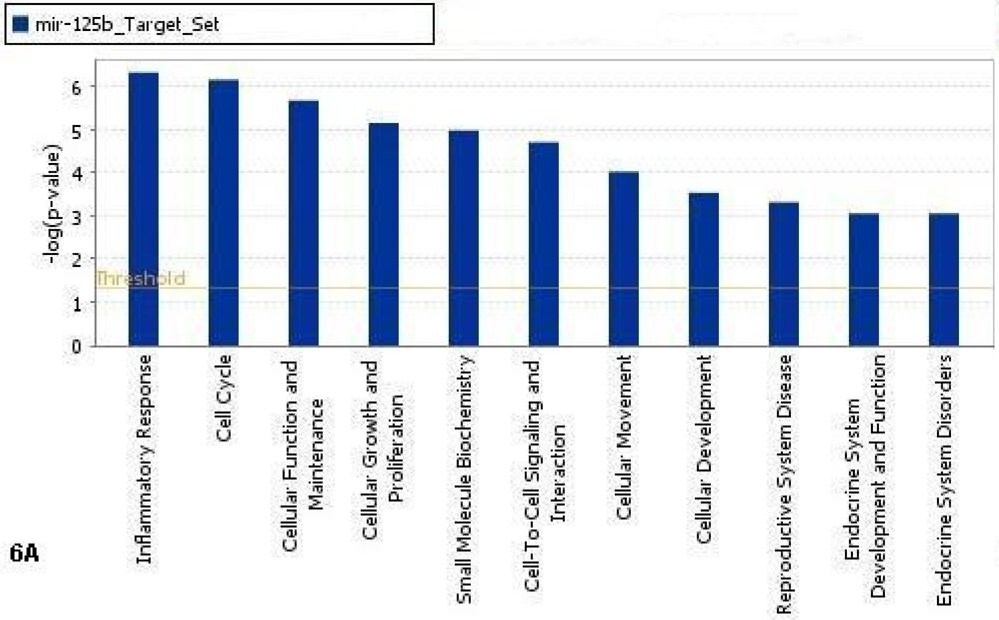
mir-125b_Target_Set
-log(p-value)
Threshold
0
1
2
3
4
5
6
Inflammatory Response
Cell Cycle
Cellular Function and Maintenance
Cellular Growth and Proliferation
Small Molecule Biochemistry
Cell-To-Cell Signaling and Interaction
Cellular Movement
Cellular Development
Reproductive System Disease
Endocrine System Development and Function
Endocrine System Disorders

6A

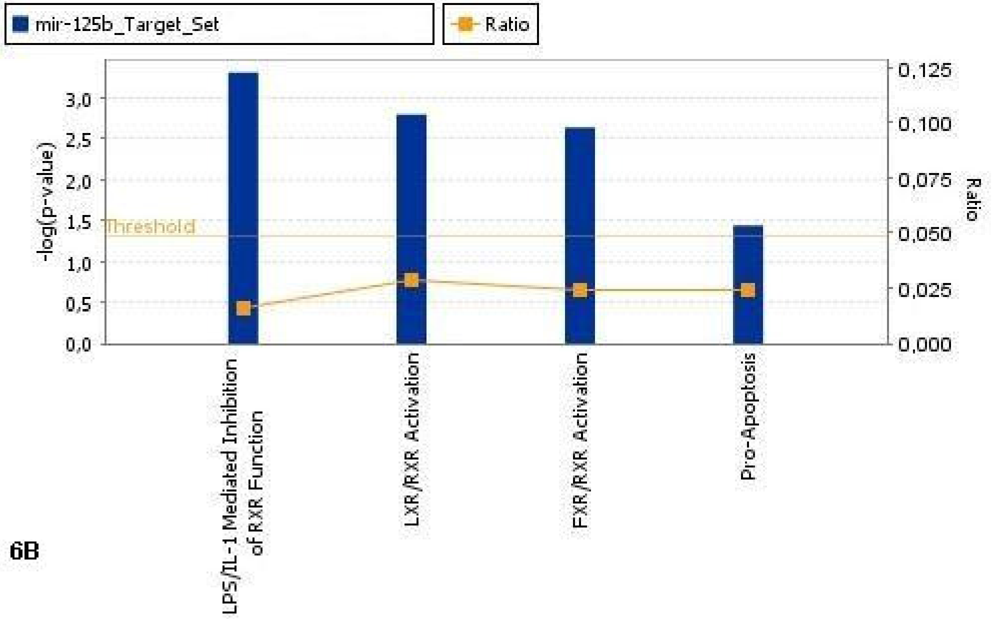
mir-125b_Target_Set
Ratio
-log(p-value)
Threshold
0,0
0,5
1,0
1,5
2,0
2,5
3,0
0,000
0,025
0,050
0,075
0,100
0,125
Ratio
LPS/IL-1 Mediated Inhibition of RXR Function
LXR/RXR Activation
FXR/RXR Activation
Pro-Apoptosis

6B

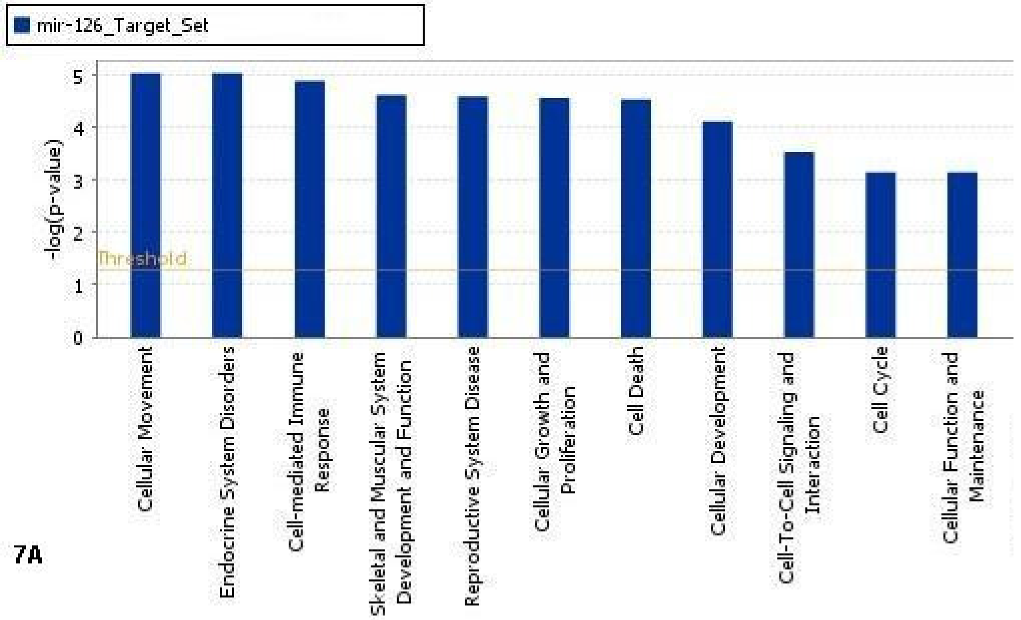
mir-126_Target_Set
-log(p-value)
Threshold
Cellular Movement
Endocrine System Disorders
Cell-mediated Immune Response
Skeletal and Muscular System Development and Function
Reproductive System Disease
Cellular Growth and Proliferation
Cell Death
Cellular Development
Cell-To-Cell Signaling and Interaction
Cell Cycle
Cellular Function and Maintenance

7A

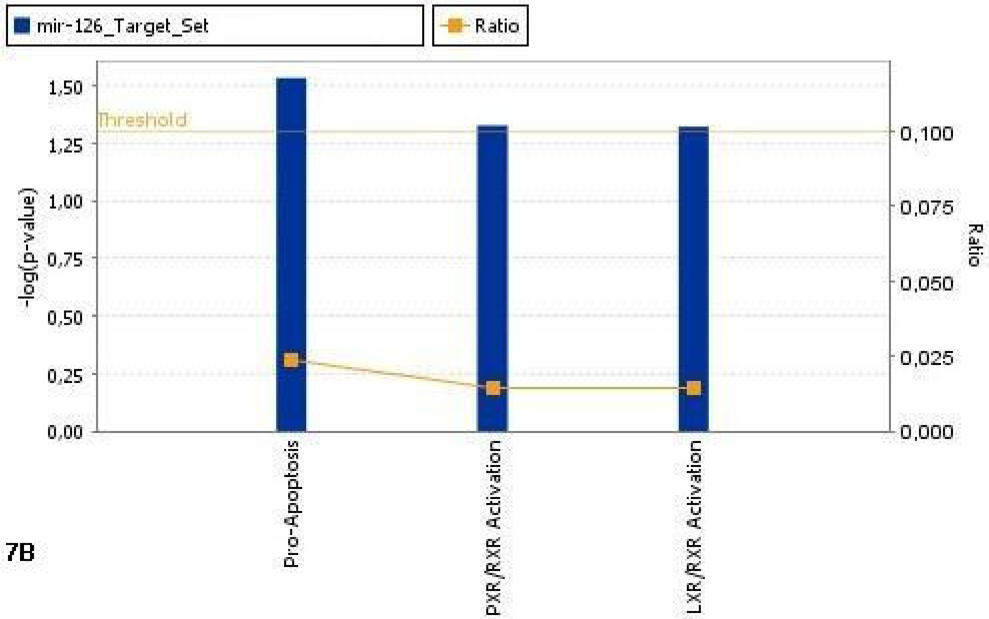
mir-126_Target_Set
Ratio
-log(p-value)
Threshold
Ratio
Pro-Apoptosis
PXR/RXR Activation
LXR/RXR Activation

7B

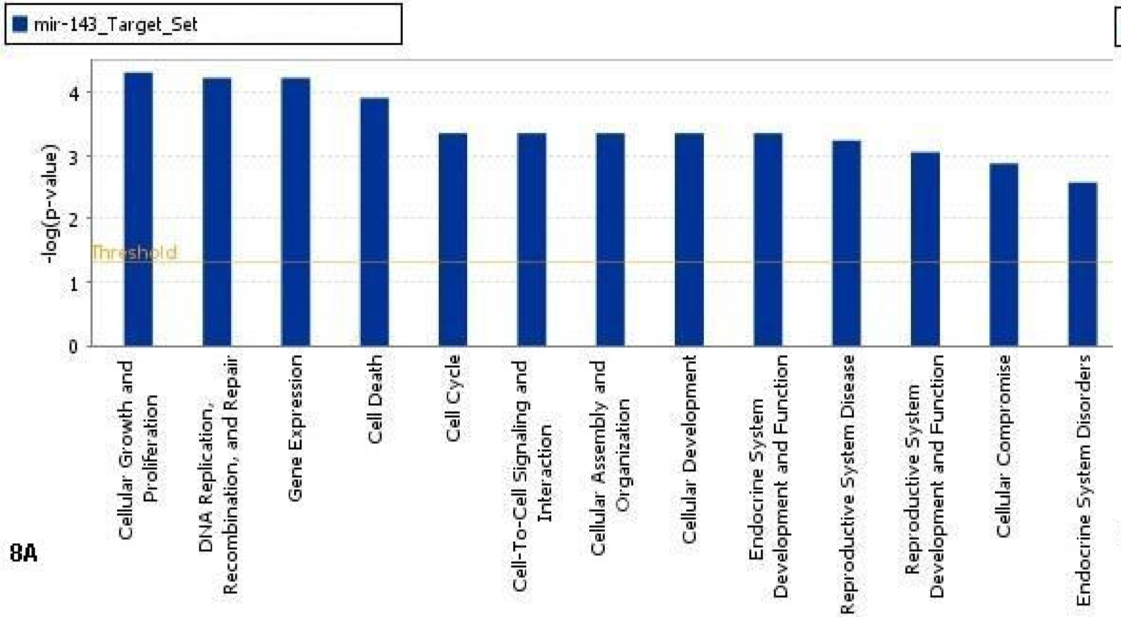
mir-143_Target_Set
-log(p-value)
Threshold
Cellular Growth and Proliferation
DNA Replication, Recombination, and Repair
Gene Expression
Cell Death
Cell Cycle
Cell-To-Cell Signaling and Interaction
Cellular Assembly and Organization
Cellular Development
Endocrine System Development and Function
Reproductive System Disease
Reproductive System Development and Function
Cellular Compromise
Endocrine System Disorders

8A

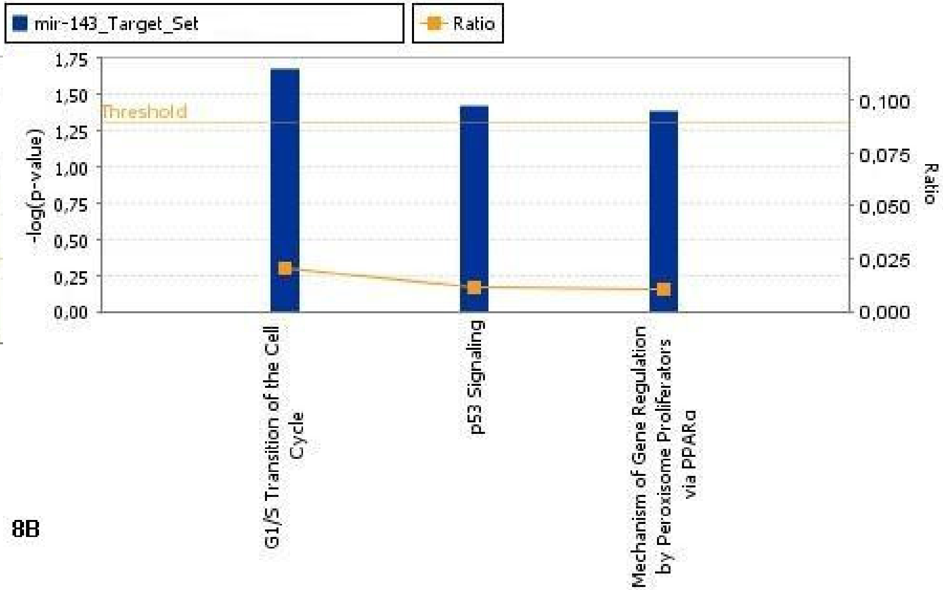
mir-143_Target_Set
Ratio
-log(p-value)
Threshold
Ratio
G1/S Transition of the Cell Cycle
p53 Signaling
Mechanism of Gene Regulation by Peroxisome Proliferators via PPARα

8B

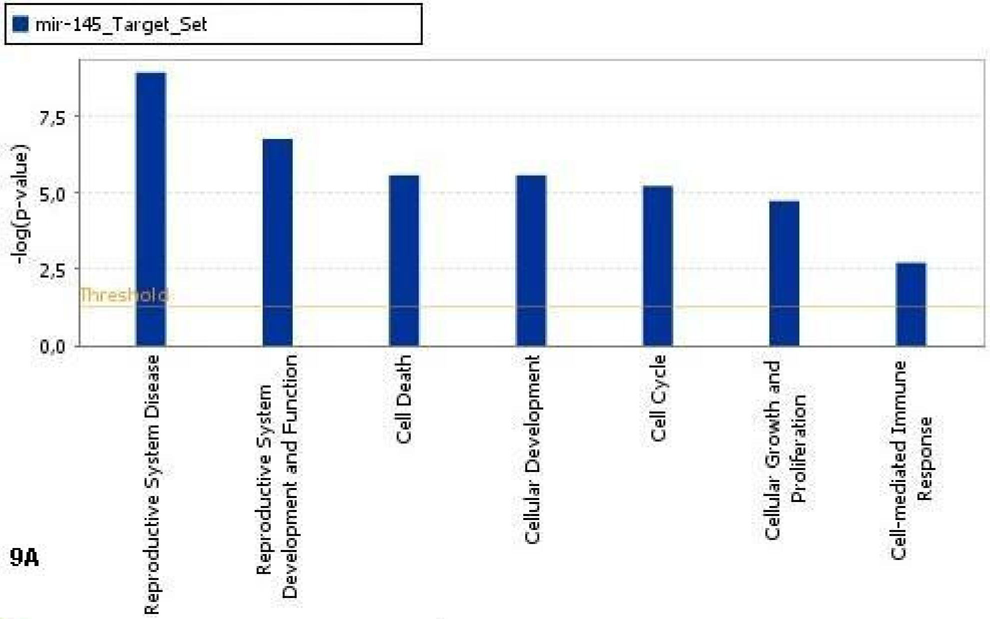
mir-145_Target_Set
-log(p-value)
7,5
5,0
2,5
0,0
Threshold
Reproductive System Disease
Reproductive System Development and Function
Cell Death
Cellular Development
Cell Cycle
Cellular Growth and Proliferation
Cell-mediated Immune Response

9A

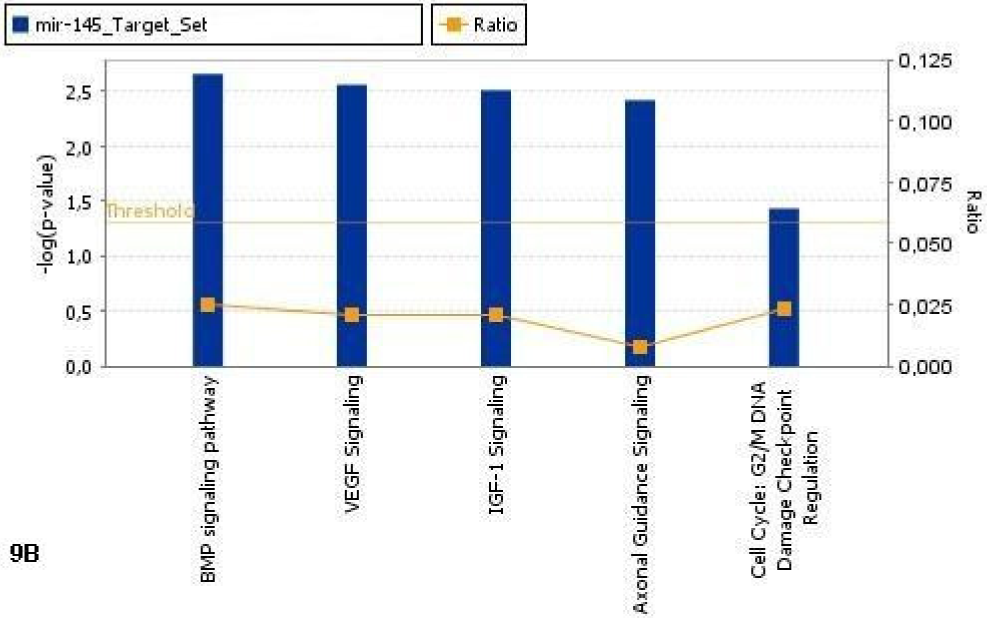
mir-145_Target_Set
Ratio
-log(p-value)
2,5
2,0
1,5
1,0
0,5
0,0
Threshold
0,125
0,100
0,075
0,050
0,025
0,000
Ratio
BMP signaling pathway
VEGF Signaling
IGF-1 Signaling
Axonal Guidance Signaling
Cell Cycle: G2/M DNA Damage Checkpoint Regulation

9B

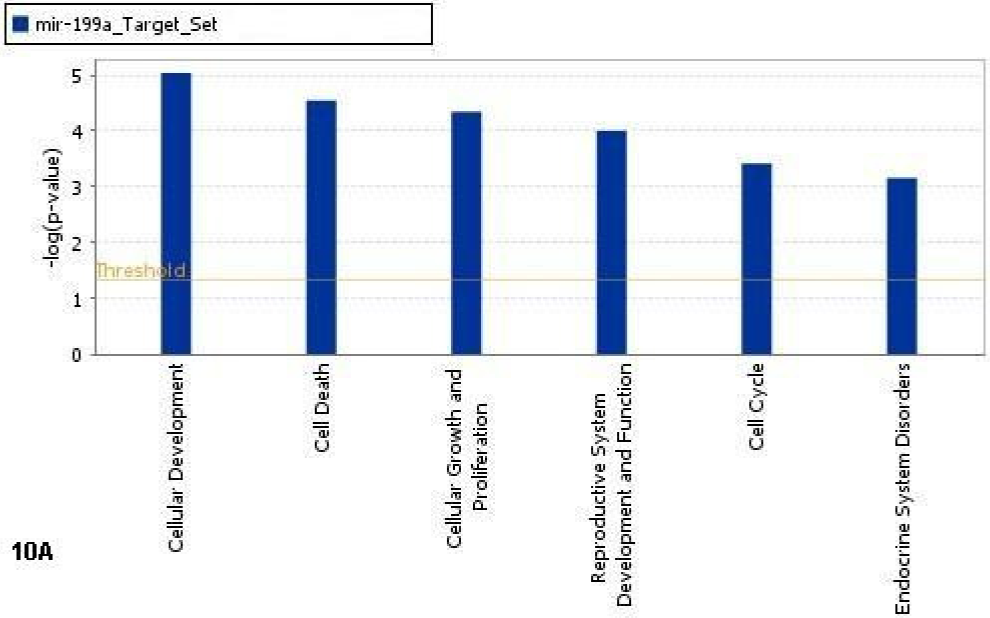
mir-199a_Target_Set
-log(p-value)
5
4
3
2
1
0
Threshold
Cellular Development
Cell Death
Cellular Growth and Proliferation
Reproductive System Development and Function
Cell Cycle
Endocrine System Disorders

10A

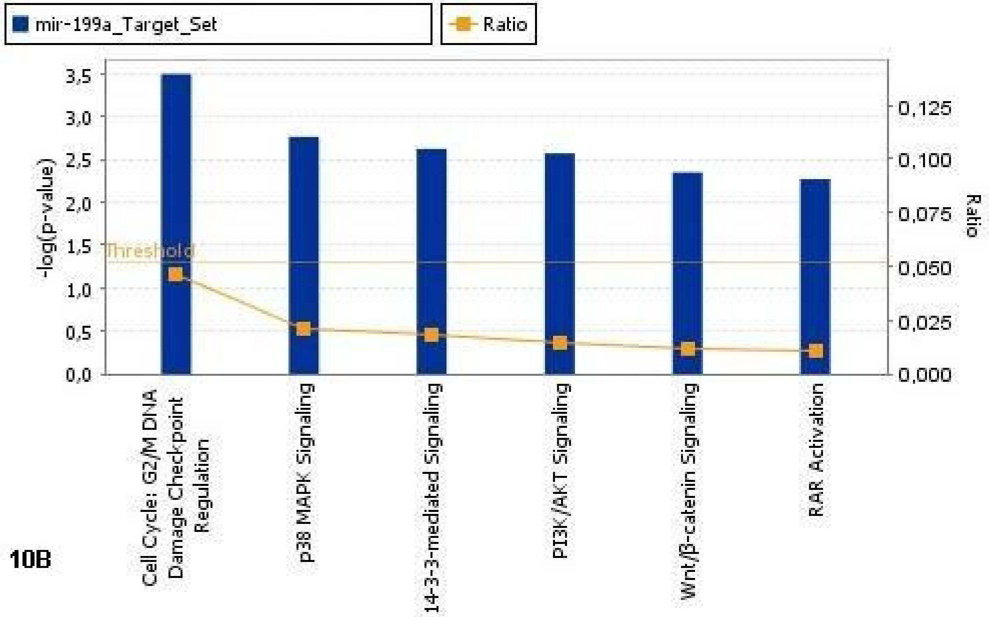
mir-199a_Target_Set
Ratio
-log(p-value)
3,5
3,0
2,5
2,0
1,5
1,0
0,5
0,0
Threshold
0,125
0,100
0,075
0,050
0,025
0,000
Ratio
Cell Cycle: G2/M DNA Damage Checkpoint Regulation
p38 MAPK Signaling
14-3-3-mediated Signaling
PI3K/AKT Signaling
Wnt/β-catenin Signaling
RAR Activation

10B

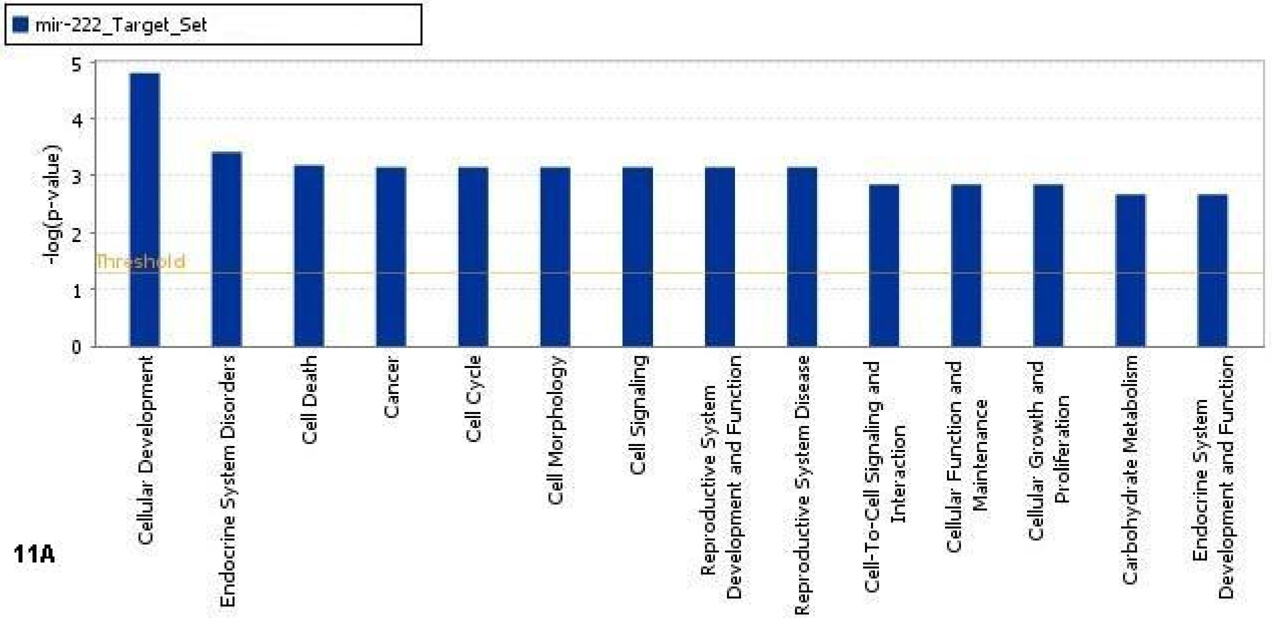
mir-222_Target_Set
-log(p-value)
5
4
3
2
1
0
Threshold
Cellular Development
Endocrine System Disorders
Cell Death
Cancer
Cell Cycle
Cell Morphology
Cell Signaling
Reproductive System Development and Function
Reproductive System Disease
Cell-To-Cell Signaling and Interaction
Cellular Function and Maintenance
Cellular Growth and Proliferation
Carbohydrate Metabolism
Endocrine System Development and Function

11A

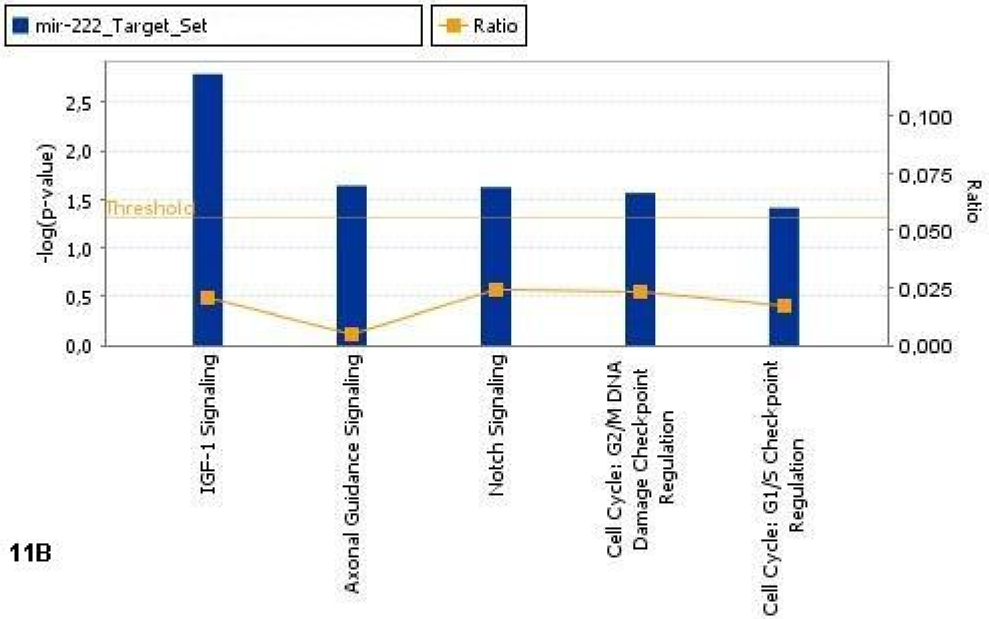
mir-222_Target_Set
Ratio
-log(p-value)
2,5
2,0
1,5
1,0
0,5
0,0
Threshold
0,100
0,075
0,050
0,025
0,000
Ratio
IGF-1 Signaling
Axonal Guidance Signaling
Notch Signaling
Cell Cycle: G2/M DNA Damage Checkpoint Regulation
Cell Cycle: G1/S Checkpoint Regulation

11B

Supp Fig. 1: Detailed Gene Ontology analysis of 11 miRNAs target gene sets by the IPA web delivered software. 1-11 represented the screened target gene set for let-7b, mir-15a, mir-18a, mir-29a, mir-101, mir-125b, mir-126, mir-143, mir- 145, mir-199a, and mir-222. The significant different overrepresented GO functional categories (A) that were found after analysis for the screened target genes sets for each miRNAs (1-11). Whereas, detailed pathway analysis are presented by 'B' with respective miRNAs target gene set number (1-11). Ratio denotes the number of affected genes to total number of genes involved in the pathway. Where, -log(p-value) denotes the threshold $p < 0.05$, which is shown as yellow line. Bars that are above the line indicate significant enrichment of a pathway or function.

Supplementary References:

1. Dalbies-Tran R, Mermillod P: **Use of Heterologous Complementary DNA Array Screening to Analyze Bovine Oocyte Transcriptome and Its Evolution During In Vitro Maturation**. *Biol Reprod* 2003, **68**(1):252-261.
2. Tosca L, Chabrolle C, Crochet S, Tesseraud S, Dupont J: **IGF-1 receptor signaling pathways and effects of AMPK activation on IGF-1-induced progesterone secretion in hen granulosa cells**. *Domest Anim Endocrinol* 2008, **34**(2):204-216.
3. Llewellyn S, Fitzpatrick R, Kenny DA, Murphy JJ, Scaramuzzi RJ, Wathes DC: **Effect of negative energy balance on the insulin-like growth factor system in pre-recruitment ovarian follicles of post partum dairy cows**. *Reproduction* 2007, **133**(3):627-639.
4. Wells D, Bermudez MG, Steuerwald N, Thornhill AR, Walker DL, Malter H, Delhanty JD, Cohen J: **Expression of genes regulating chromosome segregation, the cell cycle and apoptosis during human preimplantation development**. *Hum Reprod* 2005, **20**(5):1339-1348.
5. Mtango NR, Latham KE: **Ubiquitin proteasome pathway gene expression varies in rhesus monkey oocytes and embryos of different developmental potential**. *Physiol Genomics* 2007, **31**(1):1-14.
6. Stanton JL, Green DP: **A set of 840 mouse oocyte genes with well-matched human homologues**. *Mol Hum Reprod* 2001, **7**(6):521-543.
7. Rajagopala Raja C, Spicer L, Stewart R: **Interleukin-2 affects steroidogenesis and numbers of bovine ovarian granulosa cells but not thecal cells in vitro**. *Endocrine* 1995, **3**(12):899-905.
8. Kaiser GG, Kolle S, Boie G, Sinowatz F, Palma GA, Alberio RH: **In vivo effect of growth hormone on the expression of connexin-43 in bovine ovarian follicles**. *Mol Reprod Dev* 2006, **73**(5):600-606.
9. Suter J, Hendry IR, Ndjountche L, Obholz K, Pru JK, Davis JS, Rueda BR: **Mediators of interferon gamma-initiated signaling in bovine luteal cells**. *Biol Reprod* 2001, **64**(5):1481-1486.
10. Lees-Murdock DJ, Lau HT, Castrillon DH, De Felici M, Walsh CP: **DNA methyltransferase loading, but not de novo methylation, is an oocyte-autonomous process stimulated by SCF signalling**. *Dev Biol* 2008, **321**(1):238-250.

11. Murayama C, Miyazaki H, Miyamoto A, Shimizu T: **Involvement of Ad4BP/SF-1, DAX-1, and COUP-TFII transcription factor on steroid production and luteinization in ovarian theca cells**. *Mol Cell Biochem* 2008, **314**(1-2):51-58.
12. Hussein TS, Thompson JG, Gilchrist RB: **Oocyte-secreted factors enhance oocyte developmental competence**. *Dev Biol* 2006, **296**(2):514-521.
13. Roy A, Matzuk MM: **Deconstructing mammalian reproduction: using knockouts to define fertility pathways**. *Reproduction* 2006, **131**(2):207-219.
14. Wu B, Ignotz G, Currie WB, Yang X: **Expression of Mos proto-oncoprotein in bovine oocytes during maturation in vitro**. *Biol Reprod* 1997, **56**(1):260-265.
15. Silva JR, van den Hurk R, van Tol HT, Roelen BA, Figueiredo JR: **Gene expression and protein localisation for activin-A, follistatin and activin receptors in goat ovaries**. *J Endocrinol* 2004, **183**(2):405-415.
16. Tesfaye D, Ghanem N, Fione C, Fair T, Sirard M-A, Hoelker M, Schellander K, Lonergan P: **Gene expression profile of cumulus cells derived from cumulus-oocyte complexes matured either in vivo or in vitro**. *Reprod Fert Dev* 2009, **In Press**.
17. Totzauer I, Kolle S, Sinowatz F, Plendl J, Amselgruber W, Topfer-Petersen E: **Localization of the zona glycoproteins ZPB (ZP3 alpha) and ZPC (ZP3 beta) in the bovine ovary during pre- and postnatal development**. *Ann Anat* 1998, **180**(1):37-43.
18. Choi Y, Yuan D, Rajkovic A: **Germ cell-specific transcriptional regulator sohlh2 is essential for early mouse folliculogenesis and oocyte-specific gene expression**. *Biol Reprod* 2008, **79**(6):1176-1182.
19. Budnik LT, Mukhopadhyay AK: **Epidermal growth factor, a modulator of luteal adenylate cyclase. Characterization of epidermal growth factor receptors and its interaction with adenylate cyclase system in bovine luteal cell membrane**. *J Biol Chem* 1991, **266**(21):13908-13913.
20. Sriraman V, Eichenlaub-Ritter U, Bartsch JW, Rittger A, Mulders SM, Richards JS: **Regulated expression of ADAM8 (a disintegrin and metalloprotease domain 8) in the mouse ovary: evidence for a regulatory role of luteinizing hormone, progesterone receptor, and epidermal growth factor-like growth factors**. *Biol Reprod* 2008, **78**(6):1038-1048.
21. Nogueira MF, Buratini J, Jr., Price CA, Castilho AC, Pinto MG, Barros CM: **Expression of LH receptor mRNA splice variants in bovine granulosa cells: changes with follicle size and regulation by FSH in vitro**. *Mol Reprod Dev* 2007, **74**(6):680-686.
22. Luo CW, Kawamura K, Klein C, Hsueh AJ: **Paracrine regulation of ovarian granulosa cell differentiation by stanniocalcin (STC) 1: mediation through specific STC1 receptors**. *Mol Endocrinol* 2004, **18**(8):2085-2096.
23. Feuerstein P, Cadoret V, Dalbies-Tran R, Guerif F, Bidault R, Royere D: **Gene expression in human cumulus cells: one approach to oocyte competence**. *Hum Reprod* 2007, **22**(12):3069-3077.
24. Zhang X, Jafari N, Barnes RB, Confino E, Milad M, Kazer RR: **Studies of gene expression in human cumulus cells indicate pentraxin 3 as a possible marker for oocyte quality**. *Fertil Steril* 2005, **83 Suppl 1**:1169-1179.
25. Friedman A, Weiss S, Levy N, Meidan R: **Role of tumor necrosis factor alpha and its type I receptor in luteal regression: induction of programmed cell death in bovine corpus luteum-derived endothelial cells**. *Biol Reprod* 2000, **63**(6):1905-1912.
26. Luo W, Wiltbank MC: **Distinct regulation by steroids of messenger RNAs for FSHR and CYP19A1 in bovine granulosa cells**. *Biol Reprod* 2006, **75**(2):217-225.
27. Sisco B, Pfeffer PL: **Expression of activin pathway genes in granulosa cells of dominant and subordinate bovine follicles**. *Theriogenology* 2007, **68**(1):29-37.
28. Fayad T, Levesque V, Sirois J, Silversides DW, Lussier JG: **Gene expression profiling of differentially expressed genes in granulosa cells of bovine dominant follicles using suppression subtractive hybridization**. *Biol Reprod* 2004, **70**(2):523-533.

29. Fayad T, Lefebvre R, Nimpf J, Silversides DW, Lussier JG: **Low-density lipoprotein receptor-related protein 8 (LRP8) is upregulated in granulosa cells of bovine dominant follicle: molecular characterization and spatio-temporal expression studies**. *Biol Reprod* 2007, **76**(3):466-475.
30. Hou X, Arvisais EW, Jiang C, Chen DB, Roy SK, Pate JL, Hansen TR, Rueda BR, Davis JS: **Prostaglandin F2alpha stimulates the expression and secretion of transforming growth factor B1 via induction of the early growth response 1 gene (EGR1) in the bovine corpus luteum**. *Mol Endocrinol* 2008, **22**(2):403-414.
31. Thelie A, Papillier P, Pennetier S, Perreau C, Traverso JM, Uzbekova S, Mermillod P, Joly C, Humblot P, Dalbies-Tran R: **Differential regulation of abundance and deadenylation of maternal transcripts during bovine oocyte maturation in vitro and in vivo**. *BMC Dev Biol* 2007, **7**(125):125.
32. Agca C, Ries JE, Kolath SJ, Kim JH, Forrester LJ, Antoniou E, Whitworth KM, Mathialagan N, Springer GK, Prather RS *et al*: **Luteinization of porcine preovulatory follicles leads to systematic changes in follicular gene expression**. *Reproduction* 2006, **132**(1):133-145.
33. Soto P, Smith LC: **BH4 peptide derived from Bcl-xL and Bax-inhibitor peptide suppresses apoptotic mitochondrial changes in heat stressed bovine oocytes**. *Mol Reprod Dev* 2008, **5**:5.
34. Berisha B, Steffl M, Welter H, Kliem H, Meyer HHD, Schams D, Amselgruber W: **Effect of the luteinising hormone surge on regulation of vascular endothelial growth factor and extracellular matrix-degrading proteinases and their inhibitors in bovine follicles**. *Reproduction, Fertility and Development* 2008, **20**(2):258-268.
35. Yamashita Y, Kawashima I, Yanai Y, Nishibori M, Richards JS, Shimada M: **Hormone-induced expression of tumor necrosis factor alpha-converting enzyme/A disintegrin and metalloprotease-17 impacts porcine cumulus cell oocyte complex expansion and meiotic maturation via ligand activation of the epidermal growth factor receptor**. *Endocrinology* 2007, **148**(12):6164-6175.
36. Miyoshi T, Otsuka F, Suzuki J, Takeda M, Inagaki K, Kano Y, Otani H, Mimura Y, Ogura T, Makino H: **Mutual regulation of follicle-stimulating hormone signaling and bone morphogenetic protein system in human granulosa cells**. *Biol Reprod* 2006, **74**(6):1073-1082.
37. Havelock JC, Smith AL, Seely JB, Dooley CA, Rodgers RJ, Rainey WE, Carr BR: **The NGFI-B family of transcription factors regulates expression of 3beta-hydroxysteroid dehydrogenase type 2 in the human ovary**. *Mol Hum Reprod* 2005, **11**(2):79-85.
38. Gasca S, Pellestor F, Assou S, Loup V, Anahory T, Dechaud H, De Vos J, Hamamah S: **Identifying new human oocyte marker genes: a microarray approach**. *Reprod Biomed Online* 2007, **14**(2):175-183.
39. Vigneault C, McGraw S, Massicotte L, Sirard MA: **Transcription factor expression patterns in bovine in vitro-derived embryos prior to maternal-zygotic transition**. *Biol Reprod* 2004, **70**(6):1701-1709.
40. Skinner MK, Schmidt M, Savenkova MI, Sadler-Riggleman I, Nilsson EE: **Regulation of granulosa and theca cell transcriptomes during ovarian antral follicle development**. *Mol Reprod Dev* 2008, **75**(9):1457-1472.
41. Vigier B, Picard JY, Tran D, Legeai L, Josso N: **Production of anti-Mullerian hormone: another homology between Sertoli and granulosa cells**. *Endocrinology* 1984, **114**(4):1315-1320.
42. Nielsen AH, Hagemann A, Svenstrup B, Nielsen J, Poulsen K: **Angiotensin II receptor density in bovine ovarian follicles relates to tissue renin and follicular size**. *Clin Exp Pharmacol Physiol* 1994, **21**(6):463-469.
43. Tamba S, Yodoi R, Segi-Nishida E, Ichikawa A, Narumiya S, Sugimoto Y: **Timely interaction between prostaglandin and chemokine signaling is a prerequisite for successful fertilization**. *Proc Natl Acad Sci U S A* 2008, **105**(38):14539-14544.

44. Villaescusa JC, Verrotti AC, Ferretti E, Farookhi R, Blasi F: **Expression of Hox cofactor genes during mouse ovarian follicular development and oocyte maturation**. *Gene* 2004, **330**:1-7.
45. Zielak AE, Forde N, Park SD, Doohan F, Coussens PM, Smith GW, Ireland JJ, Lonergan P, Evans AC: **Identification of novel genes associated with dominant follicle development in cattle**. *Reprod Fertil Dev* 2007, **19**(8):967-975.
46. Castrillon DH, Miao L, Kollipara R, Horner JW, DePinho RA: **Suppression of ovarian follicle activation in mice by the transcription factor Foxo3a**. *Science* 2003, **301**(5630):215-218.
47. Nishimura R, Komiyama J, Tasaki Y, Acosta TJ, Okuda K: **Hypoxia promotes luteal cell death in bovine corpus luteum**. *Biol Reprod* 2008, **78**(3):529-536.
48. Zheng X, Price CA, Tremblay Y, Lussier JG, Carriere PD: **Role of transforming growth factor-beta1 in gene expression and activity of estradiol and progesterone-generating enzymes in FSH-stimulated bovine granulosa cells**. *Reproduction* 2008, **136**(4):447-457.
49. Liu K, Wahlberg P, Ny T: **Coordinated and cell-specific regulation of membrane type matrix metalloproteinase 1 (MT1-MMP) and its substrate matrix metalloproteinase 2 (MMP-2) by physiological signals during follicular development and ovulation**. *Endocrinology* 1998, **139**(11):4735-4738.
50. Shimada M, Hernandez-Gonzalez I, Gonzalez-Robanya I, Richards JS: **Induced expression of pattern recognition receptors in cumulus oocyte complexes: novel evidence for innate immune-like functions during ovulation**. *Mol Endocrinol* 2006, **20**(12):3228-3239.
51. Shemesh M, Bensadoun A, Hansel W: **Lipoprotein lipase activity in the bovine corpus luteum during the estrous cycle and early pregnancy**. *Proc Soc Exp Biol Med* 1976, **151**(4):667-669.
52. Vainio S, Heikkila M, Kispert A, Chin N, McMahon AP: **Female development in mammals is regulated by Wnt-4 signalling**. *Nature* 1999, **397**(6718):405-409.
53. Ben-Ami I, Freimann S, Armon L, Dantes A, Ron-El R, Amsterdam A: **Novel function of ovarian growth factors: combined studies by DNA microarray, biochemical and physiological approaches**. *Mol Hum Reprod* 2006, **12**(7):413-419.
54. Sarraj MA, Chua HK, Umbers A, Loveland KL, Findlay JK, Stenvers KL: **Differential expression of TGFBR3 (betaglycan) in mouse ovary and testis during gonadogenesis**. *Growth Factors* 2007, **25**(5):334-345.
55. Sicinski P, Donaher JL, Geng Y, Parker SB, Gardner H, Park MY, Robker RL, Richards JS, McGinnis LK, Biggers JD *et al*: **Cyclin D2 is an FSH-responsive gene involved in gonadal cell proliferation and oncogenesis**. *Nature* 1996, **384**(6608):470-474.
56. Mihm M, Baker PJ, Fleming LM, Monteiro AM, O'Shaughnessy PJ: **Differentiation of the bovine dominant follicle from the cohort upregulates mRNA expression for new tissue development genes**. *Reproduction* 2008, **135**(2):253-265.
57. Valdez KE, Cuneo SP, Turzillo AM: **Regulation of apoptosis in the atresia of dominant bovine follicles of the first follicular wave following ovulation**. *Reproduction* 2005, **130**(1):71-81.
58. Yacobi K, Tsafriri A, Gross A: **Luteinizing hormone-induced caspase activation in rat preovulatory follicles is coupled to mitochondrial steroidogenesis**. *Endocrinology* 2007, **148**(4):1717-1726.
59. de Kretser DM, Robertson DM: **The isolation and physiology of inhibin and related proteins**. *Biol Reprod* 1989, **40**(1):33-47.
60. Tsuji M, Ito Y, Terada N, Mori H: **Ovarian aromatase activity in scorbutic mutant rats unable to synthesize ascorbic acid**. *Acta Endocrinol (Copenh)* 1989, **121**(4):595-602.
61. Chen YJ, Hsiao PW, Lee MT, Mason JI, Ke FC, Hwang JJ: **Interplay of PI3K and cAMP/PKA signaling, and rapamycin-hypersensitivity in TGFbeta1 enhancement of FSH-stimulated steroidogenesis in rat ovarian granulosa cells**. *J Endocrinol* 2007, **192**(2):405-419.

62. Jo M, Curry TE, Jr.: **Luteinizing hormone-induced RUNX1 regulates the expression of genes in granulosa cells of rat periovulatory follicles**. *Mol Endocrinol* 2006, **20**(9):2156-2172.
63. Guastavino JM, Boufares S, Crusio WE: **Ovarian abnormalities in the staggerer mutant mouse**. *ScientificWorldJournal* 2005, **5**:661-664.
64. Hennebold JD: **Characterization of the ovarian transcriptome through the use of differential analysis of gene expression methodologies**. *Hum Reprod Update* 2004, **10**(3):227-239.
65. van Hemert MJ, Steensma HY, van Heusden GP: **14-3-3 proteins: key regulators of cell division, signalling and apoptosis**. *Bioessays* 2001, **23**(10):936-946.
66. Sirotkin AV, Benco A, Tandlmajerova A, Vasicek D, Kotwica J, Darlak K, Valenzuela F: **Transcription factor p53 can regulate proliferation, apoptosis and secretory activity of luteinizing porcine ovarian granulosa cell cultured with and without ghrelin and FSH**. *Reproduction* 2008, **136**(5):611-618.
67. Fiedler SD, Carletti MZ, Hong X, Christenson LK: **Hormonal regulation of MicroRNA expression in periovulatory mouse mural granulosa cells**. *Biol Reprod* 2008, **79**(6):1030-1037.
68. Robker RL, Russell DL, Yoshioka S, Sharma SC, Lydon JP, O'Malley BW, Espey LL, Richards JS: **Ovulation: a multi-gene, multi-step process**. *Steroids* 2000, **65**(10-11):559-570.
69. Yen HW, Jakimiuk AJ, Munir I, Magoffin DA: **Selective alterations in insulin receptor substrates-1, -2 and -4 in theca but not granulosa cells from polycystic ovaries**. *Mol Hum Reprod* 2004, **10**(7):473-479.
70. Pangas SA, Choi Y, Ballow DJ, Zhao Y, Westphal H, Matzuk MM, Rajkovic A: **Oogenesis requires germ cell-specific transcriptional regulators Sohlh1 and Lhx8**. *Proc Natl Acad Sci U S A* 2006, **103**(21):8090-8095.
71. Racki WJ, Richter JD: **CPEB controls oocyte growth and follicle development in the mouse**. *Development* 2006, **133**(22):4527-4537.
72. Wang TH, Chang CL, Wu HM, Chiu YM, Chen CK, Wang HS: **Insulin-like growth factor-II (IGF-II), IGF-binding protein-3 (IGFBP-3), and IGFBP-4 in follicular fluid are associated with oocyte maturation and embryo development**. *Fertil Steril* 2006, **86**(5):1392-1401.
