# Supplementary material for: Identification and characterization of miRNAs expressed in the bovine ovary
Source: BMC Genomics. 2009 Sep 18;10:443. doi: 10.1186/1471-2164-10-443 (PMC2762473; doi:10.1186/1471-2164-10-443)
Supplement: Additional file 4 — List of oligos and primers used for this study. [file 1471-2164-10-443-S4.PDF]

Sl No.	Name[A]	Sequence (5´-3´)	Usage
1.	3´ Linker	rAppCTGTAGGCACCATCAAT/3ddC	Library
2.	RT Primer	GATTGATGGTGCCTACAG	Library
3.	2nd 3´Linker	rAppTGGAATTCTCGGGTGCCAAGGT/ddC	Library
4.	PCR Primer	CCTTGGCACCCGAGAATT	Library
5.	M13 Forward	GTAAAACGACGGCCAG	Sequencing
6.	M13 reverse	CAGGAAACAGCTATGAC	Sequencing
7.	RTQ1	CGAATTCTAGAGCTCGAGGCAGGCGACATGGCT GGCTAGTTAAGCTTGGTACCGAGCTCGGATCCA CTAGTCC$(T)_{25}^{VN}$	RT PCR
8.	RTQ2	CGAATTCTAGAGCTCGAGGCAGGCGACATG (T)25^{VN}	RT PCR
9.	RTQ-UNI	CGAATTCTAGAGCTCGAGGCAGG	RT PCR
10.	Bta-Let7b	TGAGGTAGTAGGTTGTGTGGTT	RT PCR
11.	Bta-miR-15b	GTAAACCATGATGTGCTGCTA	RT PCR
12.	Bta-miR-18a	ATCTGCACTAGATGCACCT	RT PCR
13.	Bta-miR-29a	AACCGATTTCAGATGGTGCTA	RT PCR
14.	Bta-miR-101	TTCAGTTATCACAGTACTGTA	RT PCR
15.	Bta-miR-125b	TCACAAGTTAGGGTCTCAGGGA	RT PCR
16.	Bta-miR-126	CGCATTATTACTCACGGTACG	RT PCR
17.	Bta-miR-145	GTCCAGTTTTCCCAGGAATCC	RT PCR
18.	Bta-miR-199a	TAACCAATGTGCAGACTACTGT	RT PCR
19.	Bta-miR-222	ACCCAGTAGCCAGATGTAGCT	RT PCR
20.	Bomir-22/22*	ACAGTTCTTCAACTGGCAGCTT	RT PCR
21.	Bomir-140/140*	CAGTGGTTTTACCCTATGGTAG	RT PCR
22.	Bomir-143:	TGAGATGAAGCACTGTAGCTC	RT PCR
23.	Bomir-152	CCAAGTTCTGTCATGCACTGA	RT PCR
24.	Bomir-193a	GGGACTTTGTAGGCCAGTT	RT PCR
25.	Bomir-378	CTGGACTTGGAGTCAGAAGGC	RT PCR
26.	Bomir-382	GAATCCACCACGAACAACTTC	RT PCR
27.	Bomir-409	AGGGGTTCACCGAGCAACAT	RT PCR
28.	Bomir-424	CAAAACGTGAGGCGCTGCTA	RT PCR
29.	Bomir-503	TGCAGTACTGTTCCCGCTGCTA	RT PCR
30.	Bomir-542	TCTCGTGACATGATGATCCCCGA	RT PCR
31.	Bomir-578	TGTGGGTGTGTGCATGTGCGTG	RT PCR
32.	Bomir-652	CACAACCCTAGTGGCGCCATT	RT PCR
33.	Bomir-940	GCAGGGCCCCCGCTCCCC	RT PCR
34.	Bomir-A4052	GGGAGCCTCGGTTGGCCTCGG	RT PCR
35.	Bomir-A3341	GTGGCTGTCCCTGGAGGTGGG	RT PCR
36.	Bomir-C2841	GCCCCGGCCGCTCCCGGCC	RT PCR
37.	Bomir-E2664	AGGGCGGGCGGCGACTGGAA	RT PCR
38.	Bomir-G2511	AGGCGGGCCGGGGTTGGAAGG	RT PCR
39.	Bomir-F2531	TGGTGGAGATGCCGGGGACGT	RT PCR
40.	Bomir-A2143	CGGCAGATGAAGTCCATCGG	RT PCR
41.	Bomir-C1931	CCTGCTGATCTCACATTAATT	RT PCR
42.	Bomir-F1821	AGCCCTGGCCCTGCCATCGTG	RT PCR
43.	Bomir-C1511	GTGGAGGAGAATGCCCGGGG	RT PCR

44.	Bomir-D1431	GGCGACGGAGGCGCGACCCCC	RT PCR
45.	Bomir-F1353	ATCTTTGGGCTAGGTTAGTTC	RT PCR
46.	Bomir-F1351	GCCCCGGCCGCTCCCGGCCTT	RT PCR
47.	Bomir-A3711	TTCCGCGCTCTACGCCAGC	RT PCR
48.	Bomir-F0131	GGGGCGGGGGGGCGGGTG	RT PCR
49.	Bomir-F0132	AGCCCGGGCCCCTCCCCTG	RT PCR
50.	Bomir-H0121	ACTTCCCGTGTGTTGAGCC	RT PCR
51.	Bomir-F0244	GCTACTACCGATTGGATGG	RT PCR
52.	Bomir-H0222	CGGCGGCAGCGCCGGGGC	RT PCR
53.	Bomir-A0321	AGCGCCGCCGGCCGCACC	RT PCR
54.	Bomir-C0533	CGGGACCGGGGTCCGGTGC	RT PCR
55.	Bomir-F0522	GGTGGGGTGGGGGGGTTGG	RT PCR
56.	Bomir-B0821	GTCCCCGGGGCTCCCGCC	RT PCR
57.	Bomir-F2422	GGTGGGAGGGTCCCACCGAG	RT PCR
58.	Bomir-D3011	CCGAGTGCTCCCGCGAGCGCT	RT PCR

[VN]: Two variable neuceotides, where V is A, G, or C; N is A, G, C, or T

[A]: Name started with Bta and Bomir denotes annotated and new miRNAs bovine miRNAs, respectively.
